# Supplementary material for: Screening drug effects in patient‐derived cancer cells links organoid responses to genome alterations
Source: Mol Syst Biol. 2017 Nov 27;13(11):955. doi: 10.15252/msb.20177697 (PMC5731348; doi:10.15252/msb.20177697)
Supplement: Supplementary file 1 — Appendix [file MSB-13-955-s001.pdf]

Appendix for:

Screening drug effects in patient-derived cancer cells links organoid responses to genome alterations

Authors:

Julia Jabs[1,2], Franziska M. Zickgraf[3,4], Jeongbin Park[1], Steve Wagner[3,4], Xiaoqi Jiang[5], Katharina Jechow[1,2], Kortine Kleinheinz[1], Umut H. Toprak[1], Marc A. Schneider[6], Michael Meister[6], Saskia Spaich[7], Marc Sütterlin[7], Matthias Schlesner[1], Andreas Trumpp[3,4,8], Martin Sprick[3,4], Roland Eils[1,2,9]*, Christian Conrad[1,2]*

Table of Contents:

Appendix Figure S1 - Similar cell death ratio determined from single confocal images and maximum intensity projections……………………………………………………………………..**2**

Appendix Figure S2 - Cancer organoids from patient derived OC cells retain tumour markers CA-125 and WT1……………………………………………………………...............................**3**

Appendix Figure S3 - Drug-induced growth arrest and cell death increase in ovarian cancer organoids with time………………………………………………………………………………**4**

Appendix Figure S4 - Homologous recombination deficiency scores and cell growth.................**5**

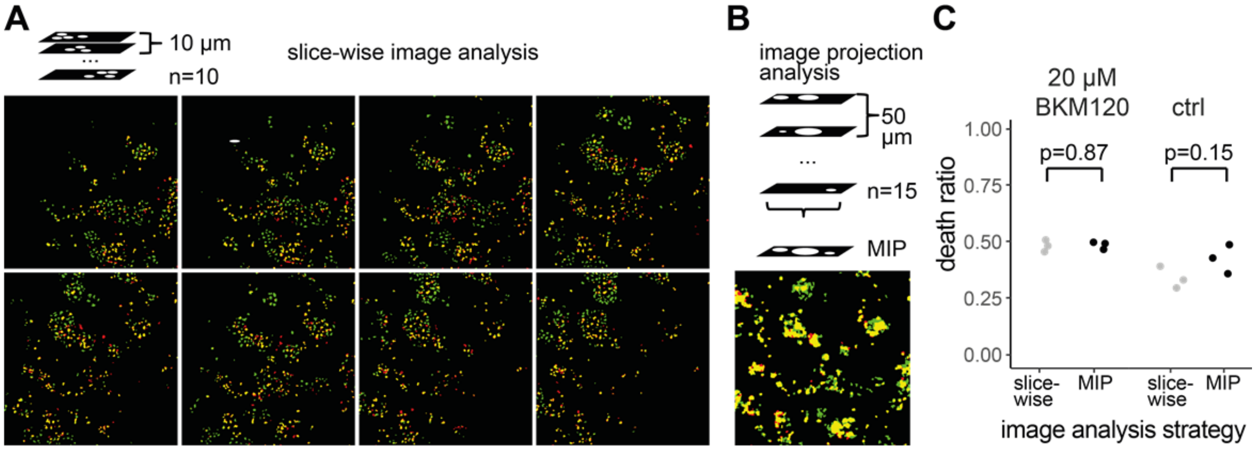

Appendix Figure S1 - Similar cell death ratio determined from single confocal images and maximum intensity projections.

A. Representative confocal images (binary) with 10 µm distance used for slice-wise image analysis. OC12 organoids at day 7 were stained with Hoechst (green) and PI (red).

B. Maximum intensity projection (MIP) of a confocal image stack with 50 µm slice distance taken at the same x,y position as images in A.

C. Analysis of single confocal images or MIPs yields similar death ratio. OC12 organoids were treated with 20 µM BKM120 or left untreated (ctrl) for 72 h and imaged as described in A,B. In slice-wise image analysis, areas of dead and all cells determined from each slice were summarized and used to calculate the average cell death ratio. Data points derive from images acquired at different positions (wells) within the same experiment. Two-sided Welch's *t*-test was performed.

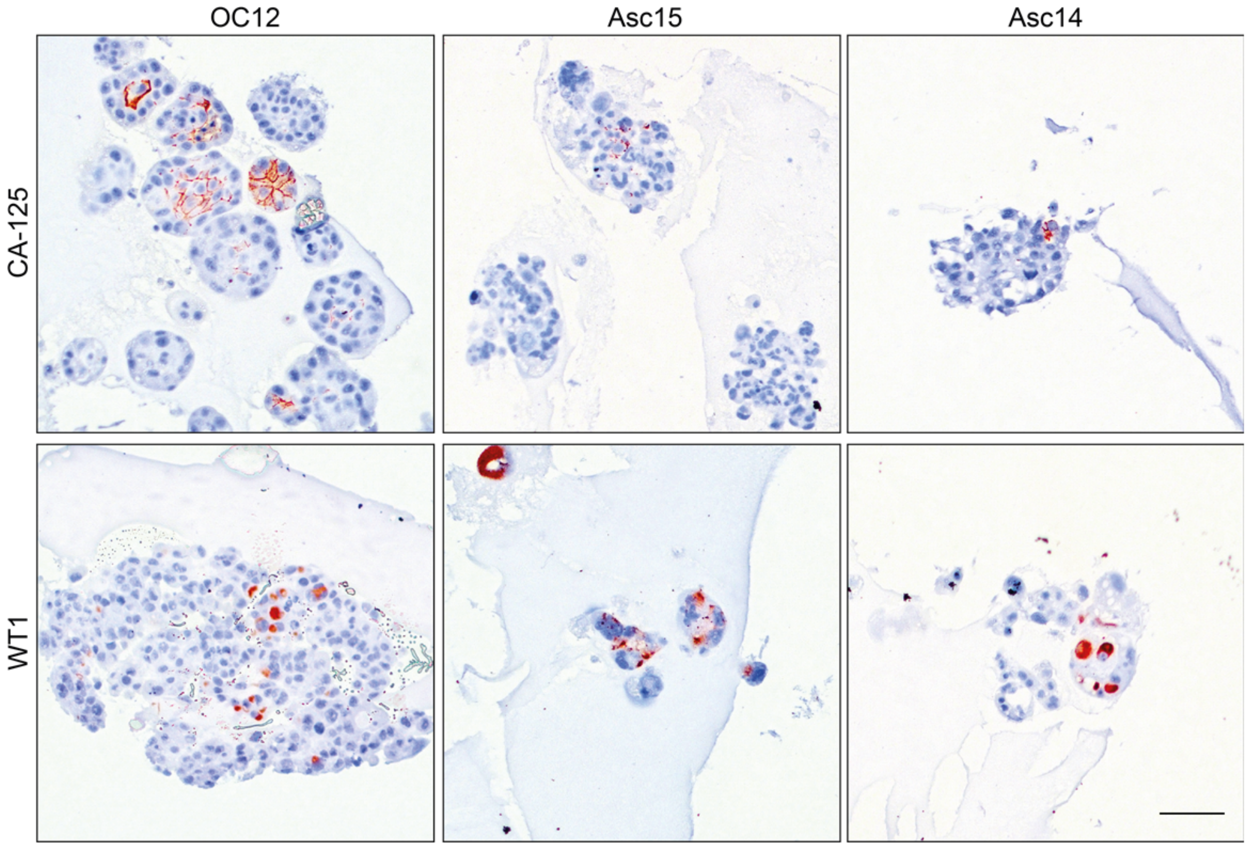

Appendix Figure S2 - Cancer organoids from patient derived OC cells retain tumour markers CA-125 and WT1.

Immunohistochemistry for CA-125 and WT1 was performed with OC12, Asc15 and Asc14 cells grown for 8 days on Matrigel. Scale bar is 200 µm.

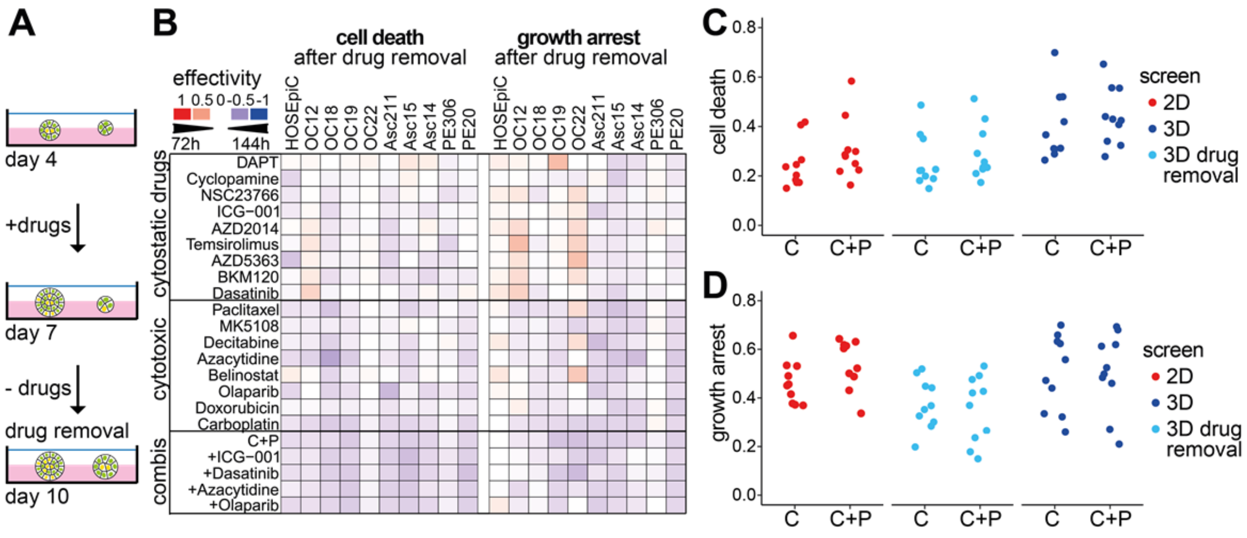

Appendix Figure S3 - Drug-induced growth arrest and cell death increase in ovarian cancer organoids with time.

A. Schematic overview of drug testing in organoid culture with the DeathPro assay.

B. Differences of drug effects in *DeathPro* screens in OC cells over time. Cell death and growth arrest were determined after 72 h or 144 h and subtracted from each other. Blue heat map color indicates stronger effect after 144 h, red color stronger effect after 72 h.

C. Cell death (AUCd) induced by Carboplatin (C) or Carboplatin and Paclitaxel (C+P) in OC cells and HOSEpiC grown in 2D or 3D culture.

D. Growth arrest (AUCpi) induced by Carboplatin (C) or Carboplatin and Paclitaxel (C+P) in OC cells and HOSEpiC.

Two-sided Welch's *t*-test was performed in C and D.

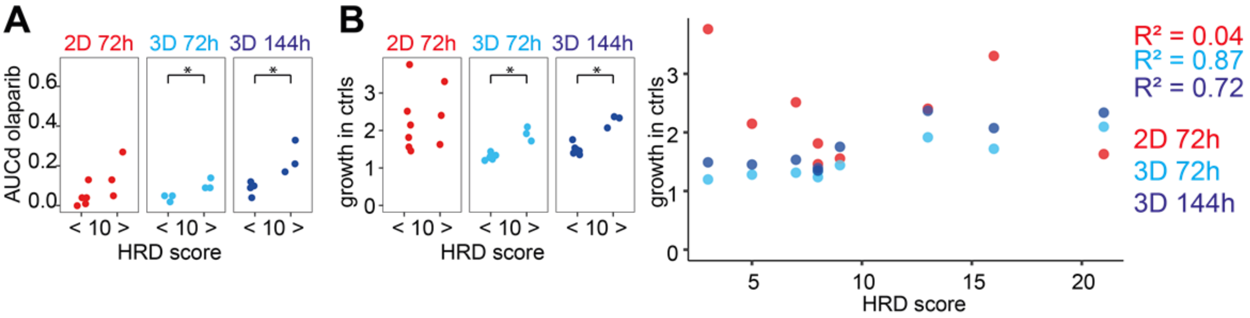

Appendix Figure S4 - Homologous recombination deficiency scores and cell growth.

A. Drug-induced cell death (AUCd) of all nine primary OC cell lines divided into two groups with low (<10) or high (>=10) homologous repair deficiency (HRD) score. Cytotoxicity induced by olaparib (A) is higher in HR deficient cells.

B. Growth in untreated patient-derived OC cells correlates with HRD score in 3D culture but not in 2D culture.

*=p<0.05 in two-sided Welch's *t*-test.
