# Supplementary material for: Screening drug effects in patient‐derived cancer cells links organoid responses to genome alterations
Source: Mol Syst Biol. 2017 Nov 27;13(11):955. doi: 10.15252/msb.20177697 (PMC5731348; doi:10.15252/msb.20177697)
Supplement: Supplementary file 2 — Expanded View Figures PDF [file MSB-13-955-s002.pdf]

Expanded View Figures

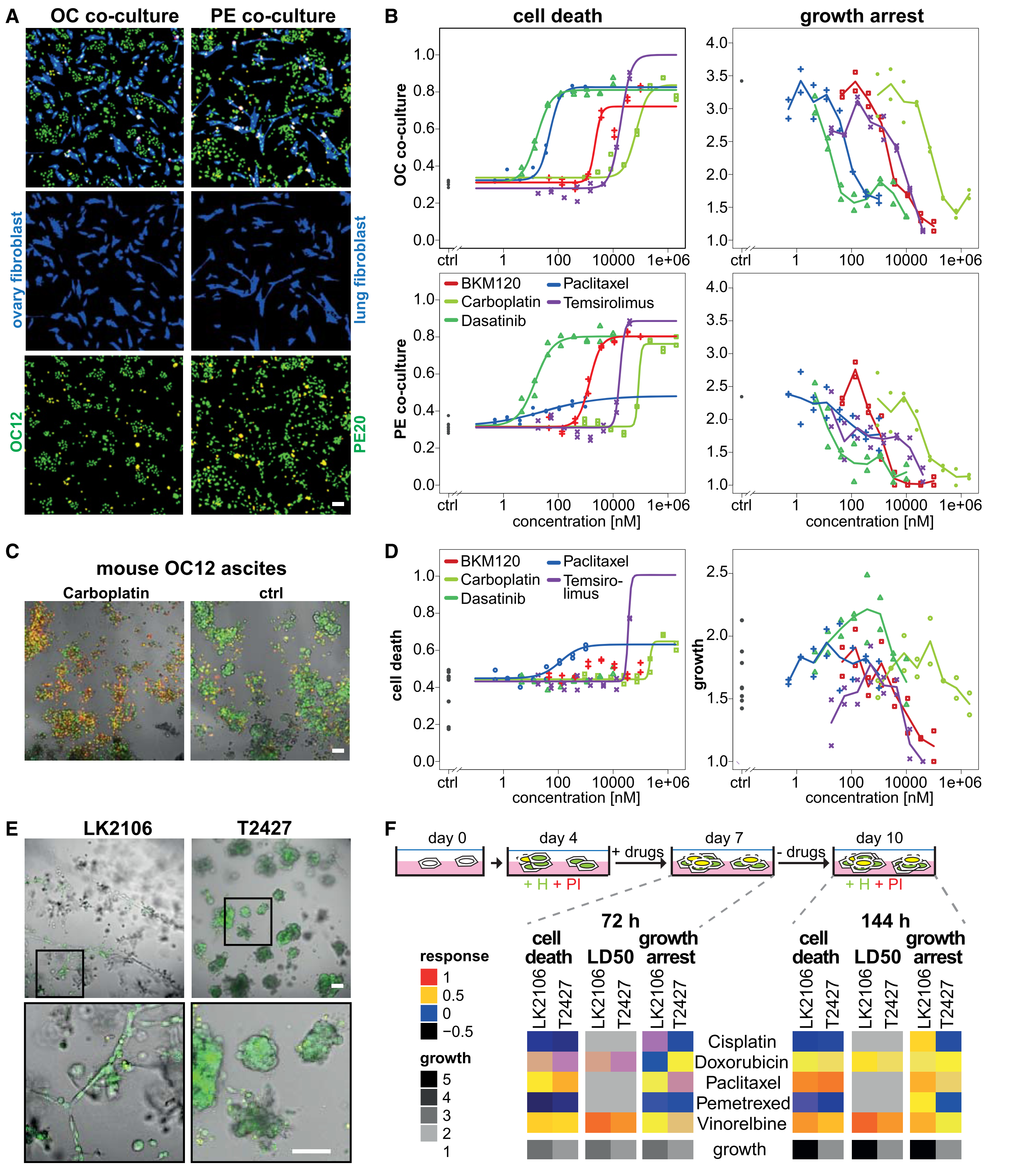

Figure EV1.

Figure EV1. ***DeathPro*** **assay resolves drug responses in ovarian cancer co-cultures, xenograft-derived cells and lung cancer organoids.**

A Representative images of primary ovarian cancer (OC12) or pleural effusion-derived cells (PE20) seeded together with human ovary (HOF) or lung fibroblasts (IMR90, both blue channel) to model cellular interactions in the primary tumour or lung metastasis. The *DeathPro* assay solely analyses drug response of Hoechst and PI-stained ovarian cancer nuclei (green and red channel) and excludes live fibroblasts (CellTracker Green stained, blue channel).

B *DeathPro* resolves cell death and growth arrest in co-culture pilot drug screens. Cells were treated for 72 h with indicated drugs.

C Drug test with OC patient-derived xenograft model. Ascites from mice intraperitoneally injected with OC12 was harvested, seeded onto Matrigel and used for drug screening from day 1–4 postseeding. Representative images depict untreated (ctrl) or 2 mM carboplatin-treated cells stained with Hoechst (green) and PI (red) at day 4.

D *DeathPro* resolves cytotoxic and cytostatic effects in mouse OC12 ascites cells. A pilot drug screen similar to the co-cultures screens (A, B) was performed as described in (C). Growth of ascites cells is heterogeneous in controls and drug-treated conditions.

E Lung cancer cells lines derived from lymph node (LN2106) or lung tumour (T2427) were cultured in 3D on Matrigel for 7 days and stained with Hoechst (H, green) and propidium iodide (PI, red).

F Pilot drug screen in 3D cultured lung cancer cells from patients. Drug responses and cell growth measured after 72 or 144 h for drugs as indicated. For better visualization, logarithmic LD50 values were normalized so that 1 and 0 correspond to minimum and maximum dose, respectively.

Data information: Scale bar is 100 μm.

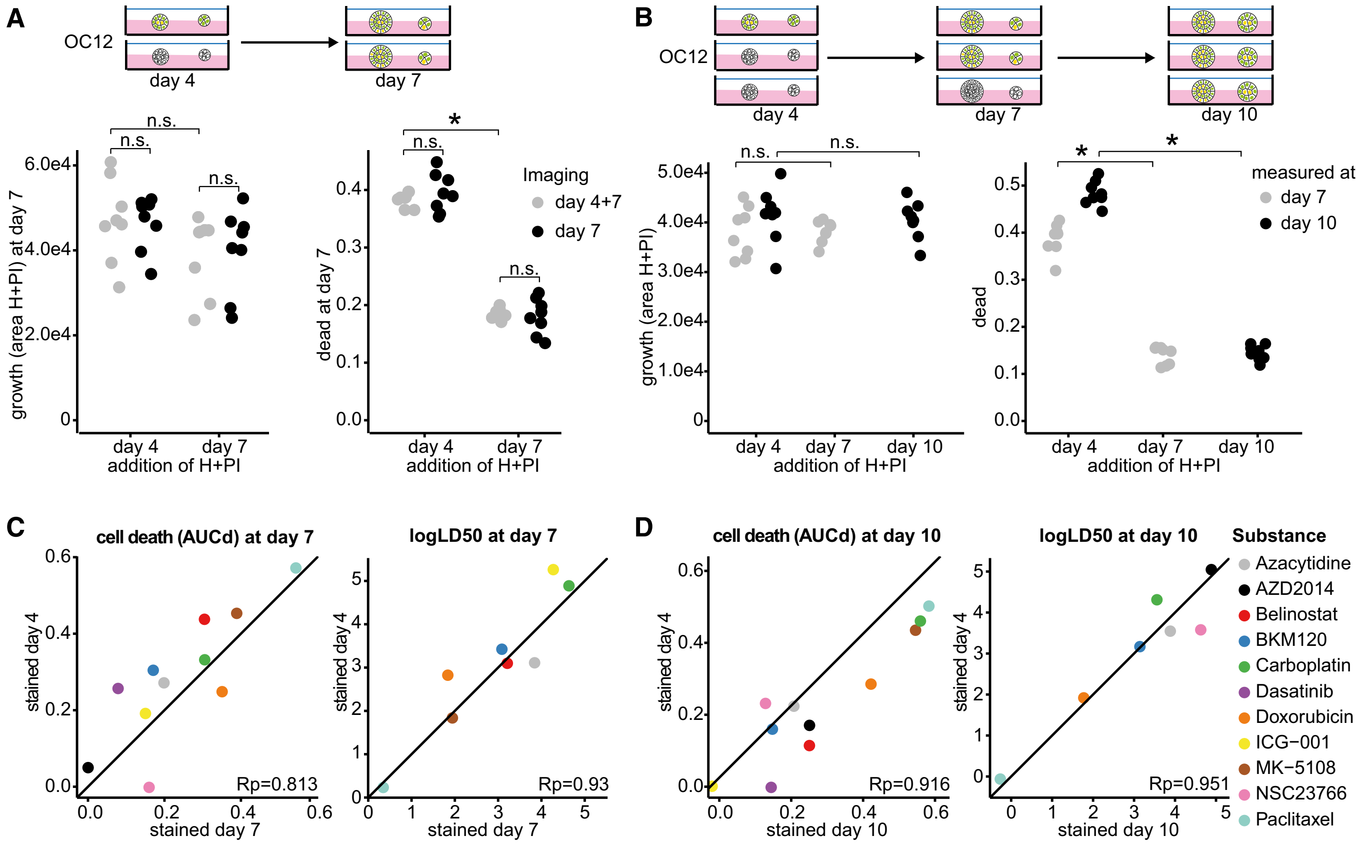

Figure EV2. ***DeathPro*** **dyes Hoechst and PI do not affect organoid growth and drug responses.**

A, B In the absence of drugs, Hoechst (H) and PI do not change growth but lead to more cell death in organoids. (A) OC12 organoids were stained at day 4 or day 7 with H and PI and imaged already at day 4 or only at day 7 to compare the influence of imaging on growth and cell death. Both were quantified at day 7. (B) Organoids were stained at day 4, 7 or 10, and growth and cell death were quantified at day 7 or 10 as indicated.

C, D Staining organoids with H and PI has no effect on cytotoxic responses to drugs. *DeathPro* assay was performed with OC12 organoids and the indicated drugs. Cell death was determined at day 7 (C) or day 10 (D) and compared between organoids stained at treatment begin (day 4) or end at day 7 (C) or day 10 after drug washout (D).

Data information: n.s., not significant, *$P < 0.05$ in two-tailed Welch's t-test. Rp, Pearson correlation coefficient. Black line ($x = y$) is depicted as reference for perfect correlation.

 © 2017 The Authors

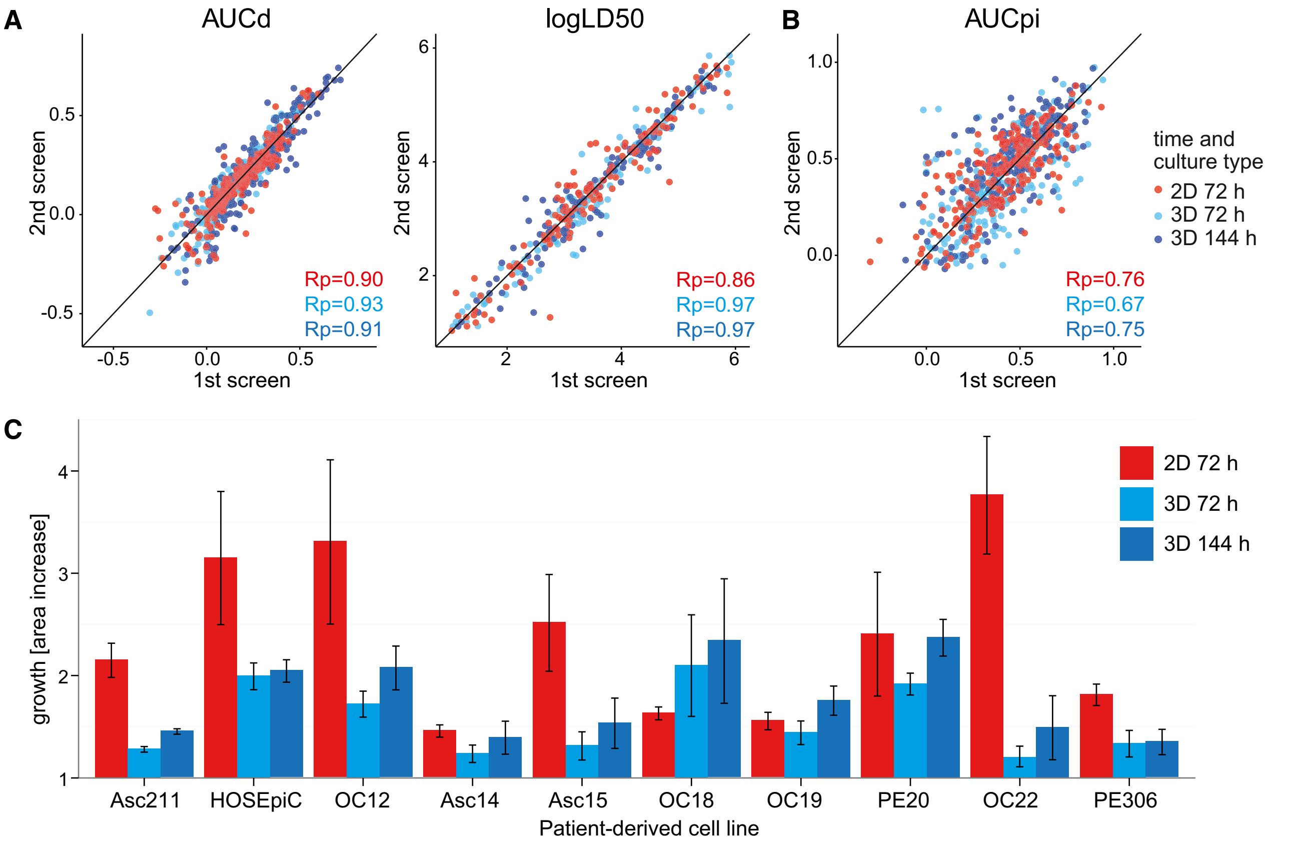

Figure EV3. Reproducibility of *DeathPro* drug screens.

A, B Scatterplots of AUCd, logLD50 and AUCpi values of biological replicates determined in 2D and 3D culture screens of patient-derived ovarian cancer cell lines and HOSEpiC (220 measurements per time point: 22 drug dilution series on 10 cell lines) with corresponding Pearson correlation coefficients (Rp). The black line ($x = y$) is depicted as reference for perfect correlation.

C Growth of patient-derived OC cell lines and HOSEpiC in organoid culture or as cell monolayers (2D). Growth within 72 or 144 h was measured from day 4 on after seeding in 3D or from day 1–4 in 2D ($n = 4$, mean ± SD shown).

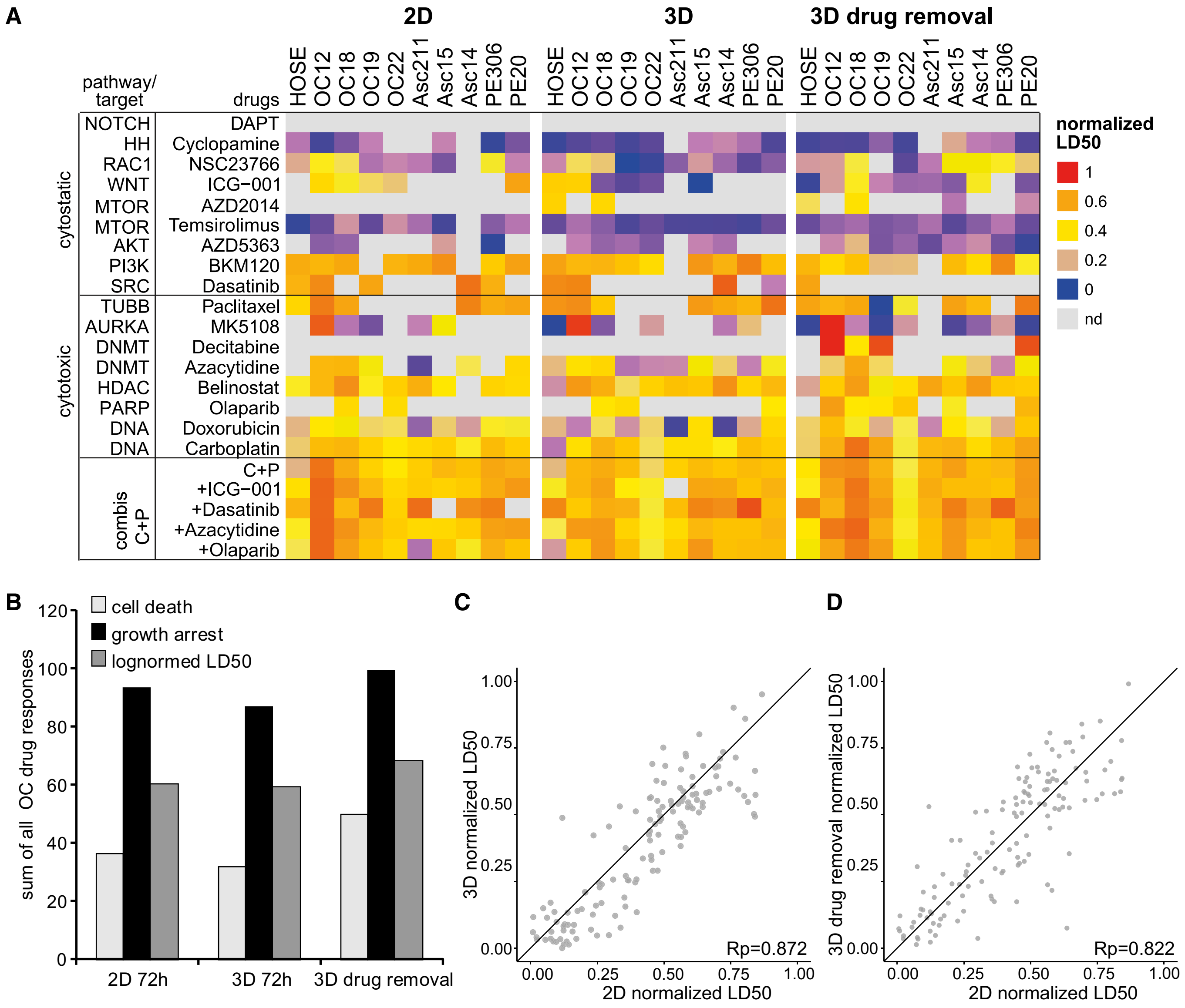

Figure EV4. Drug sensitivity described by LD50 is similar in cells cultured in 2D or as organoids.

A LD50 values determined after 72-h drug exposure, or 72-h drug removal in OC patient-derived cells cultured in 2D or as organoids. For better visualization, LD50s have been rescaled to values between 0 and 1, representing maximum and minimum dose. All values shown are means of two independent biological replicates.

B Sum of all responses across all drugs and combinations and OC cells tested in 2D culture and 3D culture.

C, D Comparison of LD50s in 2D vs. 3D after 72-h treatment (C) or after drug removal (D). The black line ($x = y$) is depicted as reference for perfect correlation.

Data information: Rp, Pearson correlation coefficient; C + P, carboplatin + paclitaxel.

 © 2017 The Authors

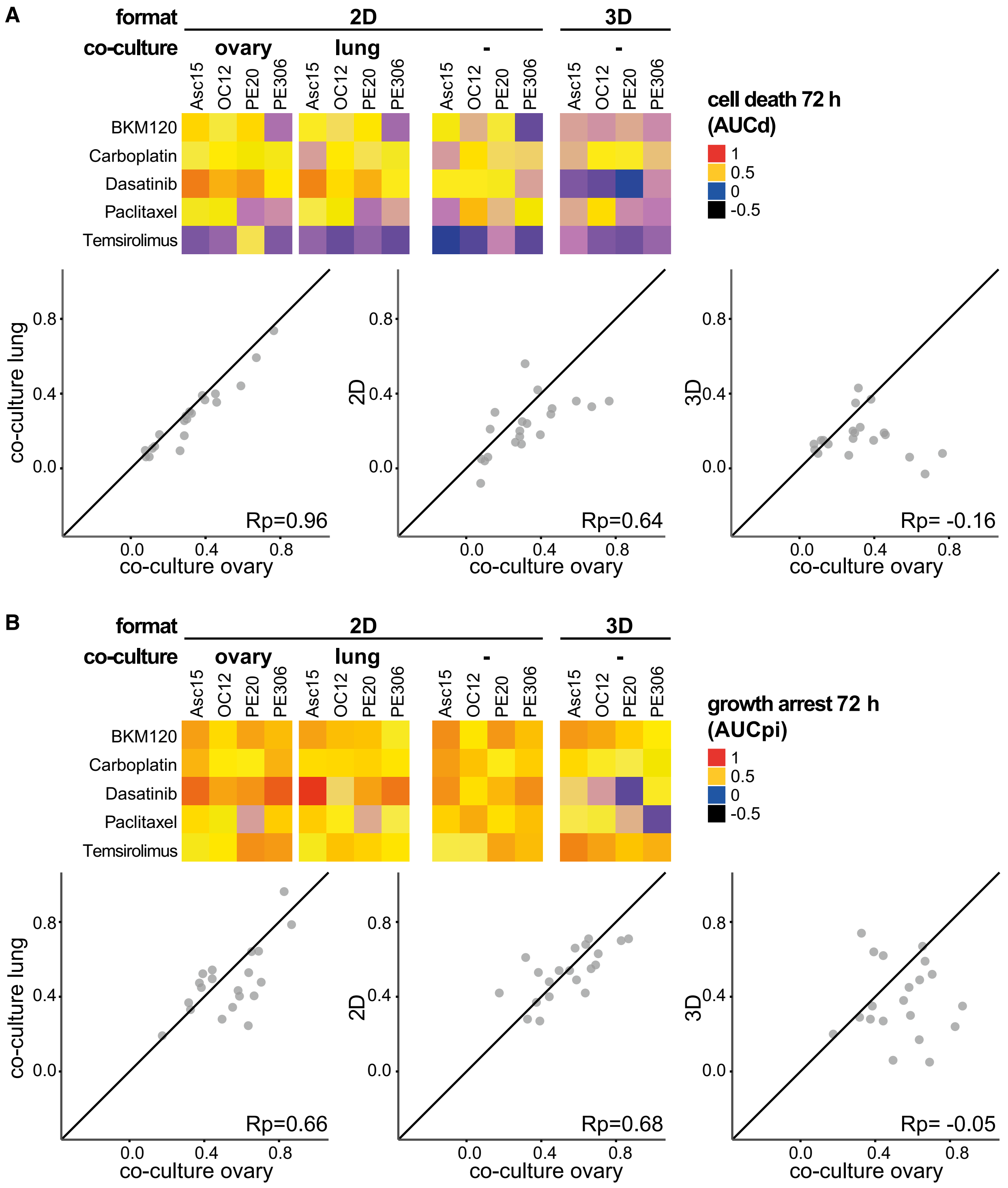

Figure EV5. Patient drug responses in 2D co-cultures resemble responses in 2D monocultures and not 3D organoid cultures.

A Drug-induced cell death is similar in co-cultures with human ovary or lung fibroblasts. Cytotoxicity observed in co-cultures correlates stronger with cytotoxicity in 2D than in 3D cultures. Heat maps depict cell death induced in four OC cell lines in 2D or 3D cultures and co-cultures.

B Drug-induced cell death correlates strongly between OC co-cultures with human ovary or lung fibroblasts. Growth arrest in co-cultures resembles drug response in 2D monocultures but not 3D cultures. Heat maps depict growth arrest induced in four OC cell lines in 2D or 3D cultures and co-cultures.

Data information: The black line ($x = y$) is depicted as reference for perfect correlation. Rp, Pearson correlation coefficient.

© 2017 The Authors
