# Supplementary material for: Screening drug effects in patient‐derived cancer cells links organoid responses to genome alterations
Source: Mol Syst Biol. 2017 Nov 27;13(11):955. doi: 10.15252/msb.20177697 (PMC5731348; doi:10.15252/msb.20177697)
Supplement: Supplementary file 3 — Table EV1 [file MSB-13-955-s003.docx]

**Table EV1: Classification of OC patient samples used for serous OC cell lines.**

| **PDCL** | **origin** | **FIGO** | **grade** | **TNM** | **Pathological disease** | **treatment** |
| --- | --- | --- | --- | --- | --- | --- |
| OC12 | tumour | IIIc | G3 | TN1M1 | serous adenocarcinoma | not treated |
| OC18 | tumour | IIIc | G3 | TN1M1 | serous adenocarcinoma | not treated |
| OC19 | tumour | IIIc | G3 | TN1M1 | serous adenocarcinoma | not treated |
| OC22 | tumour | IIIc | nd | nd | serous adenocarcinoma | not treated |
| Asc211 | ascites | IIIC | G3 | pT3c pN1 | serous adenocarcinoma | 1st line, 2nd line, 3rd line |
| Asc14 | ascites | IV | G3 | TN1M1 | serous adenocarcinoma | 1st, 2nd, 3rd line |
| Asc15 | ascites | IIIc | G3 | TN1M1 | serous adenocarcinoma | 1st, 2nd line |
| PE306 | pleural effusion | IV | G2 | T3 | serous adenocarcinoma | 1st line |
| PE20 | pleural effusion | IIIc | G3 | TN1M1 | serous adenocarcinoma | 1st, 2nd line: Morab study |
