# Supplementary material for: Screening drug effects in patient‐derived cancer cells links organoid responses to genome alterations
Source: Mol Syst Biol. 2017 Nov 27;13(11):955. doi: 10.15252/msb.20177697 (PMC5731348; doi:10.15252/msb.20177697)
Supplement: Supplementary file 4 — Table EV2 [file MSB-13-955-s004.docx]

**Table EV2: Inhibitors used for the *DeathPro* screens**

| **Inhibitors** | **Status in OC therapy** | **producer** | **Lot #** | **stock conc. [mM]** | **starting conc. [µM]** | **solvent** |
| --- | --- | --- | --- | --- | --- | --- |
| paclitaxel | 1^st^ line | Selleckchem | 9 | 10 | 1 | DMSO |
| carboplatin | 1^st^ line | Cayman Chemical | 0453486-12 | 20 | 2,000 | water |
| doxorubicin | 2^nd^ line | StressMarq Biosciences | 150120 | 1 | 10 | PBS |
| olaparib | 4^th^ line | Cayman Chemical | 0461572-5 | 10 | 100 | DMSO |
| BKM120 | phase I | Selleckchem | S224704 | 50 | 100 | DMSO |
| MK-5108 | phase I | Selleckchem | S277001 | 10 | 100 | DMSO |
| belinostat | phase II | BioVision | 9C242480 | 50 | 100 | DMSO |
| AZD2014 | phase II | Selleckchem | 1 | 50 | 5 | DMSO |
| AZD5363 | phase II | Selleckchem | S801901 | 50 | 500 | DMSO |
| temsirolimus | phase II | Sigma | 110M4716V | 10 | 40 | DMSO |
| azacytidine | phase II | Sigma | MKBR7212 | 50 | 100 | DMSO |
| decitabine | phase II | Sigma | MKBR6437V | 10 | 500 | water |
| dasatinib | phase II | LC Laboratories | BDS109 | 100 | 10 | DMSO |
| cyclopamine | preclinical | Adipogen | A00152 | 4.5 | 45 | ethanol |
| DAPT | preclinical | Sigma | 014M4609V | 25 | 100 | DMSO |
| NSC23766 | preclinical | Sigma | 044M4761V | 10 | 1,000 | water |
| ICG-001 | preclinical | Sellekchem | 2 | 50 | 100 | DMSO |
