# Supplementary material for: Screening drug effects in patient‐derived cancer cells links organoid responses to genome alterations
Source: Mol Syst Biol. 2017 Nov 27;13(11):955. doi: 10.15252/msb.20177697 (PMC5731348; doi:10.15252/msb.20177697)
Supplement: Supplementary file 5 — Code EV1 [file MSB-13-955-s005.zip › DeathPro/DeathPro_Manual.pdf]

DeathPro Manual

1. Introduction

This is a basic version of the DeathPro KNIME workflow designed to analyze images derived from a 96-well drug testing plate imaged at several time points. Cell death parameters (AUCd, LD50) are calculated for each time point and growth arrest (AUCpi) to a reference time point. The workflow has been simplified to illustrate the basic concepts of image processing and parameter calculation presented in the paper.
We tested "DeathPro" on Windows 7 and 10 as well as Mac OSX. However, we recommend using Windows since it requires less manual installation steps and the R integration in KNIME runs more robust.

→ in case you already have KNIME installed: please check if you have all required extensions (STEP 5a)!

2. Installation of KNIME and required extensions

STEP 1: Download KNIME

https://www.KNIME.org/downloads/overview --> second Tab: Download KNIME:

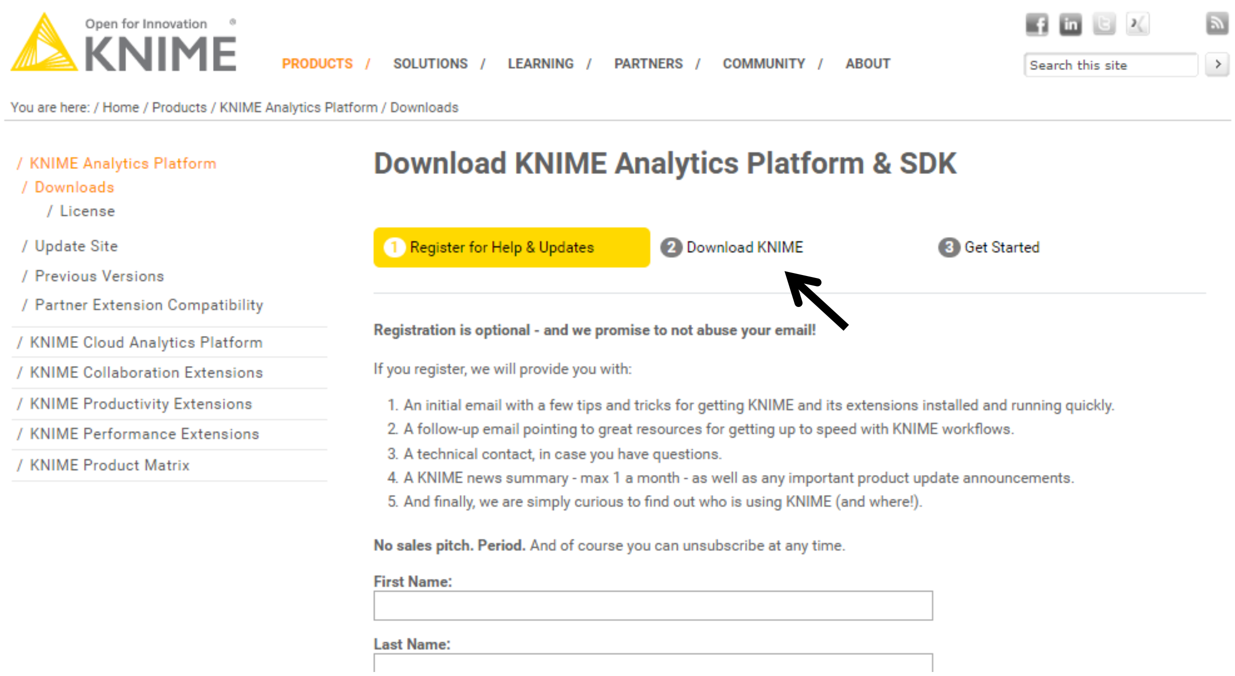

Select a suitable version for your operating system. You can install the smaller, basic version (~300 MB) without extensions:

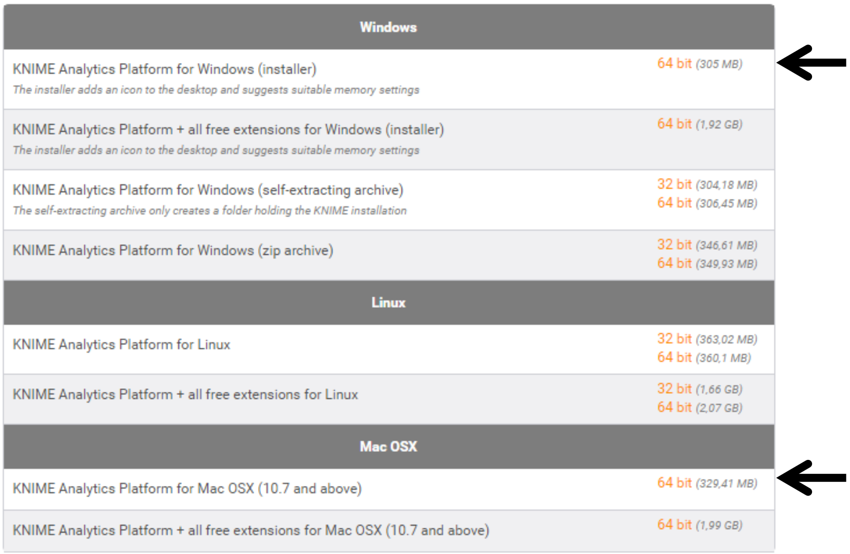

Windows	
KNIME Analytics Platform for Windows (installer)<br>*The installer adds an icon to the desktop and suggests suitable memory settings*	64 bit (305 MB)
KNIME Analytics Platform + all free extensions for Windows (installer)<br>*The installer adds an icon to the desktop and suggests suitable memory settings*	64 bit (1,92 GB)
KNIME Analytics Platform for Windows (self-extracting archive)<br>*The self-extracting archive only creates a folder holding the KNIME installation*	32 bit (304,18 MB)<br>64 bit (306,45 MB)
KNIME Analytics Platform for Windows (zip archive)	32 bit (346,61 MB)<br>64 bit (349,93 MB)
**Linux**	
KNIME Analytics Platform for Linux	32 bit (363,02 MB)<br>64 bit (360,1 MB)
KNIME Analytics Platform + all free extensions for Linux	32 bit (1,66 GB)<br>64 bit (2,07 GB)
**Mac OSX**	
KNIME Analytics Platform for Mac OSX (10.7 and above)	64 bit (329,41 MB)
KNIME Analytics Platform + all free extensions for Mac OSX (10.7 and above)	64 bit (1,99 GB)

STEP 2: install KNIME, memory settings: >2000 MB to run smoothly, >4000 MB recommended

STEP 3: Open KNIME to install extensions
In case workspace could not be launched, select another folder

STEP4: Help > Install New Software > Available Software Sites > check: Stable Community Contributions

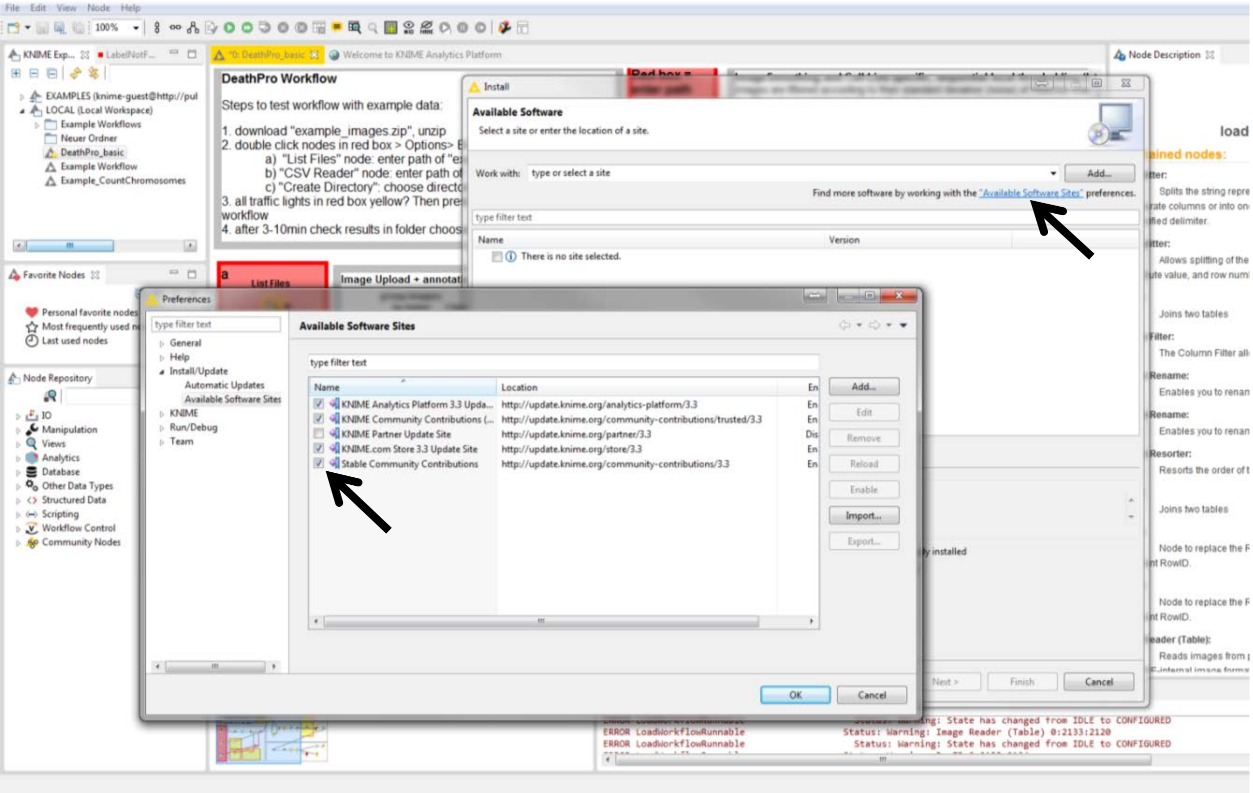

Click Ok, close “Install” window

STEP 5a: Open KNIME: File >Install KNIME Extensions

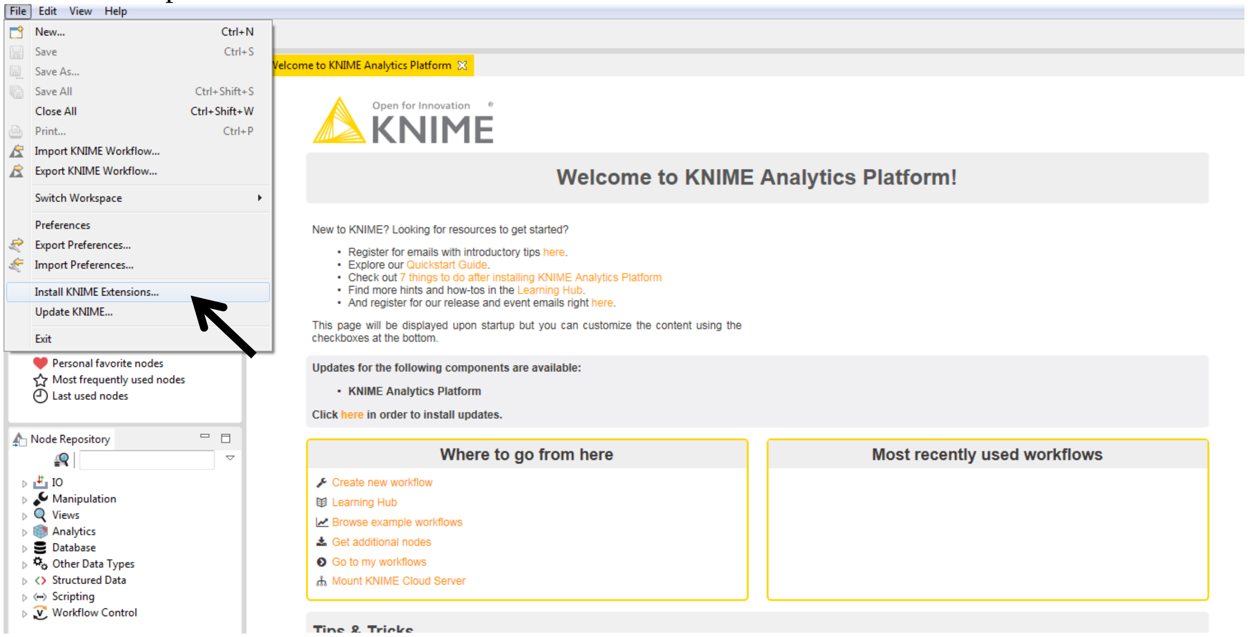

For a minimal installation of extensions required for DeathPro select:
KNIME & Extensions:

- KNIME File Handling Nodes
- KNIME Interactive R Statistics Integration
- KNIME R Statistics Integration (Windows Binaries) (only needed for Windows)

KNIME Community Contributions- Image Processing and Analysis:

- KNIME Image Processing
- KNIME Image Processing- ImageJ Integration (Beta)

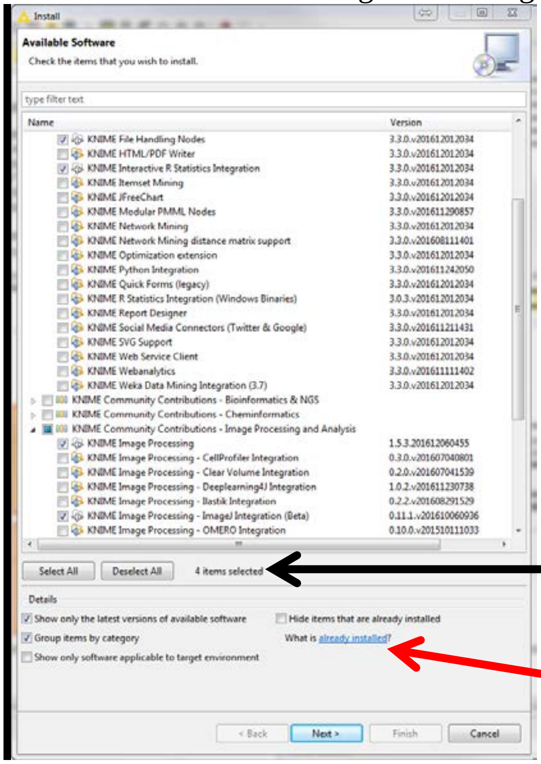

After you selected all 4 items click “Next”

If you are already using KNIME: please check here if you have all required extensions

You will see the selected extensions to be installed:

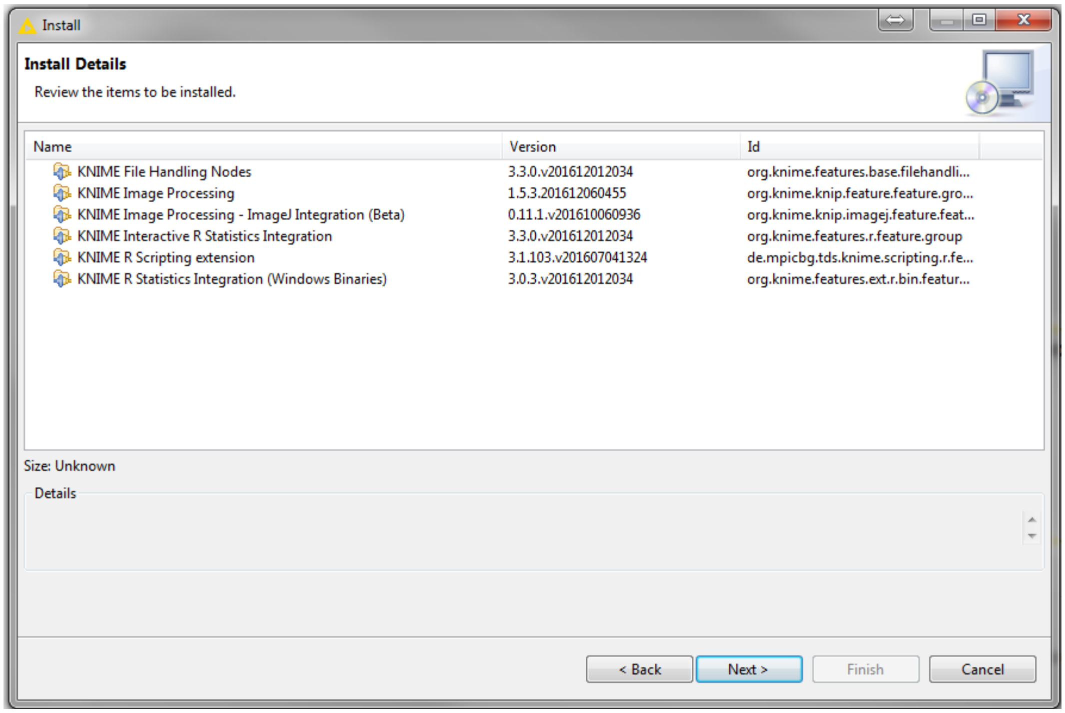

Finish the installation. *“Unsigned Content” warning during the installation is OK, you can safely ignore it.*

Alternative to minimal installation: Install the complete extension packages including

- KNIME & Extensions
- KNIME Community Contributions- Image Processing and Analysis

BUT: This requires more time and is not necessary to run DeathPro!

STEP 5b only required for Mac OS X users: install R + R packages needed

- Install R-3.3.2 from https://cran.r-project.org/bin/macosx/ (Mac OS X - release 10.6 and above)
- In File>Preferences>KNIME>R, set the path to R Home: /Library/Frameworks/R.framework/Versions/Current/Resources
- In the R.app running, install the packages Rserve, Cairo and XQuartz:
    - install.packages('Rserve')
    - install.packages('Cairo')
    - install.packages(' XQuartz ')
- to run DeathPro install the following packages:
    - install.packages('stringr')
    - install.packages('drc')
    - install.packages('plotrix')
    - install.packages('plyr')

STEP 6: Restart KNIME, you will see the installed extensions “R” and “Image Processing”

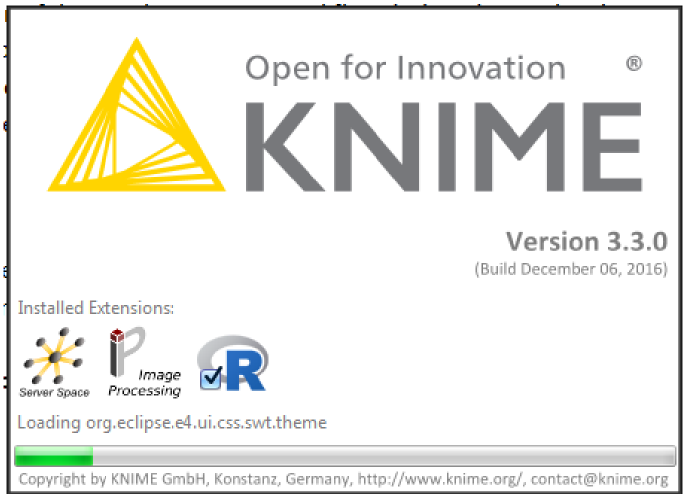

3. Running DeathPro

STEP 1: Import DeathPro workflow
File > Import workflow > browse + select “DeathPro.knwf” > Finish
See workflow appearing in KNIME Explorer window

STEP 2: Open “DeathPro” workflow by double clicking it in KNIME Explorer window
Follow the instructions in the workflow to run the analysis of the example images and to see the results. If all traffic lights under the nodes are green, the workflow has been fully executed and all data has been processed and analyzed. Check if you obtained **three pdf-files** and **six csv-files** as in the “example_results” folder.

How to deal with Nodes (little colored boxes connected by arrows):
Double click: open node + configure it
Right klick: opens the node's context menu

Please refer to the “KNIME Quickstart Guide” (https://tech.KNIME.org/files/KNIME_quickstart.pdf) for an introduction into how to use KNIME and to “The KNIME Image Processing Extension User Manual” (http://tech.KNIME.org/files/KNIMEip/KNIME-ip_user_manual_DRAFT.pdf) for information on image processing with KNIME.

In case “Memory is low.” warning appears and no progress is made over 10 min: close KNIME, check the knime.ini file and change “-Xmx____m” to “-Xmx8040m” if you have 12GB or 16GB RAM or to 50-65% of your available RAM.

4. Further Notes

For Mac OS X users

In case problems arise with the R Snippet Node, e.g. "Home invalid" message check path to R Home: in File>Preferences>KNIME>R, set the path to R Home:
/Library/Frameworks/R.framework/Versions/Current/Resources *or*
/Library/Frameworks/R.framework/Resources

For users that analyze other data than the example data

The images should be acquired similar as outlines in the paper and have similar properties as the example data. Otherwise analysis parameters have to be adopted for images with different resolution or bit depth than the example images. Signal intensities should be as described in the Methods section: weak Hoechst intensities of live cells need to be above the background and PI intensities should be close to the dynamic range (no saturation).

The naming of image files or folders should follow the example data structure. Otherwise the extraction of time point and cell line names might not work. In the plate layout concentration units have to be in "nM", "µM", or "mM". Other entries are not evaluated. Controls have to be denoted as "ctrl" for untreated or "DMSO" or "EtOH" for DMSO or ethanol controls. Controls denoted in another way will be recognized as drugs and not used as baseline for drug response curve fits or AUC calculations. Calculation and plotting of dead values is implemented for the situation that all substances are tested in the same number of dilutions. Using e.g. six concentrations for one drug and eight for another drug probably yields errors.
