# Supplementary figures and images for: Screening drug effects in patient‐derived cancer cells links organoid responses to genome alterations (part 2 of 2)
Source: Mol Syst Biol. 2017 Nov 27;13(11):955. doi: 10.15252/msb.20177697 (PMC5731348; doi:10.15252/msb.20177697)

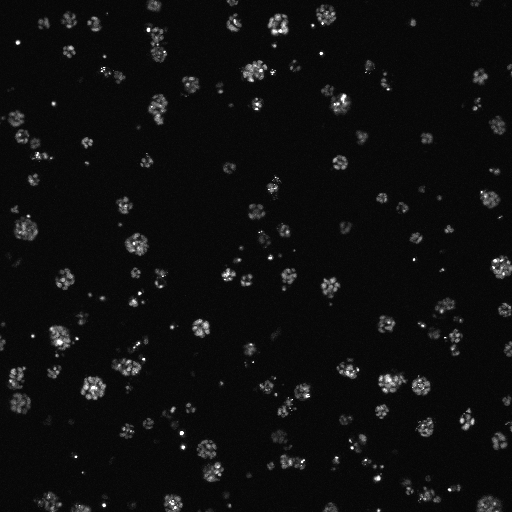

Supplement: Supplementary file 5 — Code EV1 [file MSB-13-955-s005.zip › DeathPro/example_images/150612_OC12_0h_2.1_MIPs/OC12_0h__W0026__P0001_channel0.tif]

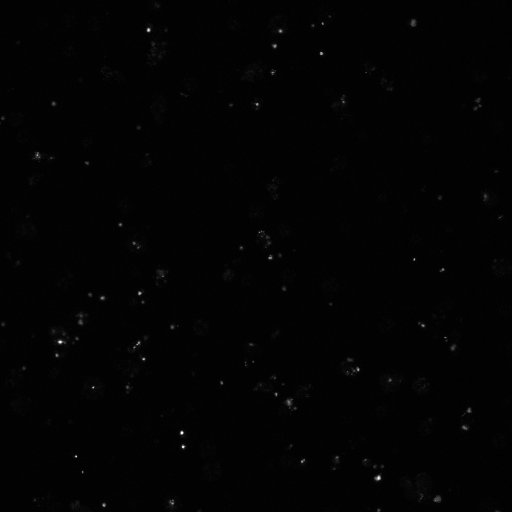

Supplement: Supplementary file 5 — Code EV1 [file MSB-13-955-s005.zip › DeathPro/example_images/150612_OC12_0h_2.1_MIPs/OC12_0h__W0026__P0001_channel1.tif]

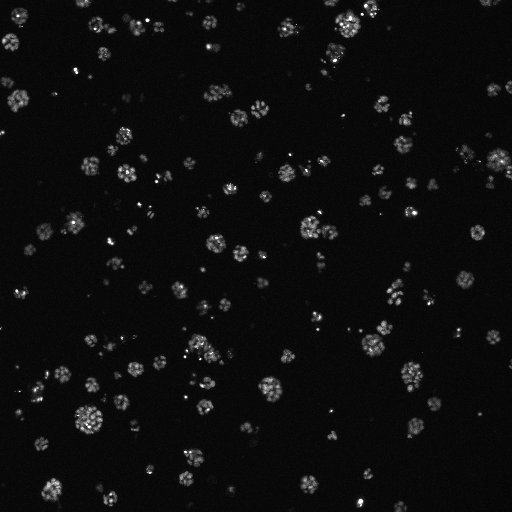

Supplement: Supplementary file 5 — Code EV1 [file MSB-13-955-s005.zip › DeathPro/example_images/150612_OC12_0h_2.1_MIPs/OC12_0h__W0026__P0002_channel0.tif]

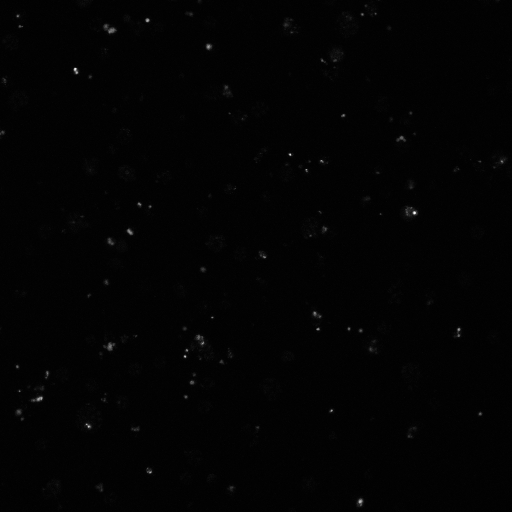

Supplement: Supplementary file 5 — Code EV1 [file MSB-13-955-s005.zip › DeathPro/example_images/150612_OC12_0h_2.1_MIPs/OC12_0h__W0026__P0002_channel1.tif]

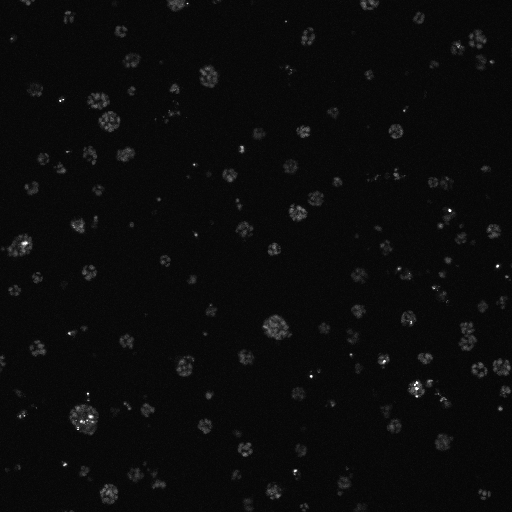

Supplement: Supplementary file 5 — Code EV1 [file MSB-13-955-s005.zip › DeathPro/example_images/150612_OC12_0h_2.1_MIPs/OC12_0h__W0027__P0001_channel0.tif]

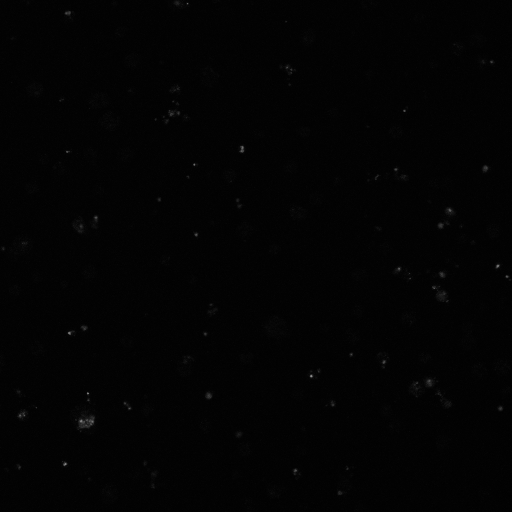

Supplement: Supplementary file 5 — Code EV1 [file MSB-13-955-s005.zip › DeathPro/example_images/150612_OC12_0h_2.1_MIPs/OC12_0h__W0027__P0001_channel1.tif]

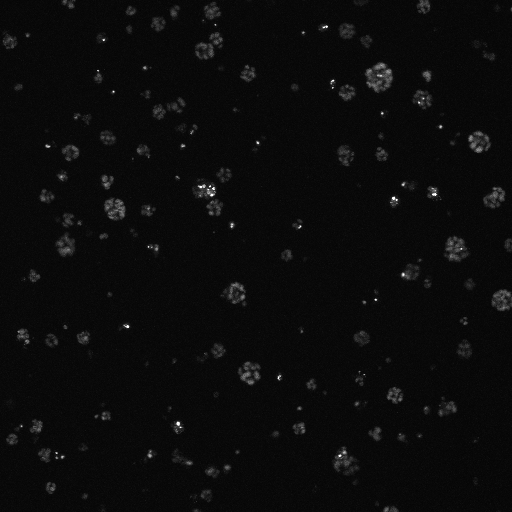

Supplement: Supplementary file 5 — Code EV1 [file MSB-13-955-s005.zip › DeathPro/example_images/150612_OC12_0h_2.1_MIPs/OC12_0h__W0027__P0002_channel0.tif]

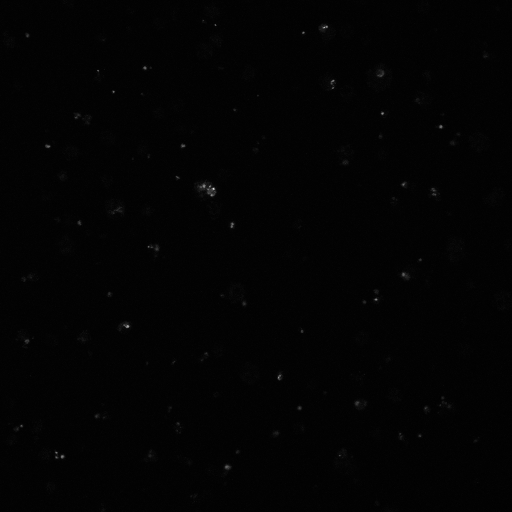

Supplement: Supplementary file 5 — Code EV1 [file MSB-13-955-s005.zip › DeathPro/example_images/150612_OC12_0h_2.1_MIPs/OC12_0h__W0027__P0002_channel1.tif]

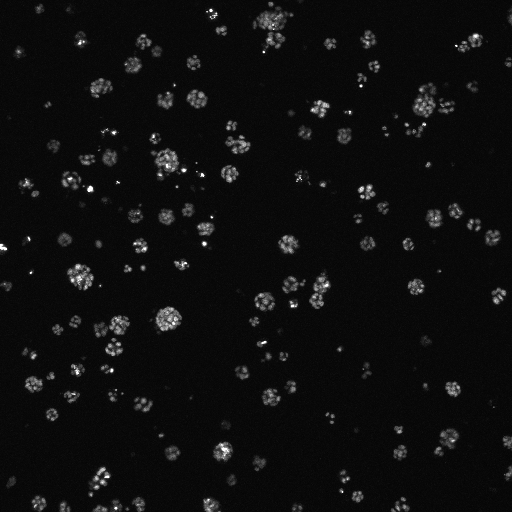

Supplement: Supplementary file 5 — Code EV1 [file MSB-13-955-s005.zip › DeathPro/example_images/150612_OC12_0h_2.1_MIPs/OC12_0h__W0028__P0001_channel0.tif]

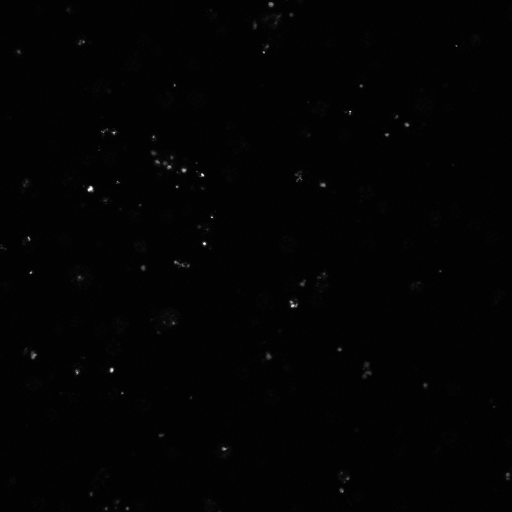

Supplement: Supplementary file 5 — Code EV1 [file MSB-13-955-s005.zip › DeathPro/example_images/150612_OC12_0h_2.1_MIPs/OC12_0h__W0028__P0001_channel1.tif]

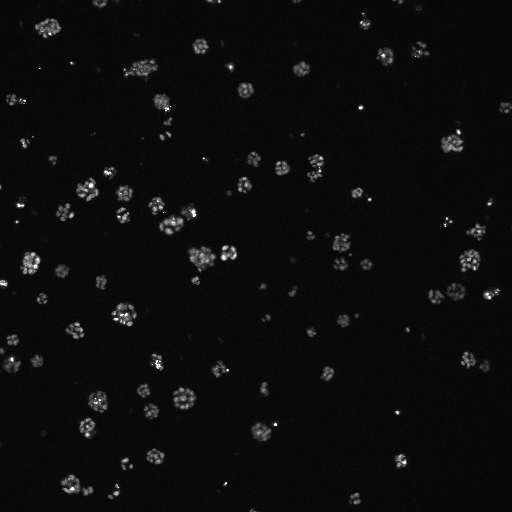

Supplement: Supplementary file 5 — Code EV1 [file MSB-13-955-s005.zip › DeathPro/example_images/150612_OC12_0h_2.1_MIPs/OC12_0h__W0028__P0002_channel0.tif]

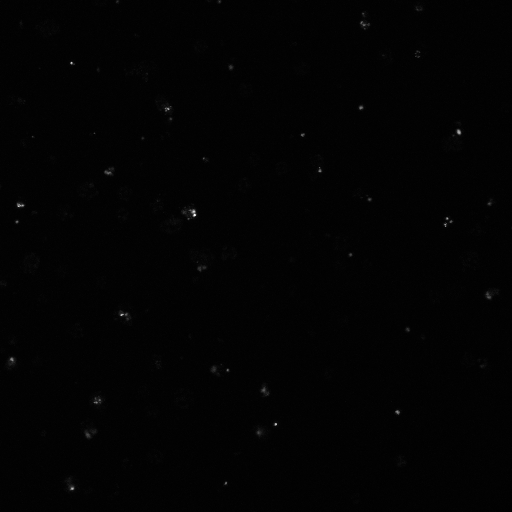

Supplement: Supplementary file 5 — Code EV1 [file MSB-13-955-s005.zip › DeathPro/example_images/150612_OC12_0h_2.1_MIPs/OC12_0h__W0028__P0002_channel1.tif]

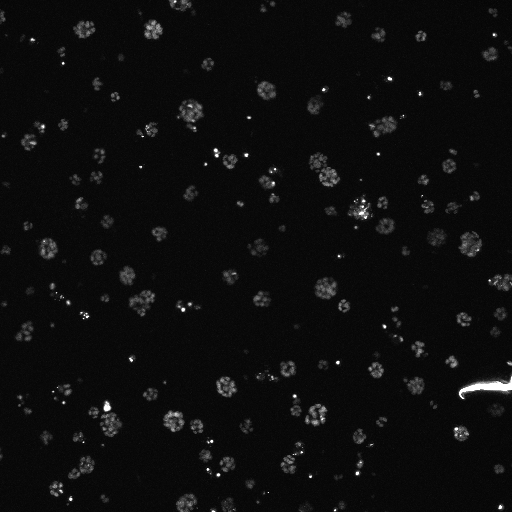

Supplement: Supplementary file 5 — Code EV1 [file MSB-13-955-s005.zip › DeathPro/example_images/150612_OC12_0h_2.1_MIPs/OC12_0h__W0029__P0001_channel0.tif]

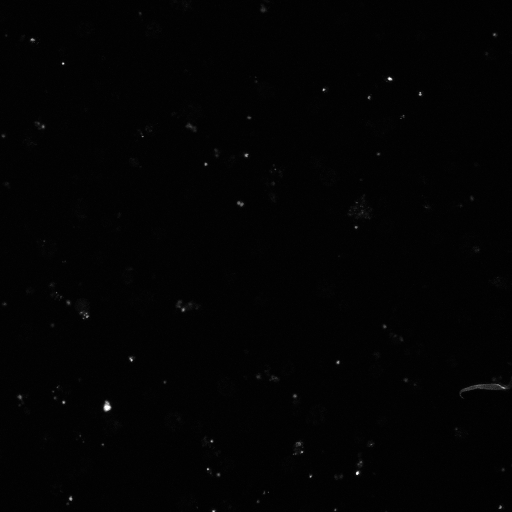

Supplement: Supplementary file 5 — Code EV1 [file MSB-13-955-s005.zip › DeathPro/example_images/150612_OC12_0h_2.1_MIPs/OC12_0h__W0029__P0001_channel1.tif]

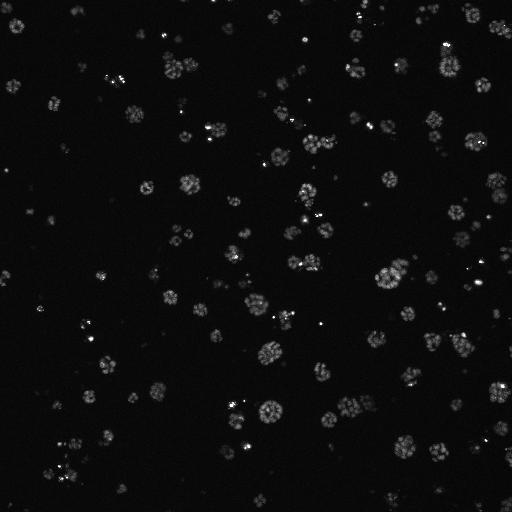

Supplement: Supplementary file 5 — Code EV1 [file MSB-13-955-s005.zip › DeathPro/example_images/150612_OC12_0h_2.1_MIPs/OC12_0h__W0029__P0002_channel0.tif]

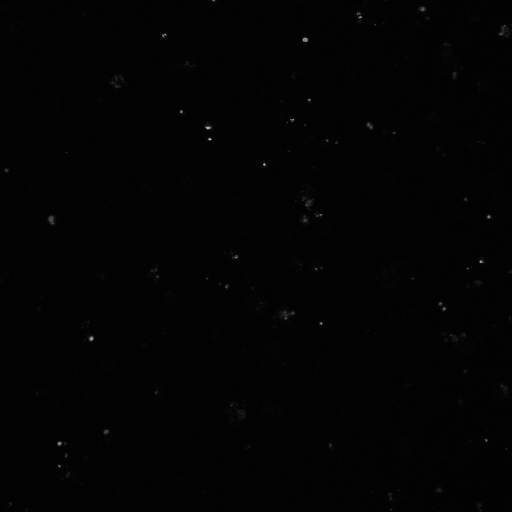

Supplement: Supplementary file 5 — Code EV1 [file MSB-13-955-s005.zip › DeathPro/example_images/150612_OC12_0h_2.1_MIPs/OC12_0h__W0029__P0002_channel1.tif]

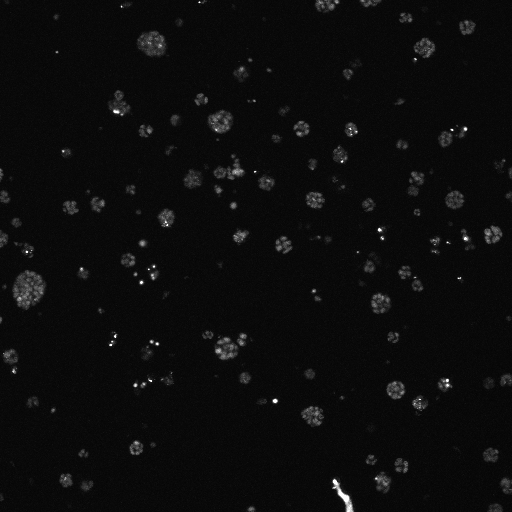

Supplement: Supplementary file 5 — Code EV1 [file MSB-13-955-s005.zip › DeathPro/example_images/150612_OC12_0h_2.1_MIPs/OC12_0h__W0030__P0001_channel0.tif]

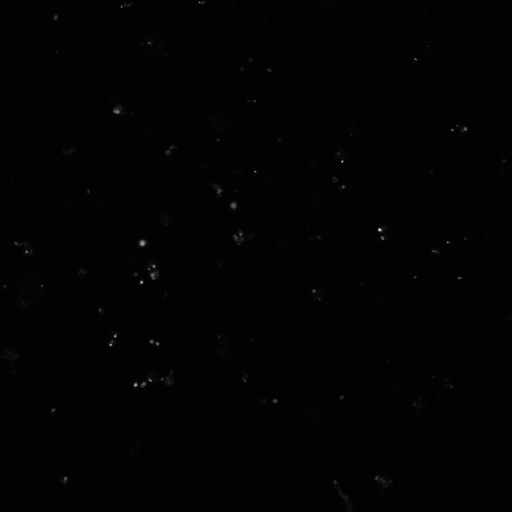

Supplement: Supplementary file 5 — Code EV1 [file MSB-13-955-s005.zip › DeathPro/example_images/150612_OC12_0h_2.1_MIPs/OC12_0h__W0030__P0001_channel1.tif]

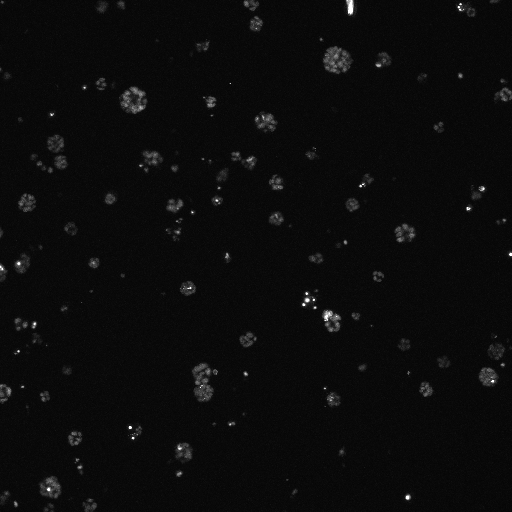

Supplement: Supplementary file 5 — Code EV1 [file MSB-13-955-s005.zip › DeathPro/example_images/150612_OC12_0h_2.1_MIPs/OC12_0h__W0030__P0002_channel0.tif]

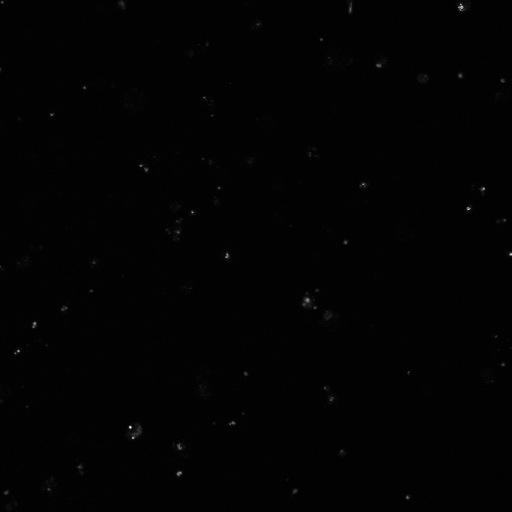

Supplement: Supplementary file 5 — Code EV1 [file MSB-13-955-s005.zip › DeathPro/example_images/150612_OC12_0h_2.1_MIPs/OC12_0h__W0030__P0002_channel1.tif]

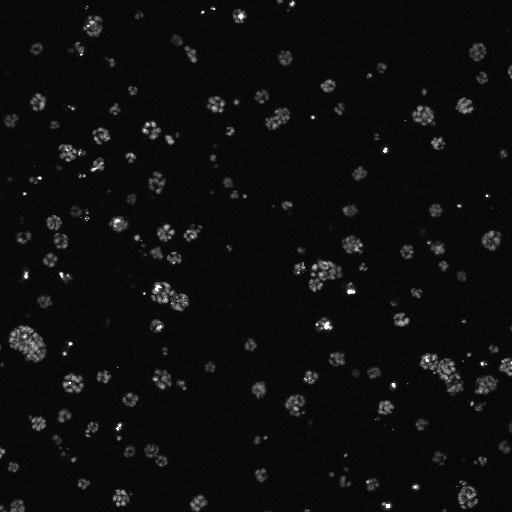

Supplement: Supplementary file 5 — Code EV1 [file MSB-13-955-s005.zip › DeathPro/example_images/150612_OC12_0h_2.1_MIPs/OC12_0h__W0031__P0001_channel0.tif]

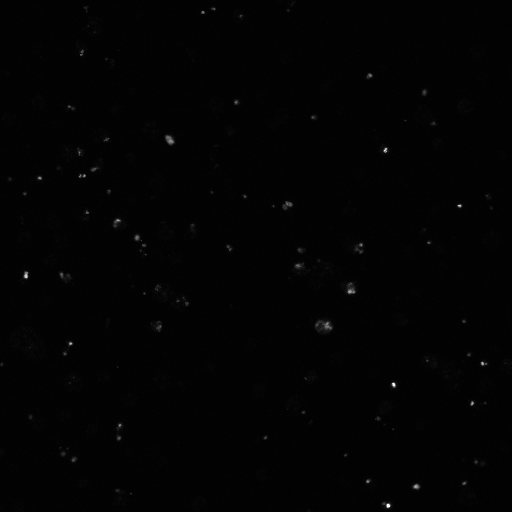

Supplement: Supplementary file 5 — Code EV1 [file MSB-13-955-s005.zip › DeathPro/example_images/150612_OC12_0h_2.1_MIPs/OC12_0h__W0031__P0001_channel1.tif]

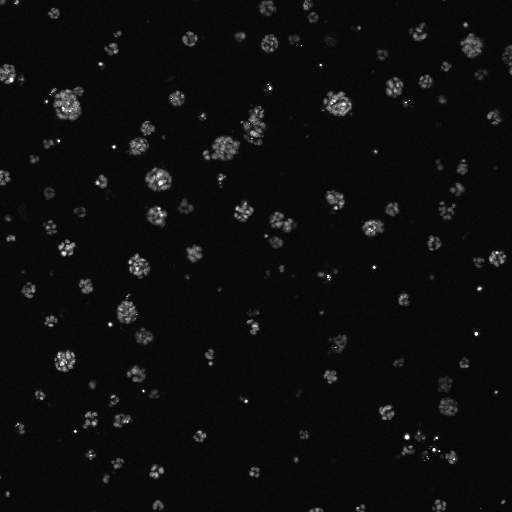

Supplement: Supplementary file 5 — Code EV1 [file MSB-13-955-s005.zip › DeathPro/example_images/150612_OC12_0h_2.1_MIPs/OC12_0h__W0031__P0002_channel0.tif]

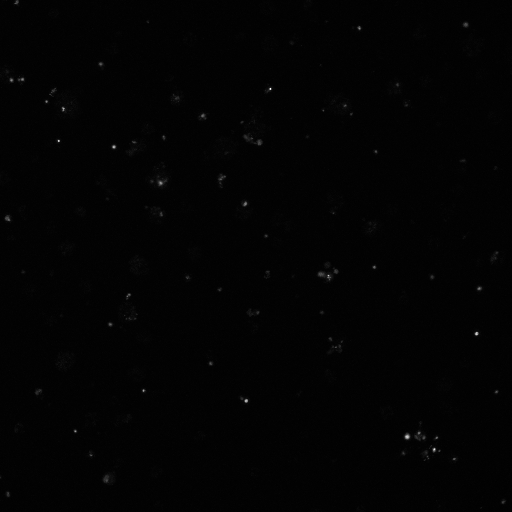

Supplement: Supplementary file 5 — Code EV1 [file MSB-13-955-s005.zip › DeathPro/example_images/150612_OC12_0h_2.1_MIPs/OC12_0h__W0031__P0002_channel1.tif]

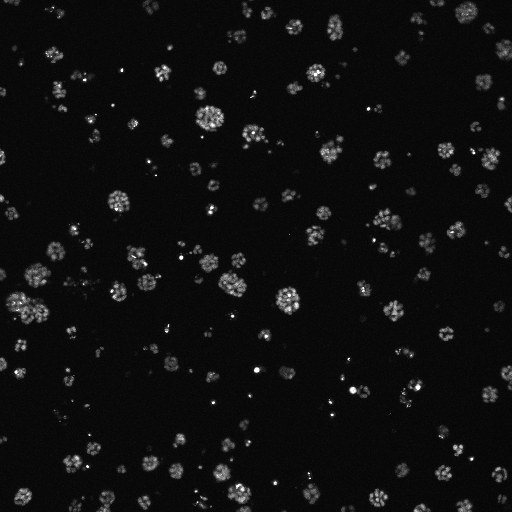

Supplement: Supplementary file 5 — Code EV1 [file MSB-13-955-s005.zip › DeathPro/example_images/150612_OC12_0h_2.1_MIPs/OC12_0h__W0032__P0001_channel0.tif]

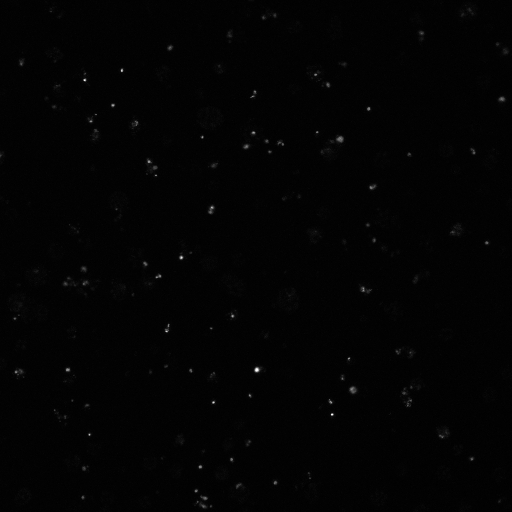

Supplement: Supplementary file 5 — Code EV1 [file MSB-13-955-s005.zip › DeathPro/example_images/150612_OC12_0h_2.1_MIPs/OC12_0h__W0032__P0001_channel1.tif]

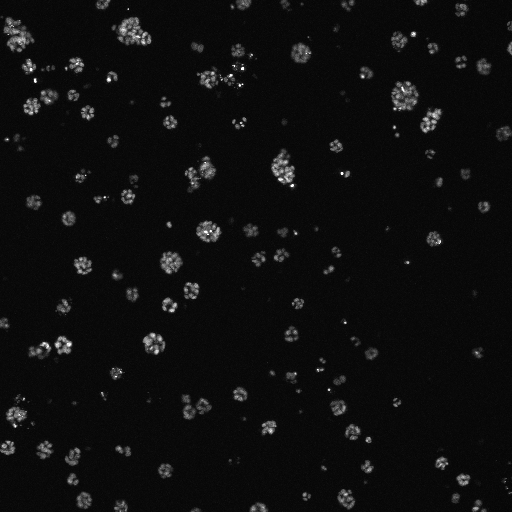

Supplement: Supplementary file 5 — Code EV1 [file MSB-13-955-s005.zip › DeathPro/example_images/150612_OC12_0h_2.1_MIPs/OC12_0h__W0032__P0002_channel0.tif]

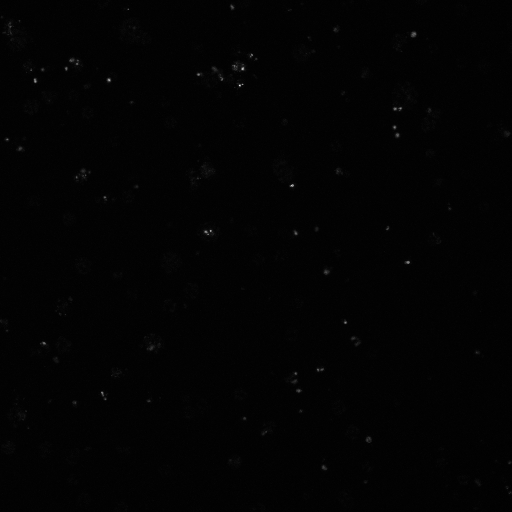

Supplement: Supplementary file 5 — Code EV1 [file MSB-13-955-s005.zip › DeathPro/example_images/150612_OC12_0h_2.1_MIPs/OC12_0h__W0032__P0002_channel1.tif]

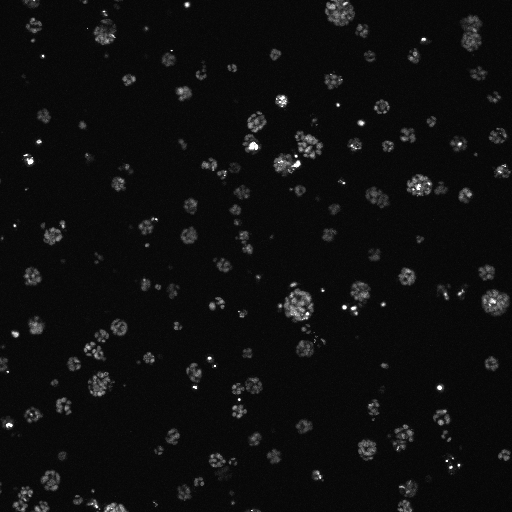

Supplement: Supplementary file 5 — Code EV1 [file MSB-13-955-s005.zip › DeathPro/example_images/150612_OC12_0h_2.1_MIPs/OC12_0h__W0033__P0001_channel0.tif]

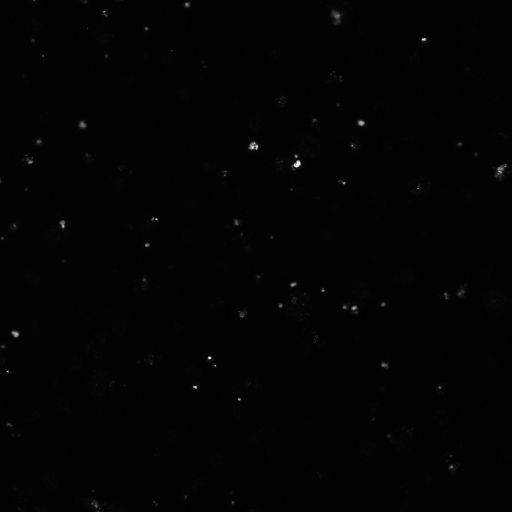

Supplement: Supplementary file 5 — Code EV1 [file MSB-13-955-s005.zip › DeathPro/example_images/150612_OC12_0h_2.1_MIPs/OC12_0h__W0033__P0001_channel1.tif]

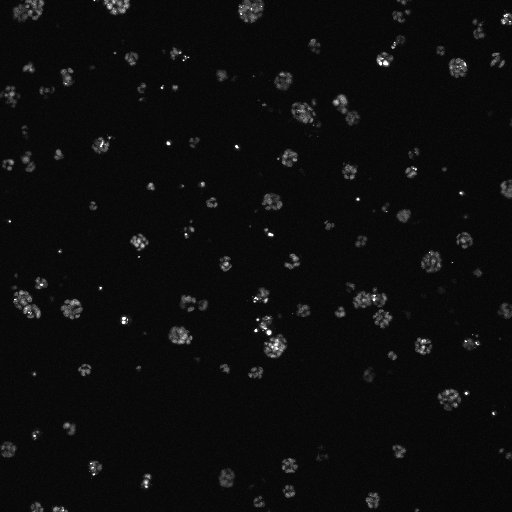

Supplement: Supplementary file 5 — Code EV1 [file MSB-13-955-s005.zip › DeathPro/example_images/150612_OC12_0h_2.1_MIPs/OC12_0h__W0033__P0002_channel0.tif]

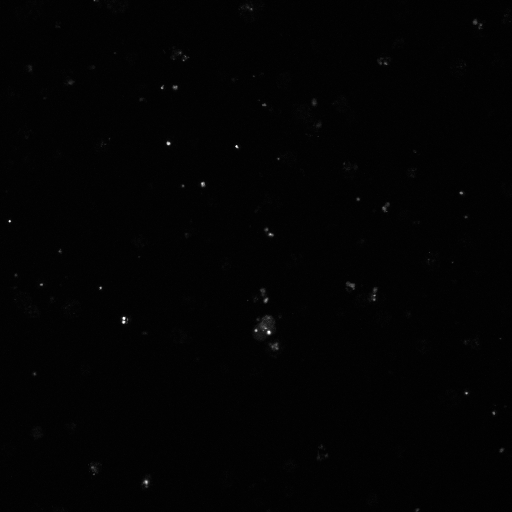

Supplement: Supplementary file 5 — Code EV1 [file MSB-13-955-s005.zip › DeathPro/example_images/150612_OC12_0h_2.1_MIPs/OC12_0h__W0033__P0002_channel1.tif]

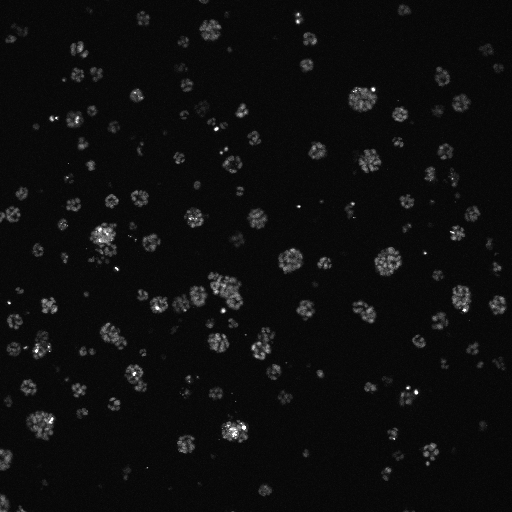

Supplement: Supplementary file 5 — Code EV1 [file MSB-13-955-s005.zip › DeathPro/example_images/150612_OC12_0h_2.1_MIPs/OC12_0h__W0034__P0001_channel0.tif]

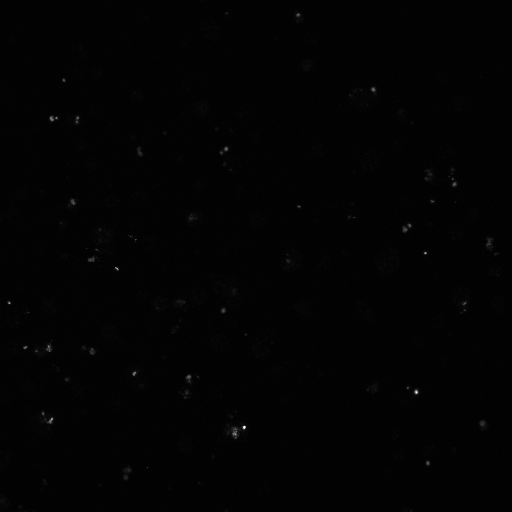

Supplement: Supplementary file 5 — Code EV1 [file MSB-13-955-s005.zip › DeathPro/example_images/150612_OC12_0h_2.1_MIPs/OC12_0h__W0034__P0001_channel1.tif]

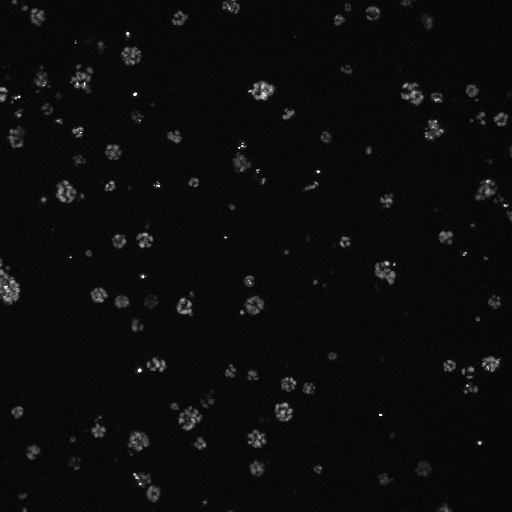

Supplement: Supplementary file 5 — Code EV1 [file MSB-13-955-s005.zip › DeathPro/example_images/150612_OC12_0h_2.1_MIPs/OC12_0h__W0034__P0002_channel0.tif]

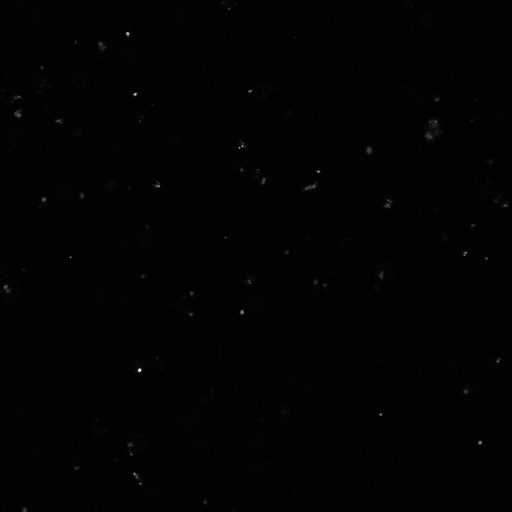

Supplement: Supplementary file 5 — Code EV1 [file MSB-13-955-s005.zip › DeathPro/example_images/150612_OC12_0h_2.1_MIPs/OC12_0h__W0034__P0002_channel1.tif]

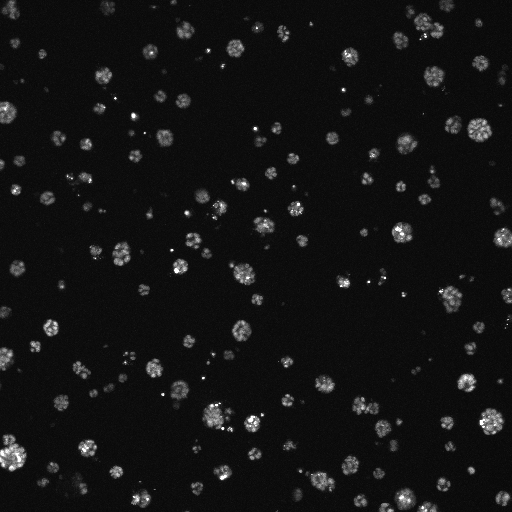

Supplement: Supplementary file 5 — Code EV1 [file MSB-13-955-s005.zip › DeathPro/example_images/150612_OC12_0h_2.1_MIPs/OC12_0h__W0035__P0001_channel0.tif]

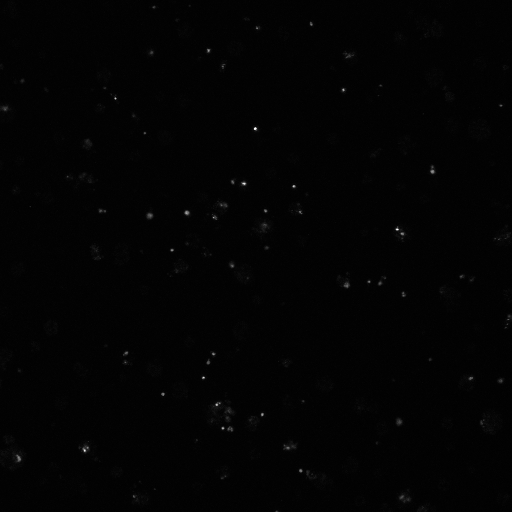

Supplement: Supplementary file 5 — Code EV1 [file MSB-13-955-s005.zip › DeathPro/example_images/150612_OC12_0h_2.1_MIPs/OC12_0h__W0035__P0001_channel1.tif]

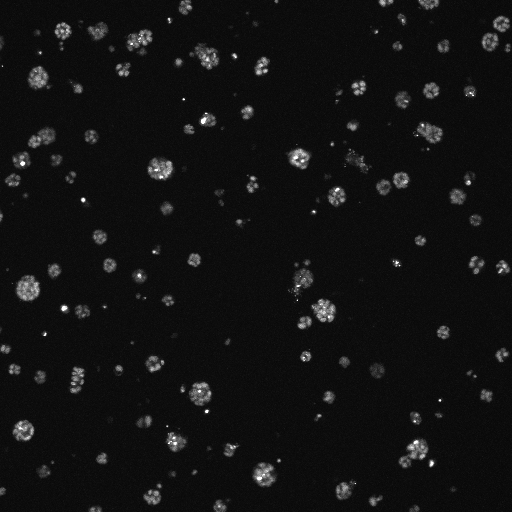

Supplement: Supplementary file 5 — Code EV1 [file MSB-13-955-s005.zip › DeathPro/example_images/150612_OC12_0h_2.1_MIPs/OC12_0h__W0035__P0002_channel0.tif]

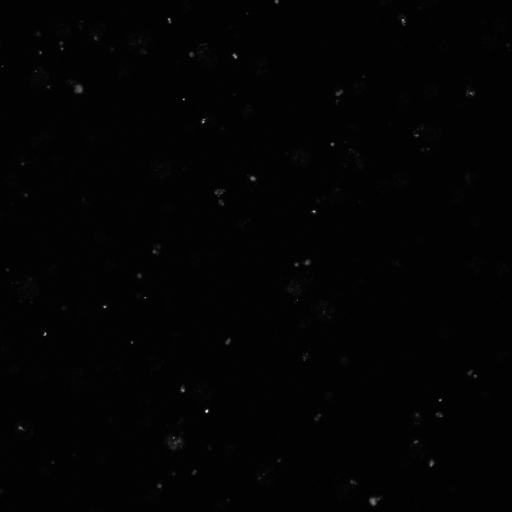

Supplement: Supplementary file 5 — Code EV1 [file MSB-13-955-s005.zip › DeathPro/example_images/150612_OC12_0h_2.1_MIPs/OC12_0h__W0035__P0002_channel1.tif]

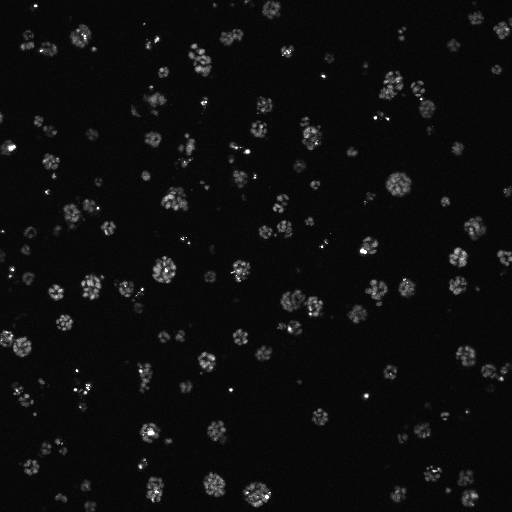

Supplement: Supplementary file 5 — Code EV1 [file MSB-13-955-s005.zip › DeathPro/example_images/150612_OC12_0h_2.1_MIPs/OC12_0h__W0036__P0001_channel0.tif]

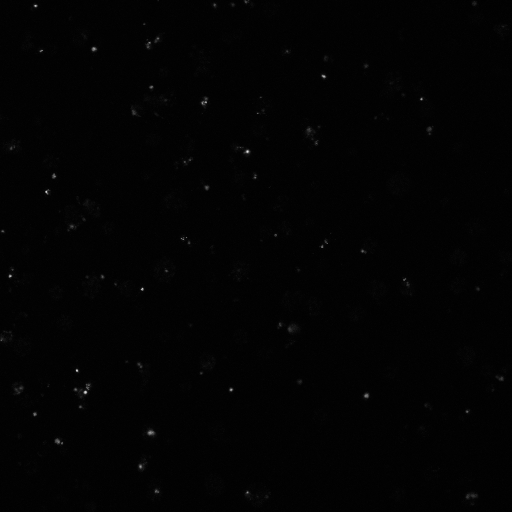

Supplement: Supplementary file 5 — Code EV1 [file MSB-13-955-s005.zip › DeathPro/example_images/150612_OC12_0h_2.1_MIPs/OC12_0h__W0036__P0001_channel1.tif]

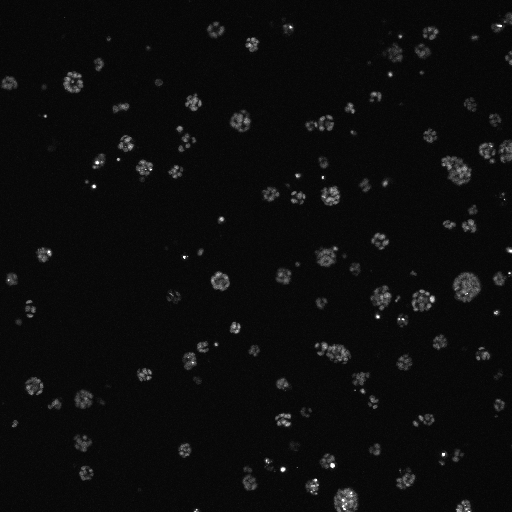

Supplement: Supplementary file 5 — Code EV1 [file MSB-13-955-s005.zip › DeathPro/example_images/150612_OC12_0h_2.1_MIPs/OC12_0h__W0036__P0002_channel0.tif]

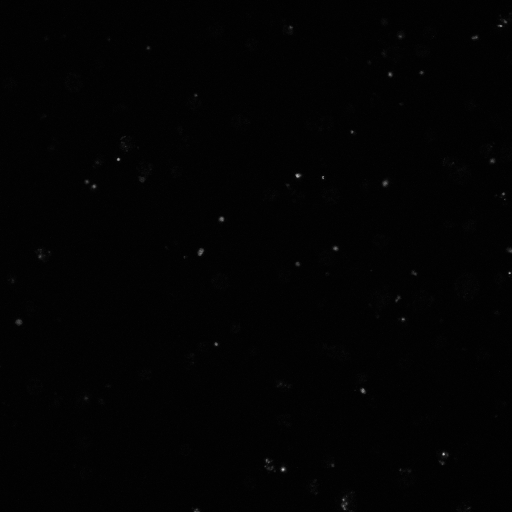

Supplement: Supplementary file 5 — Code EV1 [file MSB-13-955-s005.zip › DeathPro/example_images/150612_OC12_0h_2.1_MIPs/OC12_0h__W0036__P0002_channel1.tif]

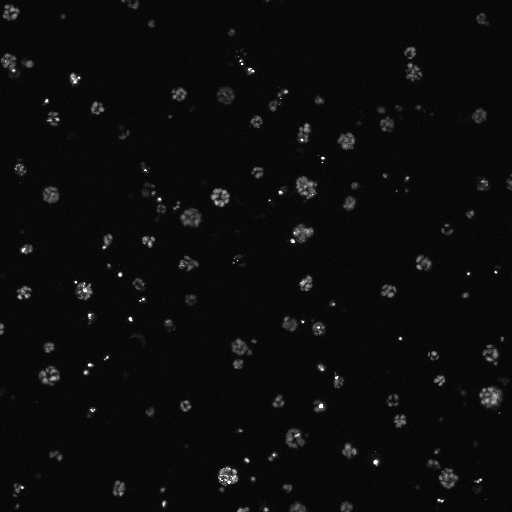

Supplement: Supplementary file 5 — Code EV1 [file MSB-13-955-s005.zip › DeathPro/example_images/150612_OC12_0h_2.1_MIPs/OC12_0h__W0037__P0001_channel0.tif]

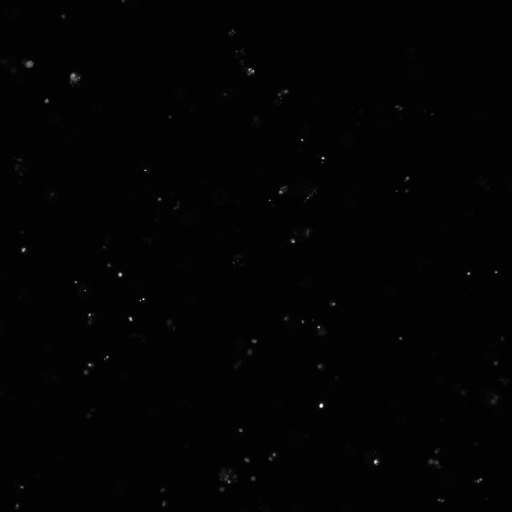

Supplement: Supplementary file 5 — Code EV1 [file MSB-13-955-s005.zip › DeathPro/example_images/150612_OC12_0h_2.1_MIPs/OC12_0h__W0037__P0001_channel1.tif]

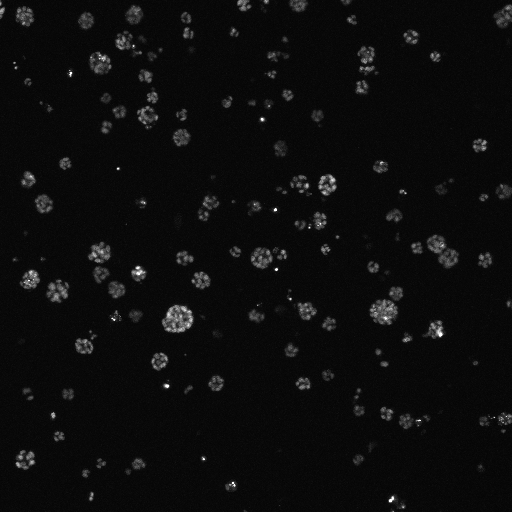

Supplement: Supplementary file 5 — Code EV1 [file MSB-13-955-s005.zip › DeathPro/example_images/150612_OC12_0h_2.1_MIPs/OC12_0h__W0037__P0002_channel0.tif]

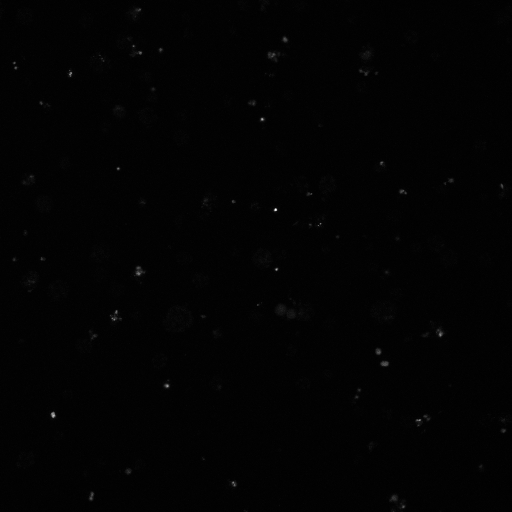

Supplement: Supplementary file 5 — Code EV1 [file MSB-13-955-s005.zip › DeathPro/example_images/150612_OC12_0h_2.1_MIPs/OC12_0h__W0037__P0002_channel1.tif]

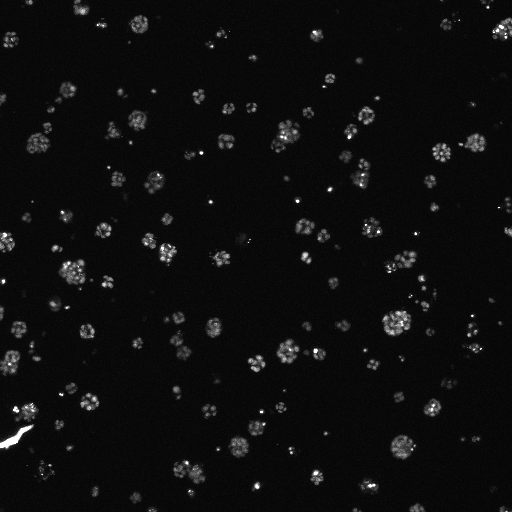

Supplement: Supplementary file 5 — Code EV1 [file MSB-13-955-s005.zip › DeathPro/example_images/150612_OC12_0h_2.1_MIPs/OC12_0h__W0038__P0001_channel0.tif]

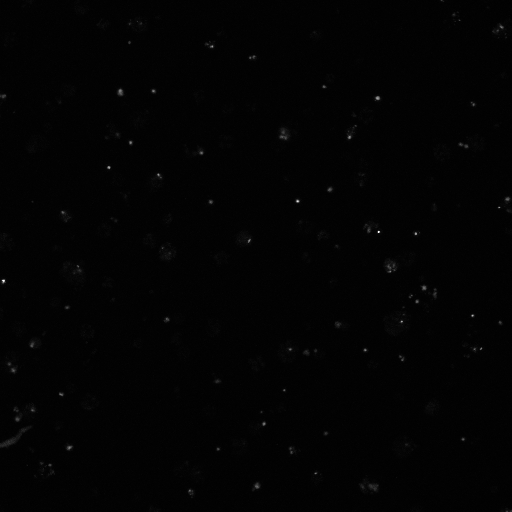

Supplement: Supplementary file 5 — Code EV1 [file MSB-13-955-s005.zip › DeathPro/example_images/150612_OC12_0h_2.1_MIPs/OC12_0h__W0038__P0001_channel1.tif]

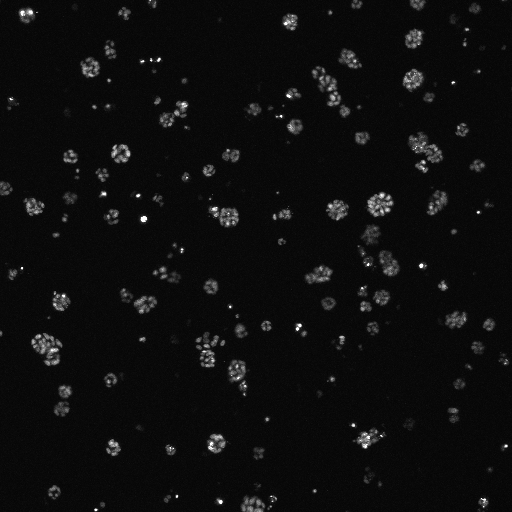

Supplement: Supplementary file 5 — Code EV1 [file MSB-13-955-s005.zip › DeathPro/example_images/150612_OC12_0h_2.1_MIPs/OC12_0h__W0038__P0002_channel0.tif]

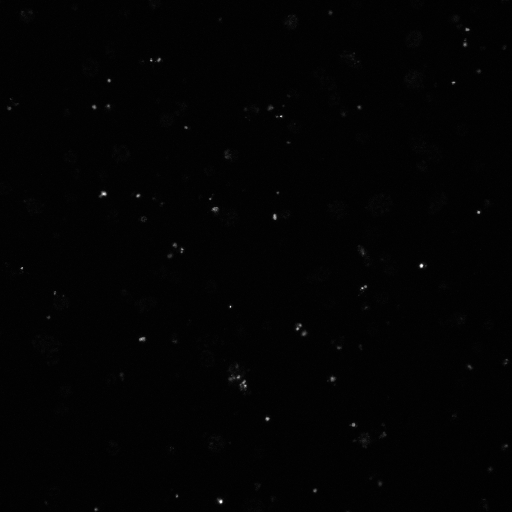

Supplement: Supplementary file 5 — Code EV1 [file MSB-13-955-s005.zip › DeathPro/example_images/150612_OC12_0h_2.1_MIPs/OC12_0h__W0038__P0002_channel1.tif]

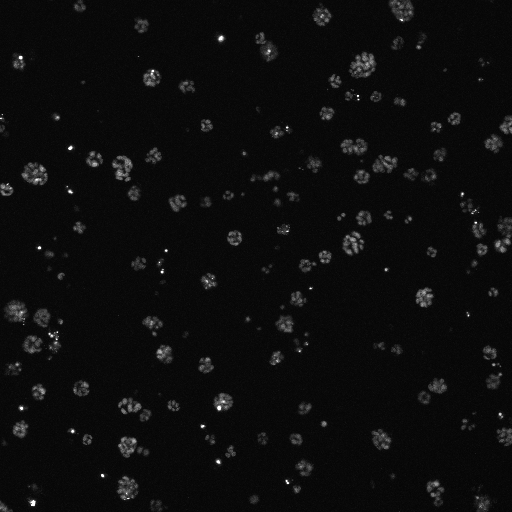

Supplement: Supplementary file 5 — Code EV1 [file MSB-13-955-s005.zip › DeathPro/example_images/150612_OC12_0h_2.1_MIPs/OC12_0h__W0039__P0001_channel0.tif]

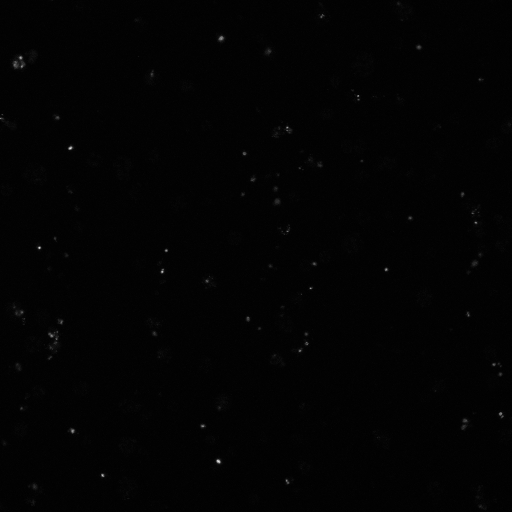

Supplement: Supplementary file 5 — Code EV1 [file MSB-13-955-s005.zip › DeathPro/example_images/150612_OC12_0h_2.1_MIPs/OC12_0h__W0039__P0001_channel1.tif]

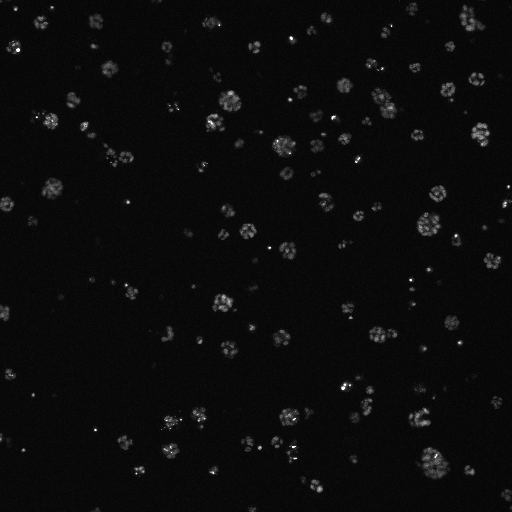

Supplement: Supplementary file 5 — Code EV1 [file MSB-13-955-s005.zip › DeathPro/example_images/150612_OC12_0h_2.1_MIPs/OC12_0h__W0039__P0002_channel0.tif]

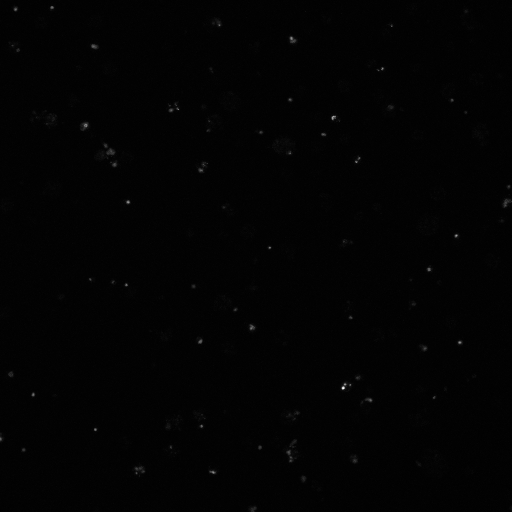

Supplement: Supplementary file 5 — Code EV1 [file MSB-13-955-s005.zip › DeathPro/example_images/150612_OC12_0h_2.1_MIPs/OC12_0h__W0039__P0002_channel1.tif]

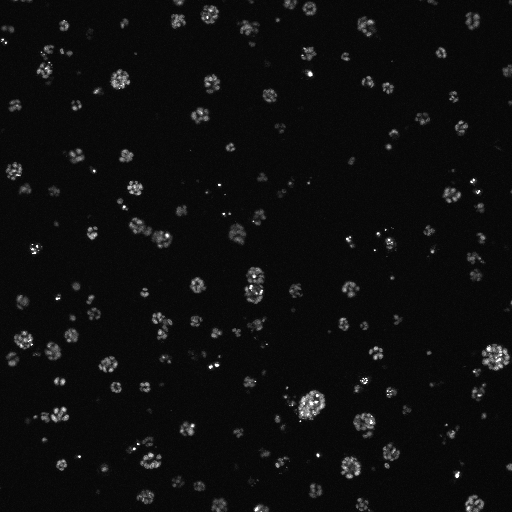

Supplement: Supplementary file 5 — Code EV1 [file MSB-13-955-s005.zip › DeathPro/example_images/150612_OC12_0h_2.1_MIPs/OC12_0h__W0040__P0001_channel0.tif]

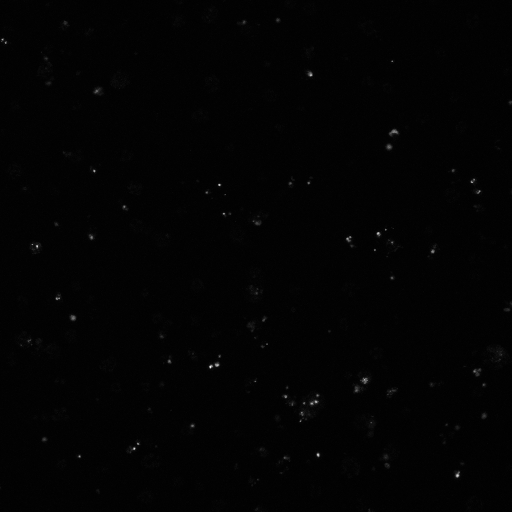

Supplement: Supplementary file 5 — Code EV1 [file MSB-13-955-s005.zip › DeathPro/example_images/150612_OC12_0h_2.1_MIPs/OC12_0h__W0040__P0001_channel1.tif]

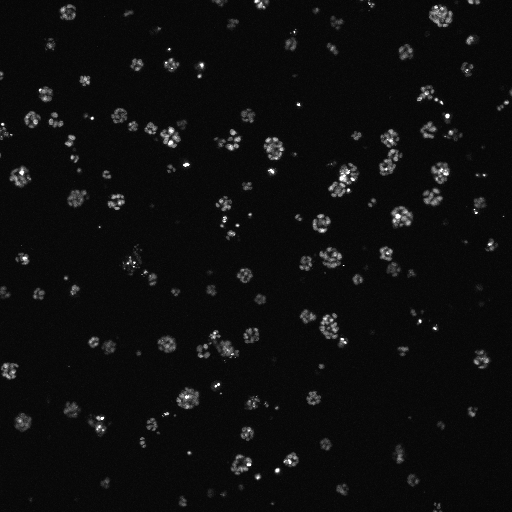

Supplement: Supplementary file 5 — Code EV1 [file MSB-13-955-s005.zip › DeathPro/example_images/150612_OC12_0h_2.1_MIPs/OC12_0h__W0040__P0002_channel0.tif]

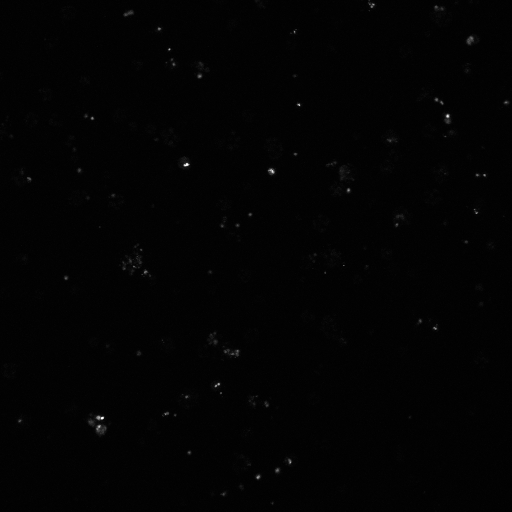

Supplement: Supplementary file 5 — Code EV1 [file MSB-13-955-s005.zip › DeathPro/example_images/150612_OC12_0h_2.1_MIPs/OC12_0h__W0040__P0002_channel1.tif]

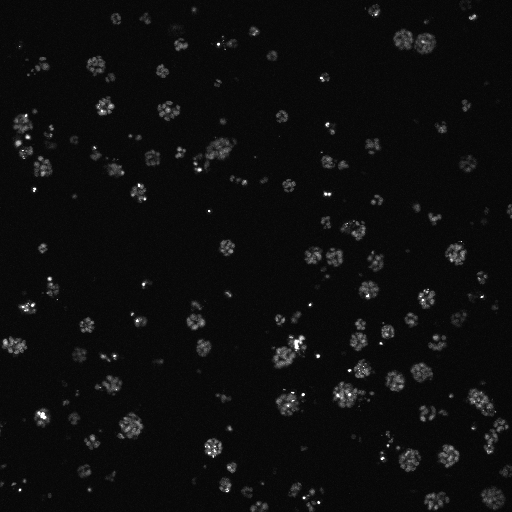

Supplement: Supplementary file 5 — Code EV1 [file MSB-13-955-s005.zip › DeathPro/example_images/150612_OC12_0h_2.1_MIPs/OC12_0h__W0041__P0001_channel0.tif]

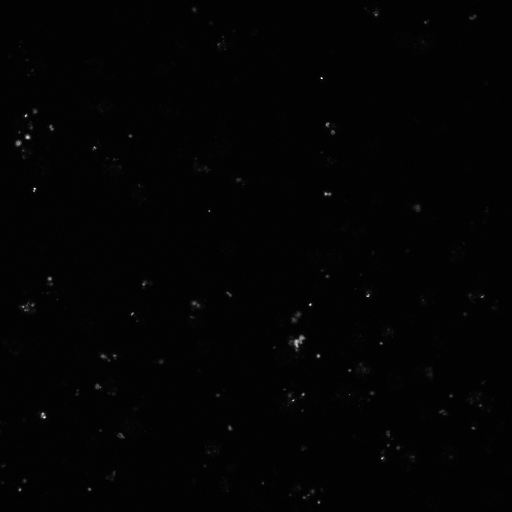

Supplement: Supplementary file 5 — Code EV1 [file MSB-13-955-s005.zip › DeathPro/example_images/150612_OC12_0h_2.1_MIPs/OC12_0h__W0041__P0001_channel1.tif]

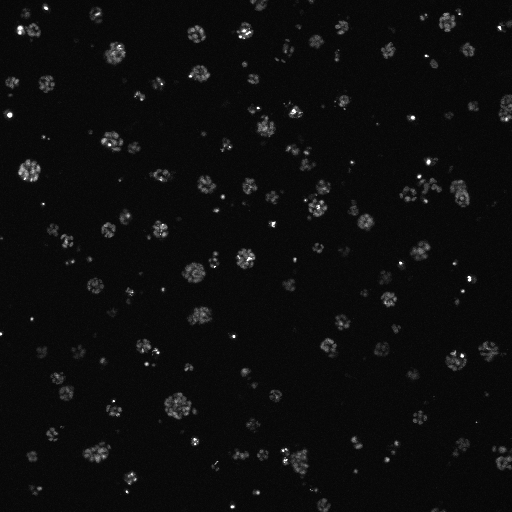

Supplement: Supplementary file 5 — Code EV1 [file MSB-13-955-s005.zip › DeathPro/example_images/150612_OC12_0h_2.1_MIPs/OC12_0h__W0041__P0002_channel0.tif]

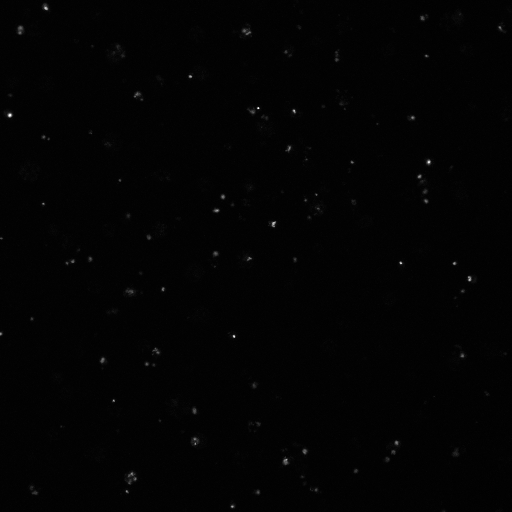

Supplement: Supplementary file 5 — Code EV1 [file MSB-13-955-s005.zip › DeathPro/example_images/150612_OC12_0h_2.1_MIPs/OC12_0h__W0041__P0002_channel1.tif]

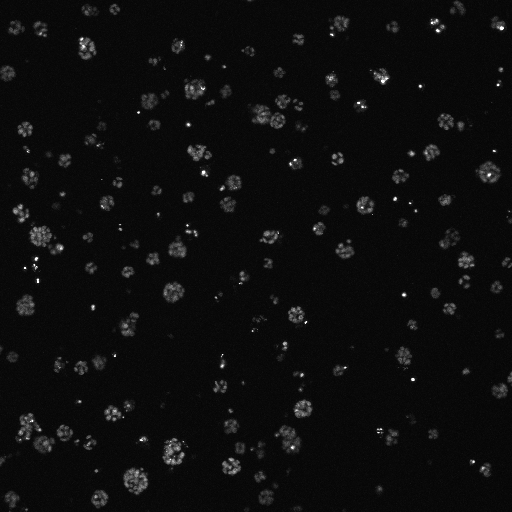

Supplement: Supplementary file 5 — Code EV1 [file MSB-13-955-s005.zip › DeathPro/example_images/150612_OC12_0h_2.1_MIPs/OC12_0h__W0042__P0001_channel0.tif]

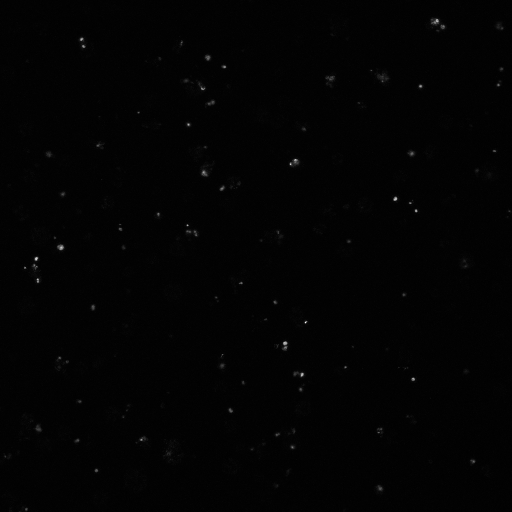

Supplement: Supplementary file 5 — Code EV1 [file MSB-13-955-s005.zip › DeathPro/example_images/150612_OC12_0h_2.1_MIPs/OC12_0h__W0042__P0001_channel1.tif]

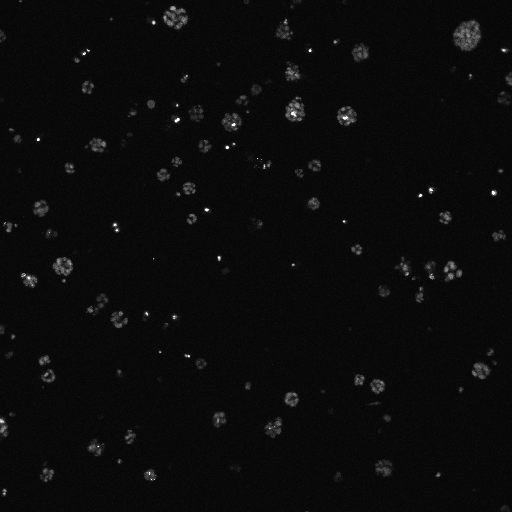

Supplement: Supplementary file 5 — Code EV1 [file MSB-13-955-s005.zip › DeathPro/example_images/150612_OC12_0h_2.1_MIPs/OC12_0h__W0042__P0002_channel0.tif]

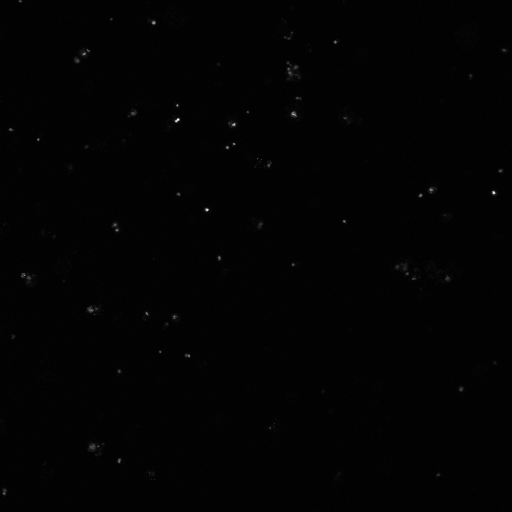

Supplement: Supplementary file 5 — Code EV1 [file MSB-13-955-s005.zip › DeathPro/example_images/150612_OC12_0h_2.1_MIPs/OC12_0h__W0042__P0002_channel1.tif]

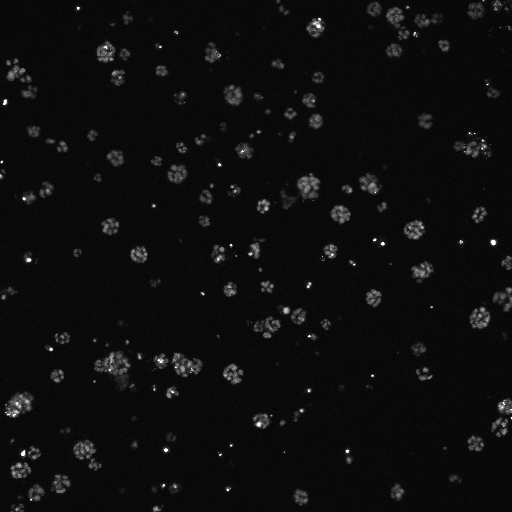

Supplement: Supplementary file 5 — Code EV1 [file MSB-13-955-s005.zip › DeathPro/example_images/150612_OC12_0h_2.1_MIPs/OC12_0h__W0043__P0001_channel0.tif]

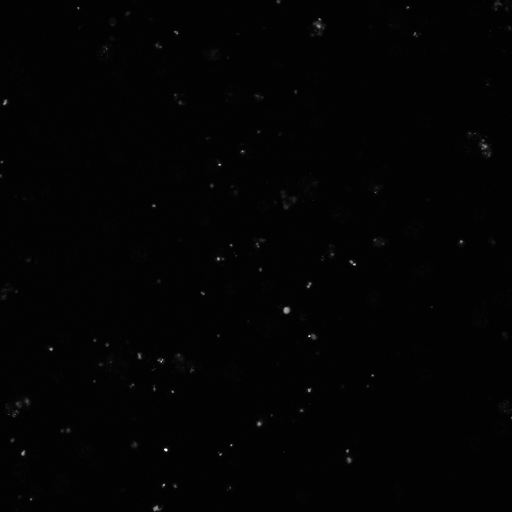

Supplement: Supplementary file 5 — Code EV1 [file MSB-13-955-s005.zip › DeathPro/example_images/150612_OC12_0h_2.1_MIPs/OC12_0h__W0043__P0001_channel1.tif]

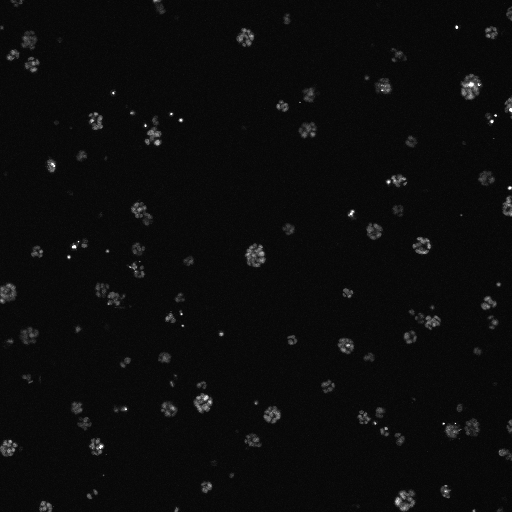

Supplement: Supplementary file 5 — Code EV1 [file MSB-13-955-s005.zip › DeathPro/example_images/150612_OC12_0h_2.1_MIPs/OC12_0h__W0043__P0002_channel0.tif]

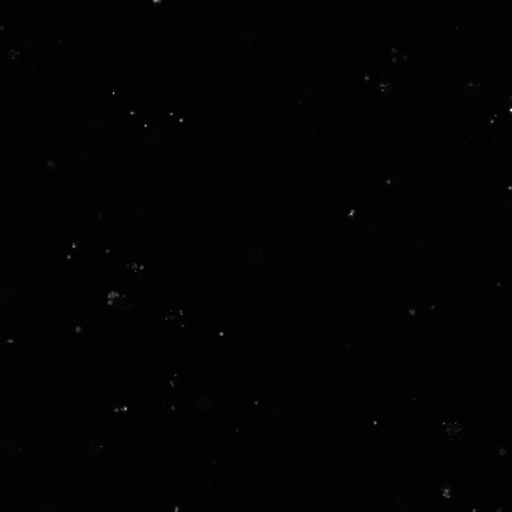

Supplement: Supplementary file 5 — Code EV1 [file MSB-13-955-s005.zip › DeathPro/example_images/150612_OC12_0h_2.1_MIPs/OC12_0h__W0043__P0002_channel1.tif]

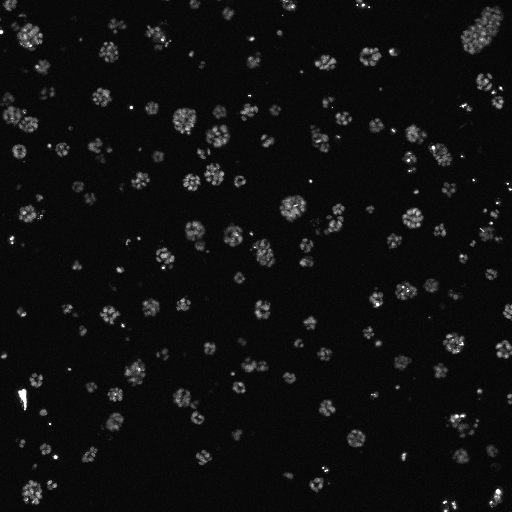

Supplement: Supplementary file 5 — Code EV1 [file MSB-13-955-s005.zip › DeathPro/example_images/150612_OC12_0h_2.1_MIPs/OC12_0h__W0044__P0001_channel0.tif]

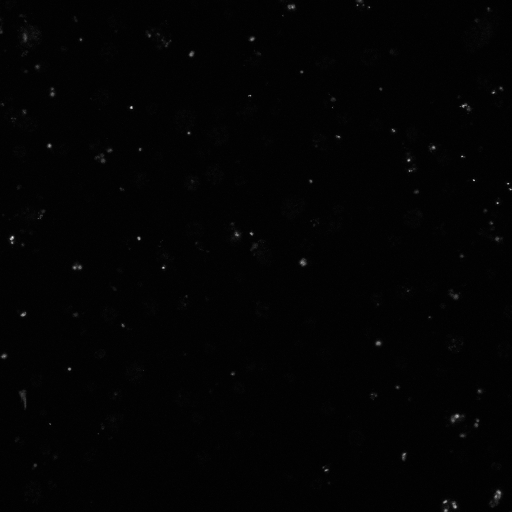

Supplement: Supplementary file 5 — Code EV1 [file MSB-13-955-s005.zip › DeathPro/example_images/150612_OC12_0h_2.1_MIPs/OC12_0h__W0044__P0001_channel1.tif]

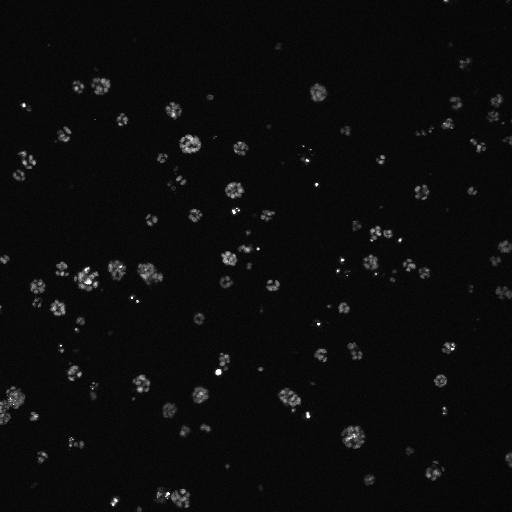

Supplement: Supplementary file 5 — Code EV1 [file MSB-13-955-s005.zip › DeathPro/example_images/150612_OC12_0h_2.1_MIPs/OC12_0h__W0044__P0002_channel0.tif]

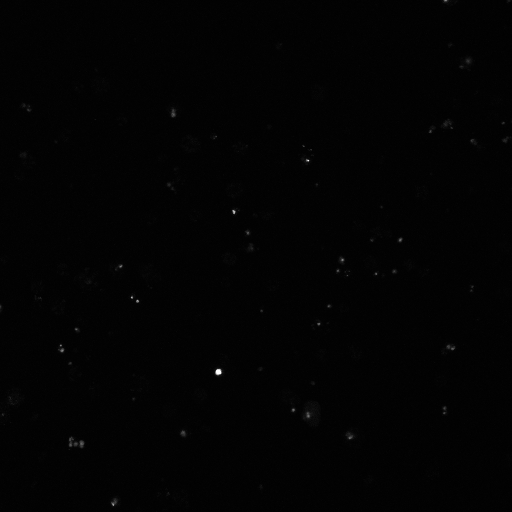

Supplement: Supplementary file 5 — Code EV1 [file MSB-13-955-s005.zip › DeathPro/example_images/150612_OC12_0h_2.1_MIPs/OC12_0h__W0044__P0002_channel1.tif]

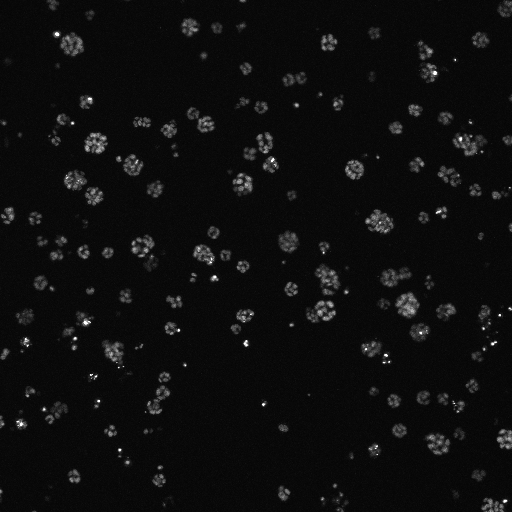

Supplement: Supplementary file 5 — Code EV1 [file MSB-13-955-s005.zip › DeathPro/example_images/150612_OC12_0h_2.1_MIPs/OC12_0h__W0045__P0001_channel0.tif]

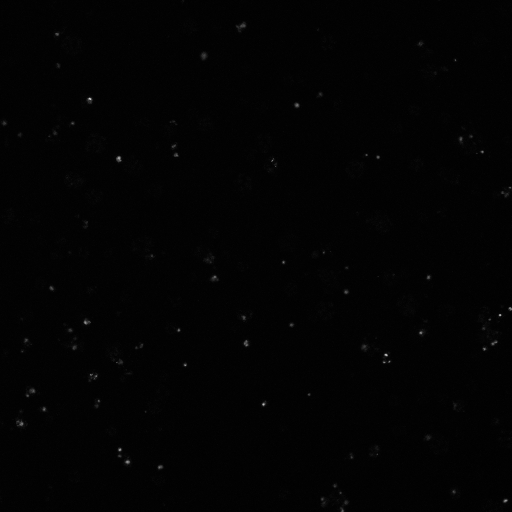

Supplement: Supplementary file 5 — Code EV1 [file MSB-13-955-s005.zip › DeathPro/example_images/150612_OC12_0h_2.1_MIPs/OC12_0h__W0045__P0001_channel1.tif]

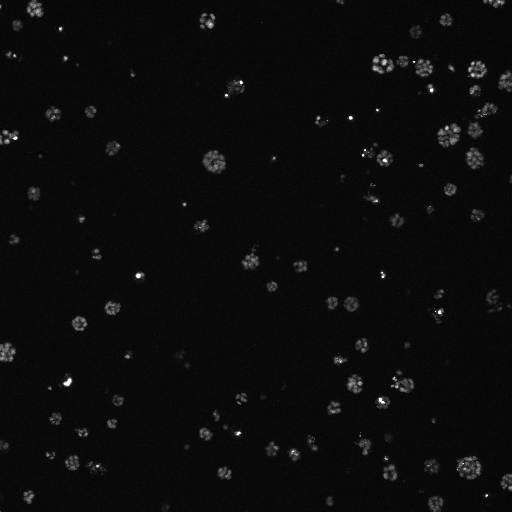

Supplement: Supplementary file 5 — Code EV1 [file MSB-13-955-s005.zip › DeathPro/example_images/150612_OC12_0h_2.1_MIPs/OC12_0h__W0045__P0002_channel0.tif]

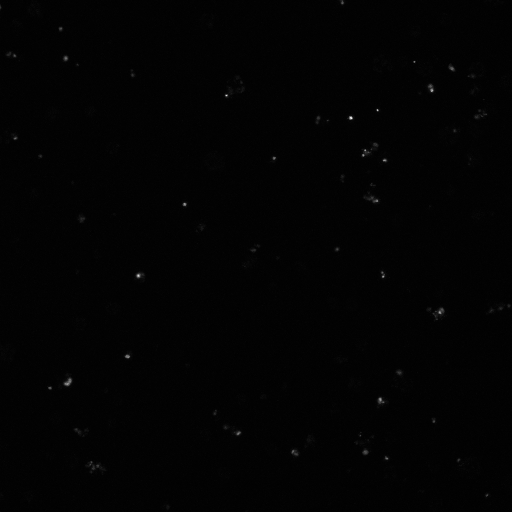

Supplement: Supplementary file 5 — Code EV1 [file MSB-13-955-s005.zip › DeathPro/example_images/150612_OC12_0h_2.1_MIPs/OC12_0h__W0045__P0002_channel1.tif]

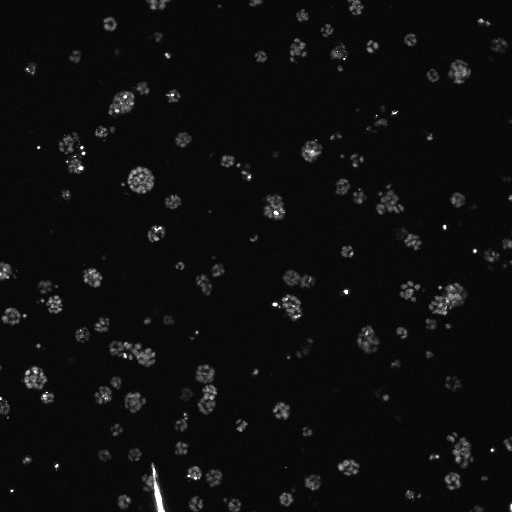

Supplement: Supplementary file 5 — Code EV1 [file MSB-13-955-s005.zip › DeathPro/example_images/150612_OC12_0h_2.1_MIPs/OC12_0h__W0046__P0001_channel0.tif]

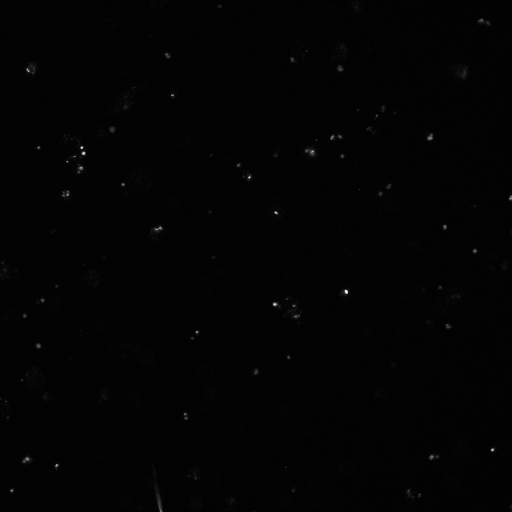

Supplement: Supplementary file 5 — Code EV1 [file MSB-13-955-s005.zip › DeathPro/example_images/150612_OC12_0h_2.1_MIPs/OC12_0h__W0046__P0001_channel1.tif]

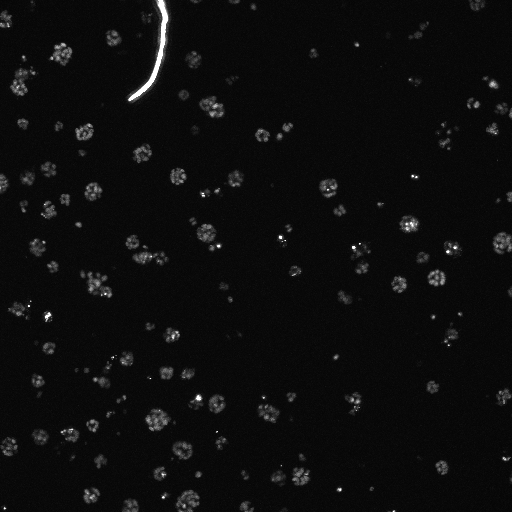

Supplement: Supplementary file 5 — Code EV1 [file MSB-13-955-s005.zip › DeathPro/example_images/150612_OC12_0h_2.1_MIPs/OC12_0h__W0046__P0002_channel0.tif]

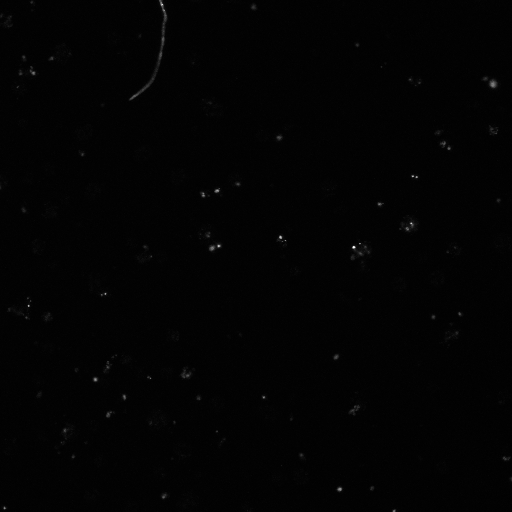

Supplement: Supplementary file 5 — Code EV1 [file MSB-13-955-s005.zip › DeathPro/example_images/150612_OC12_0h_2.1_MIPs/OC12_0h__W0046__P0002_channel1.tif]

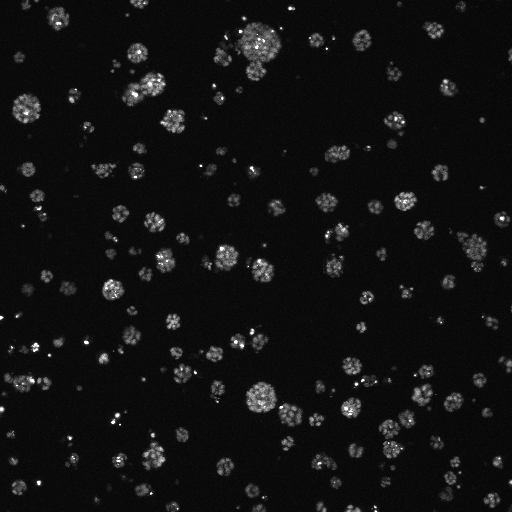

Supplement: Supplementary file 5 — Code EV1 [file MSB-13-955-s005.zip › DeathPro/example_images/150612_OC12_0h_2.1_MIPs/OC12_0h__W0047__P0001_channel0.tif]

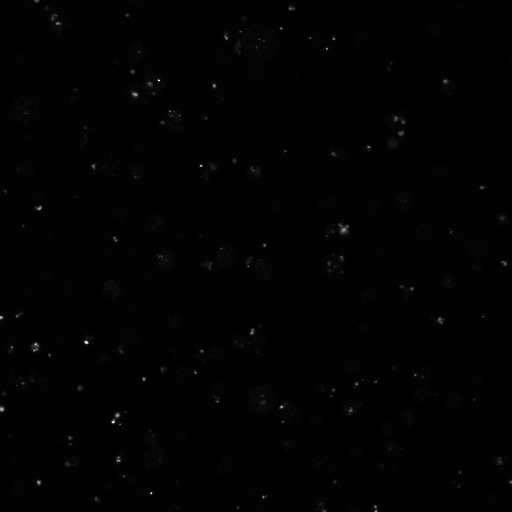

Supplement: Supplementary file 5 — Code EV1 [file MSB-13-955-s005.zip › DeathPro/example_images/150612_OC12_0h_2.1_MIPs/OC12_0h__W0047__P0001_channel1.tif]

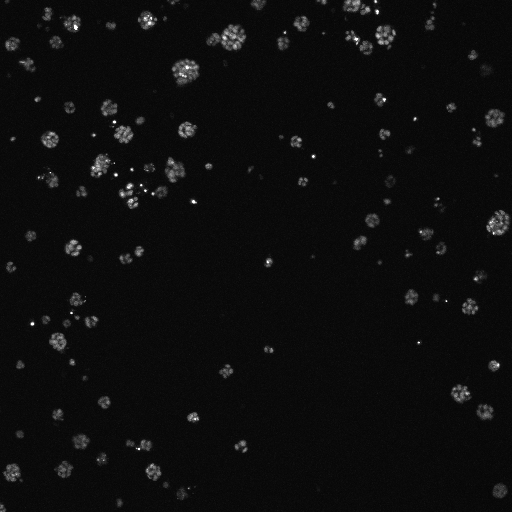

Supplement: Supplementary file 5 — Code EV1 [file MSB-13-955-s005.zip › DeathPro/example_images/150612_OC12_0h_2.1_MIPs/OC12_0h__W0047__P0002_channel0.tif]

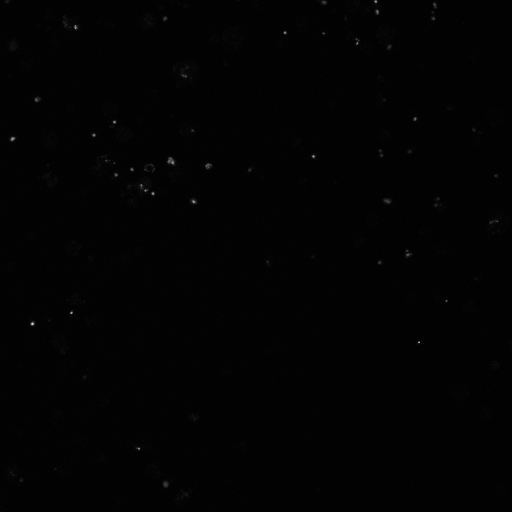

Supplement: Supplementary file 5 — Code EV1 [file MSB-13-955-s005.zip › DeathPro/example_images/150612_OC12_0h_2.1_MIPs/OC12_0h__W0047__P0002_channel1.tif]

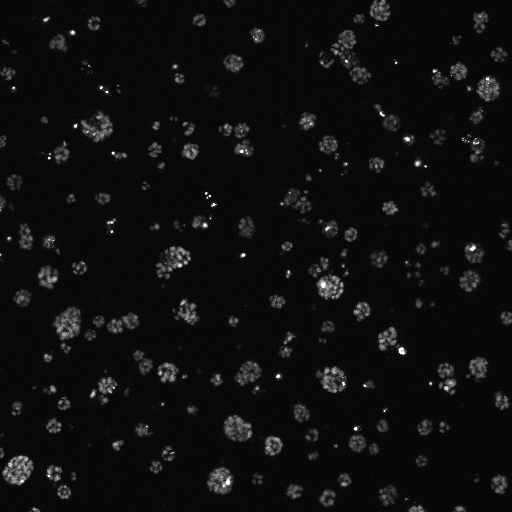

Supplement: Supplementary file 5 — Code EV1 [file MSB-13-955-s005.zip › DeathPro/example_images/150612_OC12_0h_2.1_MIPs/OC12_0h__W0048__P0001_channel0.tif]

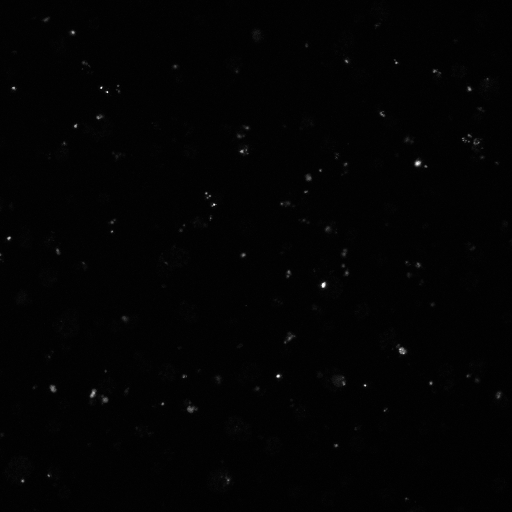

Supplement: Supplementary file 5 — Code EV1 [file MSB-13-955-s005.zip › DeathPro/example_images/150612_OC12_0h_2.1_MIPs/OC12_0h__W0048__P0001_channel1.tif]

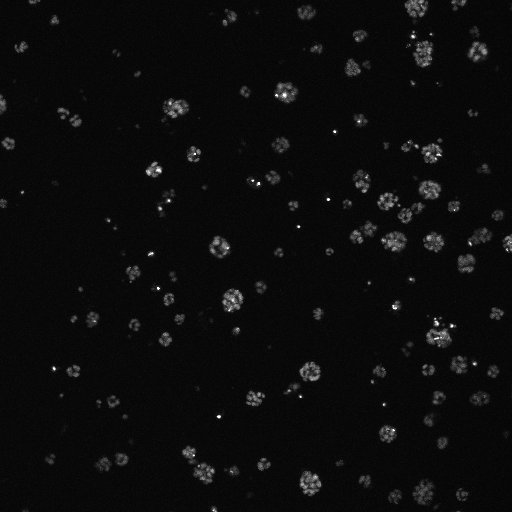

Supplement: Supplementary file 5 — Code EV1 [file MSB-13-955-s005.zip › DeathPro/example_images/150612_OC12_0h_2.1_MIPs/OC12_0h__W0048__P0002_channel0.tif]

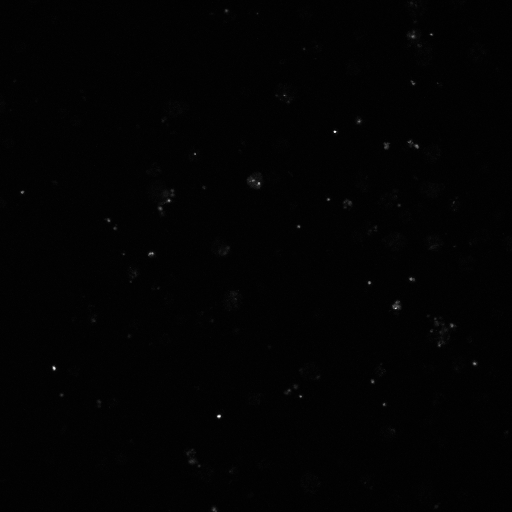

Supplement: Supplementary file 5 — Code EV1 [file MSB-13-955-s005.zip › DeathPro/example_images/150612_OC12_0h_2.1_MIPs/OC12_0h__W0048__P0002_channel1.tif]

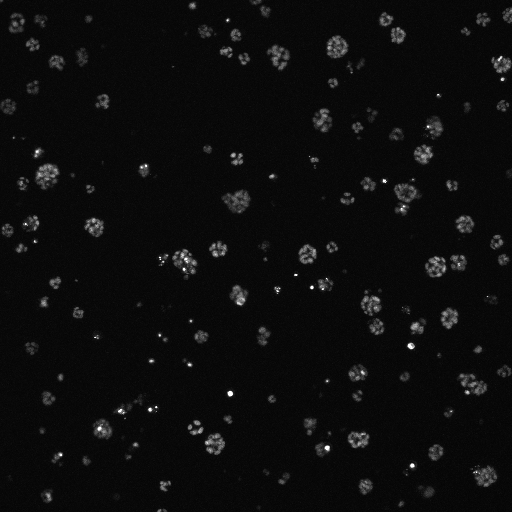

Supplement: Supplementary file 5 — Code EV1 [file MSB-13-955-s005.zip › DeathPro/example_images/150612_OC12_0h_2.1_MIPs/OC12_0h__W0049__P0001_channel0.tif]

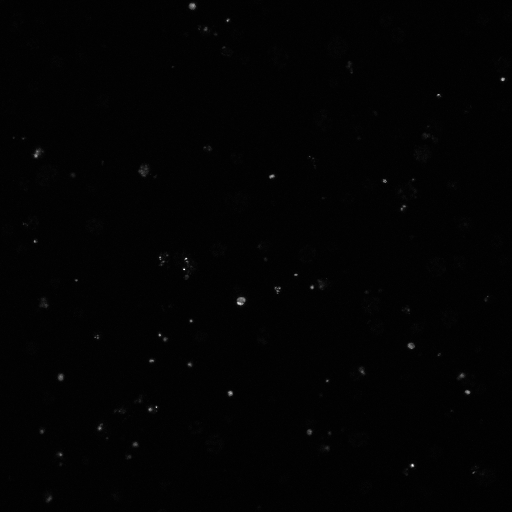

Supplement: Supplementary file 5 — Code EV1 [file MSB-13-955-s005.zip › DeathPro/example_images/150612_OC12_0h_2.1_MIPs/OC12_0h__W0049__P0001_channel1.tif]

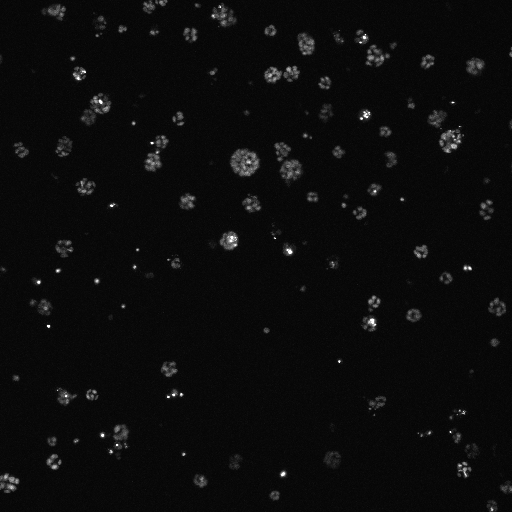

Supplement: Supplementary file 5 — Code EV1 [file MSB-13-955-s005.zip › DeathPro/example_images/150612_OC12_0h_2.1_MIPs/OC12_0h__W0049__P0002_channel0.tif]

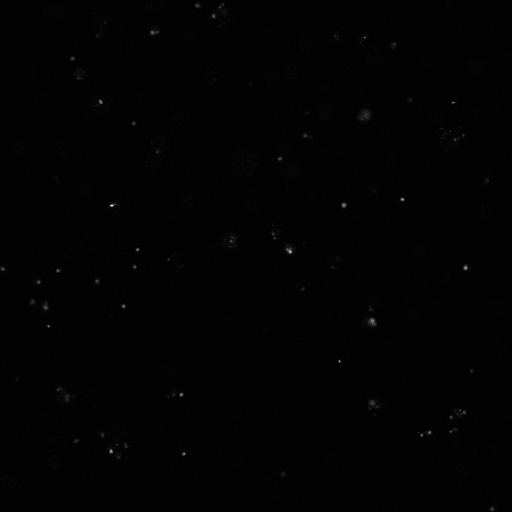

Supplement: Supplementary file 5 — Code EV1 [file MSB-13-955-s005.zip › DeathPro/example_images/150612_OC12_0h_2.1_MIPs/OC12_0h__W0049__P0002_channel1.tif]
