# Supplementary figures and images for: Screening drug effects in patient‐derived cancer cells links organoid responses to genome alterations (part 1 of 2)
Source: Mol Syst Biol. 2017 Nov 27;13(11):955. doi: 10.15252/msb.20177697 (PMC5731348; doi:10.15252/msb.20177697)

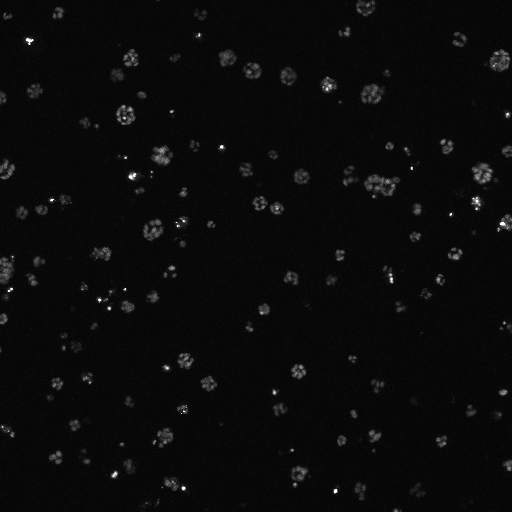

Supplement: Supplementary file 5 — Code EV1 [file MSB-13-955-s005.zip › DeathPro/example_images/150612_OC12_0h_2.1_MIPs/OC12_0h__W0001__P0001_channel0.tif]

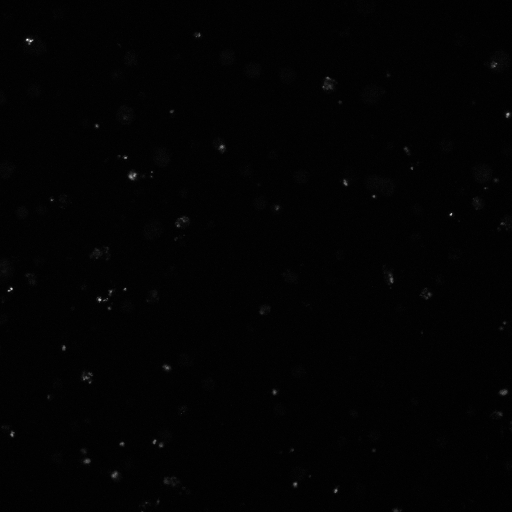

Supplement: Supplementary file 5 — Code EV1 [file MSB-13-955-s005.zip › DeathPro/example_images/150612_OC12_0h_2.1_MIPs/OC12_0h__W0001__P0001_channel1.tif]

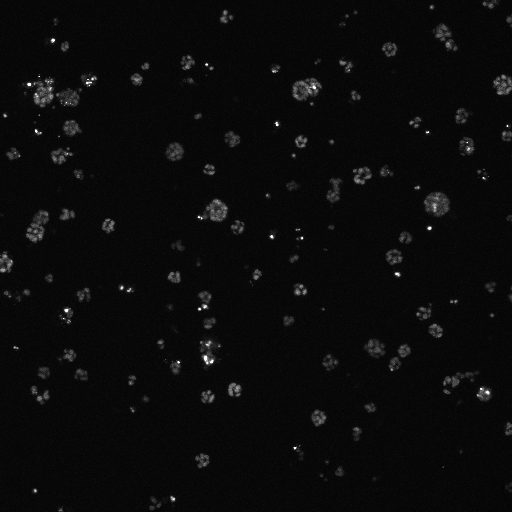

Supplement: Supplementary file 5 — Code EV1 [file MSB-13-955-s005.zip › DeathPro/example_images/150612_OC12_0h_2.1_MIPs/OC12_0h__W0001__P0002_channel0.tif]

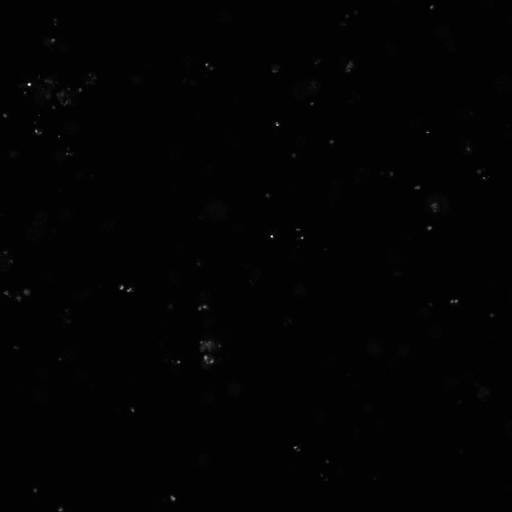

Supplement: Supplementary file 5 — Code EV1 [file MSB-13-955-s005.zip › DeathPro/example_images/150612_OC12_0h_2.1_MIPs/OC12_0h__W0001__P0002_channel1.tif]

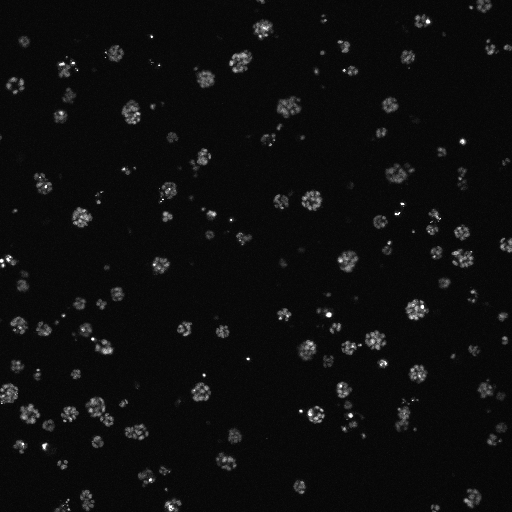

Supplement: Supplementary file 5 — Code EV1 [file MSB-13-955-s005.zip › DeathPro/example_images/150612_OC12_0h_2.1_MIPs/OC12_0h__W0002__P0001_channel0.tif]

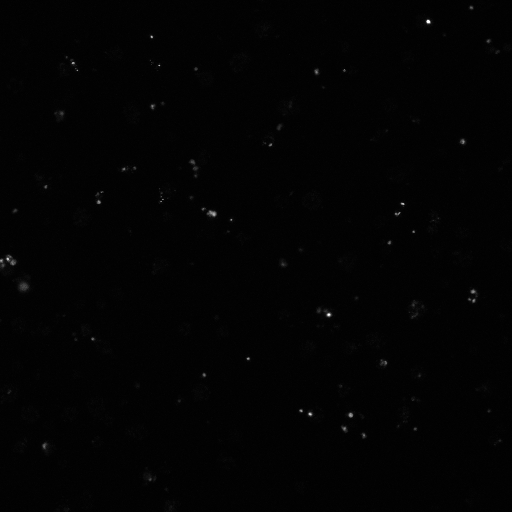

Supplement: Supplementary file 5 — Code EV1 [file MSB-13-955-s005.zip › DeathPro/example_images/150612_OC12_0h_2.1_MIPs/OC12_0h__W0002__P0001_channel1.tif]

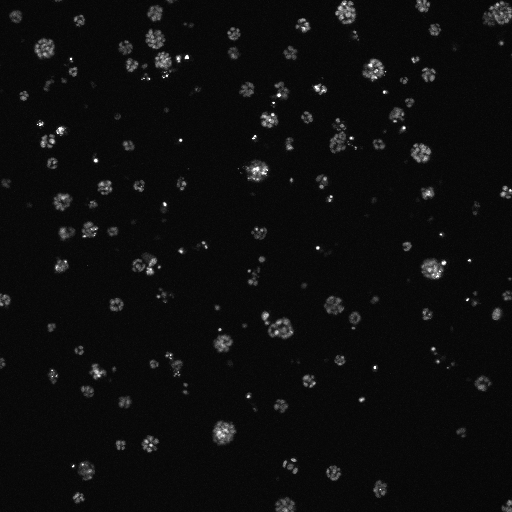

Supplement: Supplementary file 5 — Code EV1 [file MSB-13-955-s005.zip › DeathPro/example_images/150612_OC12_0h_2.1_MIPs/OC12_0h__W0002__P0002_channel0.tif]

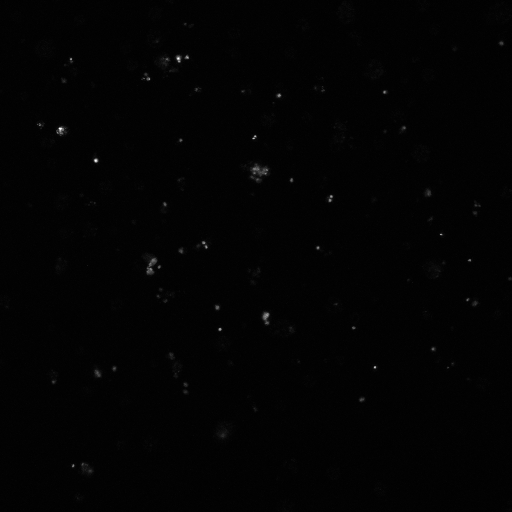

Supplement: Supplementary file 5 — Code EV1 [file MSB-13-955-s005.zip › DeathPro/example_images/150612_OC12_0h_2.1_MIPs/OC12_0h__W0002__P0002_channel1.tif]

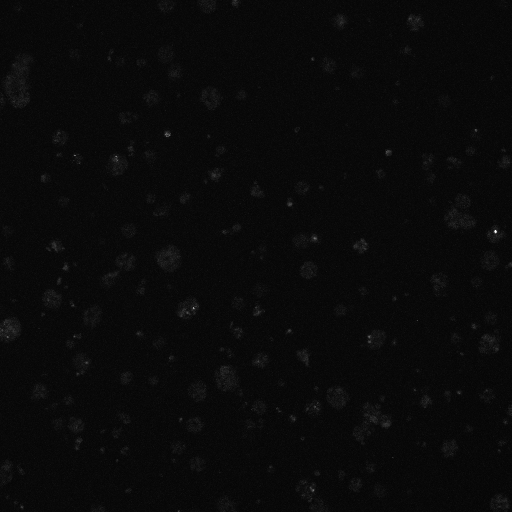

Supplement: Supplementary file 5 — Code EV1 [file MSB-13-955-s005.zip › DeathPro/example_images/150612_OC12_0h_2.1_MIPs/OC12_0h__W0003__P0001_channel0.tif]

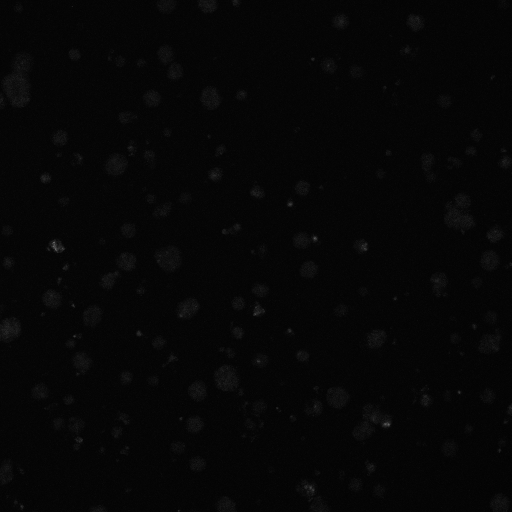

Supplement: Supplementary file 5 — Code EV1 [file MSB-13-955-s005.zip › DeathPro/example_images/150612_OC12_0h_2.1_MIPs/OC12_0h__W0003__P0001_channel1.tif]

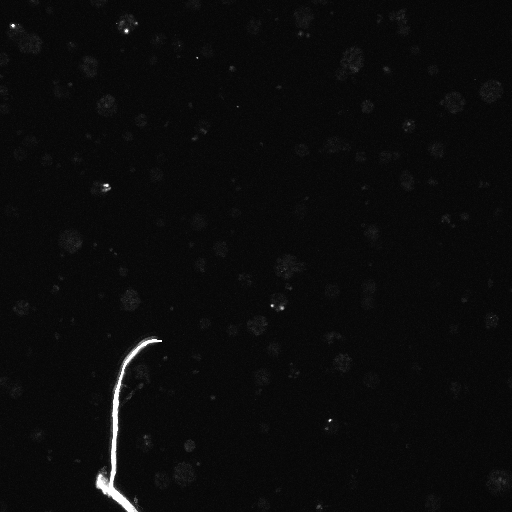

Supplement: Supplementary file 5 — Code EV1 [file MSB-13-955-s005.zip › DeathPro/example_images/150612_OC12_0h_2.1_MIPs/OC12_0h__W0003__P0002_channel0.tif]

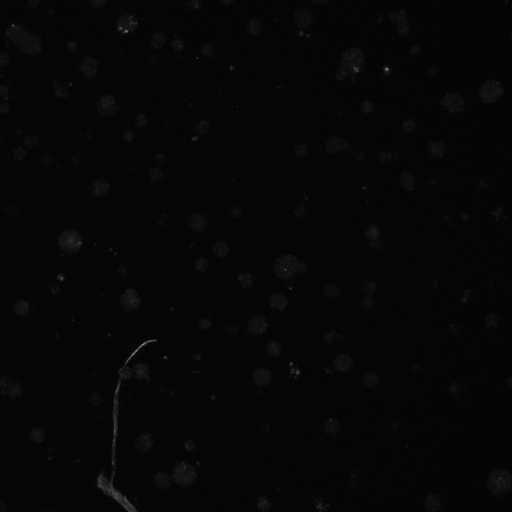

Supplement: Supplementary file 5 — Code EV1 [file MSB-13-955-s005.zip › DeathPro/example_images/150612_OC12_0h_2.1_MIPs/OC12_0h__W0003__P0002_channel1.tif]

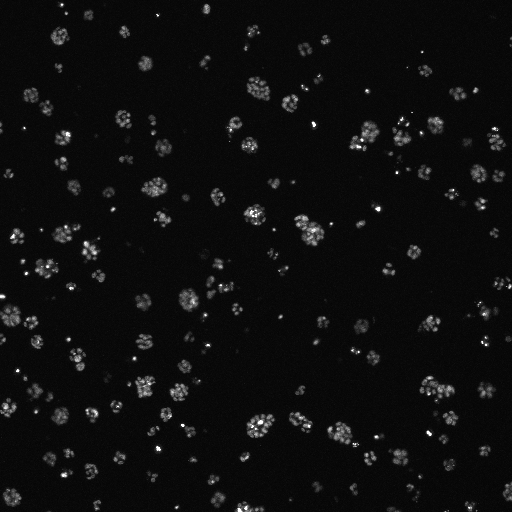

Supplement: Supplementary file 5 — Code EV1 [file MSB-13-955-s005.zip › DeathPro/example_images/150612_OC12_0h_2.1_MIPs/OC12_0h__W0004__P0001_channel0.tif]

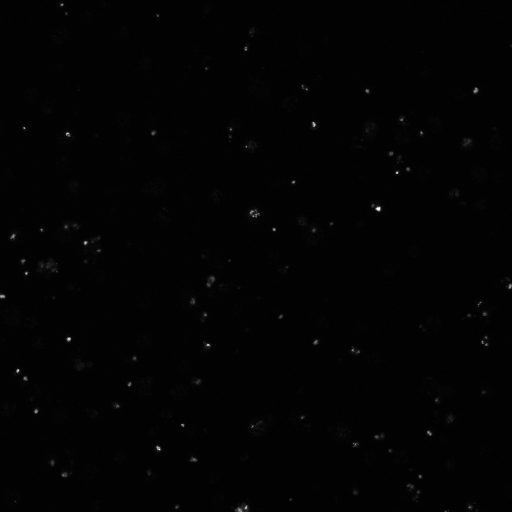

Supplement: Supplementary file 5 — Code EV1 [file MSB-13-955-s005.zip › DeathPro/example_images/150612_OC12_0h_2.1_MIPs/OC12_0h__W0004__P0001_channel1.tif]

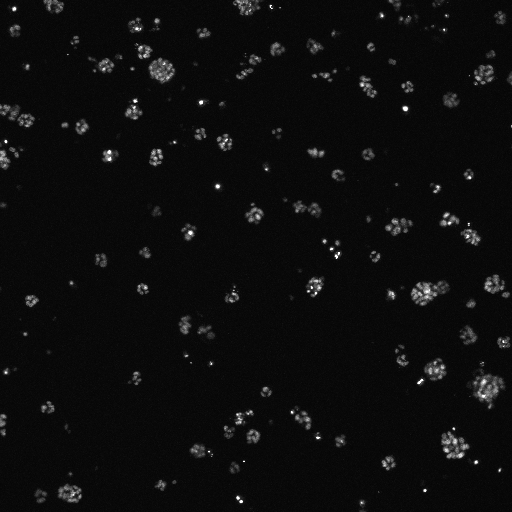

Supplement: Supplementary file 5 — Code EV1 [file MSB-13-955-s005.zip › DeathPro/example_images/150612_OC12_0h_2.1_MIPs/OC12_0h__W0004__P0002_channel0.tif]

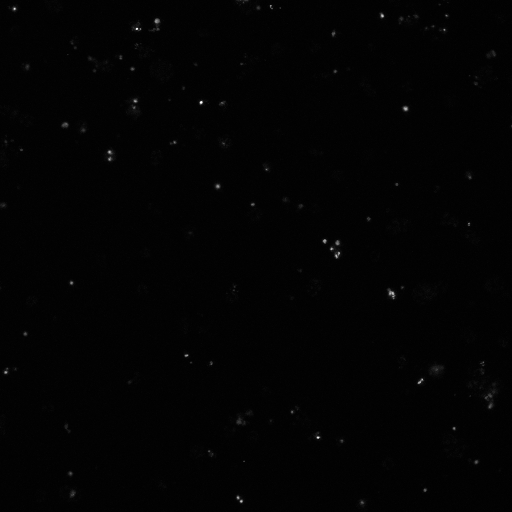

Supplement: Supplementary file 5 — Code EV1 [file MSB-13-955-s005.zip › DeathPro/example_images/150612_OC12_0h_2.1_MIPs/OC12_0h__W0004__P0002_channel1.tif]

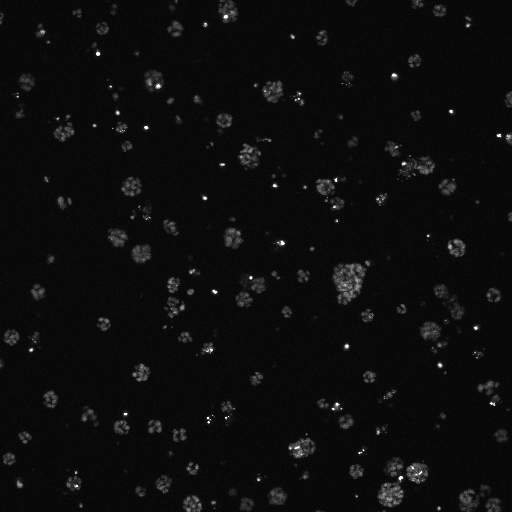

Supplement: Supplementary file 5 — Code EV1 [file MSB-13-955-s005.zip › DeathPro/example_images/150612_OC12_0h_2.1_MIPs/OC12_0h__W0005__P0001_channel0.tif]

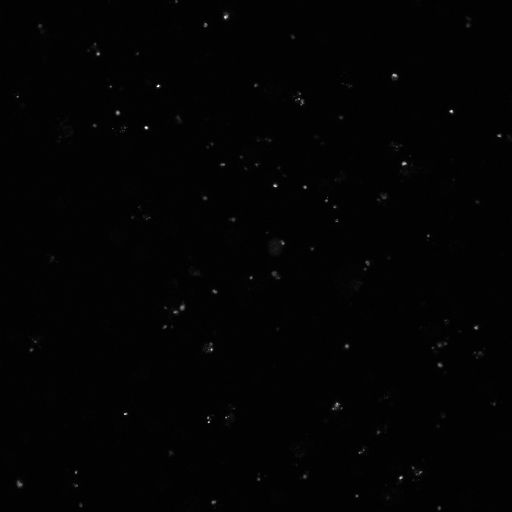

Supplement: Supplementary file 5 — Code EV1 [file MSB-13-955-s005.zip › DeathPro/example_images/150612_OC12_0h_2.1_MIPs/OC12_0h__W0005__P0001_channel1.tif]

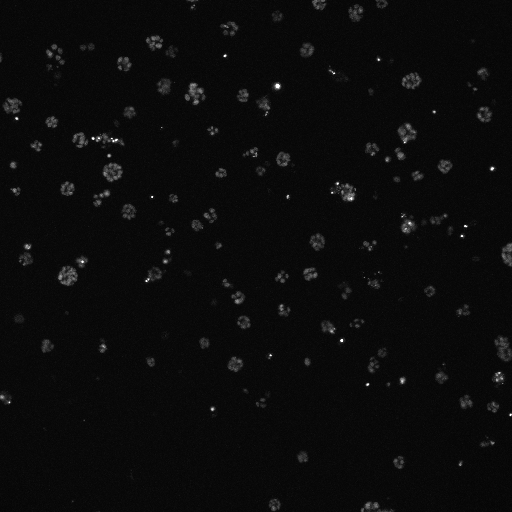

Supplement: Supplementary file 5 — Code EV1 [file MSB-13-955-s005.zip › DeathPro/example_images/150612_OC12_0h_2.1_MIPs/OC12_0h__W0005__P0002_channel0.tif]

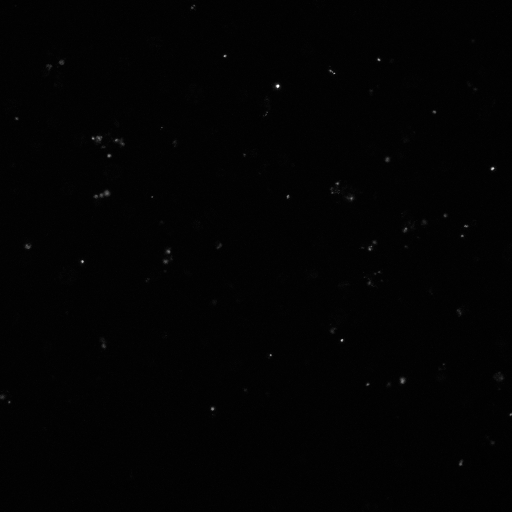

Supplement: Supplementary file 5 — Code EV1 [file MSB-13-955-s005.zip › DeathPro/example_images/150612_OC12_0h_2.1_MIPs/OC12_0h__W0005__P0002_channel1.tif]

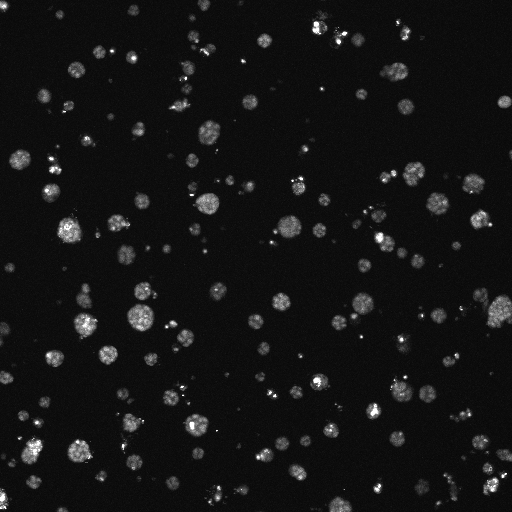

Supplement: Supplementary file 5 — Code EV1 [file MSB-13-955-s005.zip › DeathPro/example_images/150612_OC12_0h_2.1_MIPs/OC12_0h__W0006__P0001_channel0.tif]

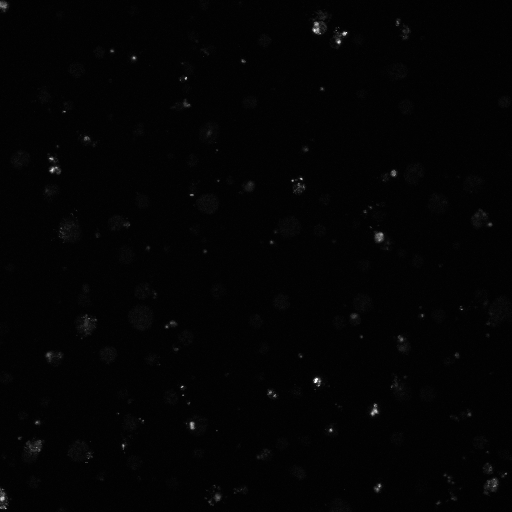

Supplement: Supplementary file 5 — Code EV1 [file MSB-13-955-s005.zip › DeathPro/example_images/150612_OC12_0h_2.1_MIPs/OC12_0h__W0006__P0001_channel1.tif]

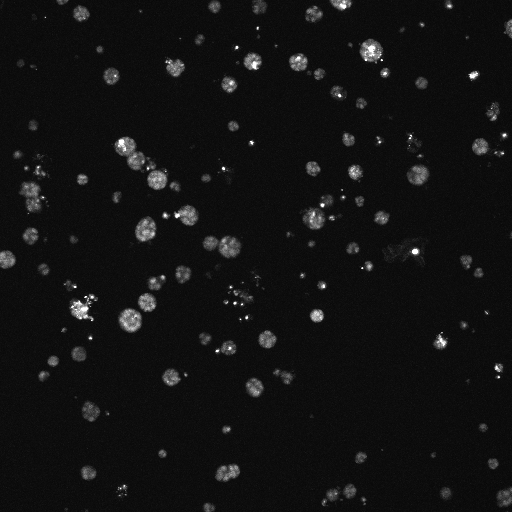

Supplement: Supplementary file 5 — Code EV1 [file MSB-13-955-s005.zip › DeathPro/example_images/150612_OC12_0h_2.1_MIPs/OC12_0h__W0006__P0002_channel0.tif]

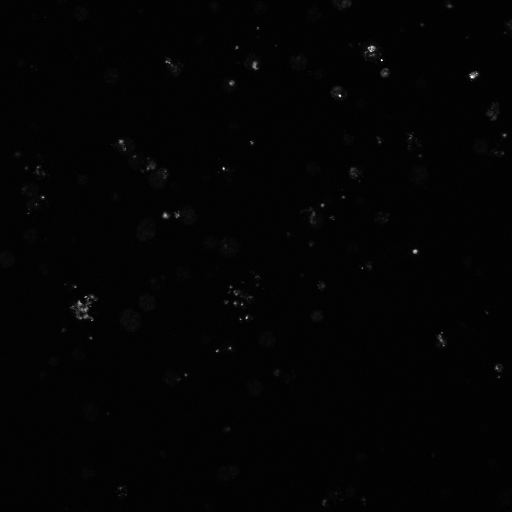

Supplement: Supplementary file 5 — Code EV1 [file MSB-13-955-s005.zip › DeathPro/example_images/150612_OC12_0h_2.1_MIPs/OC12_0h__W0006__P0002_channel1.tif]

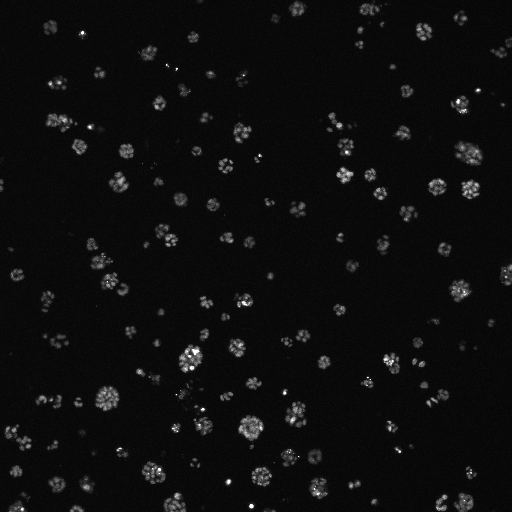

Supplement: Supplementary file 5 — Code EV1 [file MSB-13-955-s005.zip › DeathPro/example_images/150612_OC12_0h_2.1_MIPs/OC12_0h__W0007__P0001_channel0.tif]

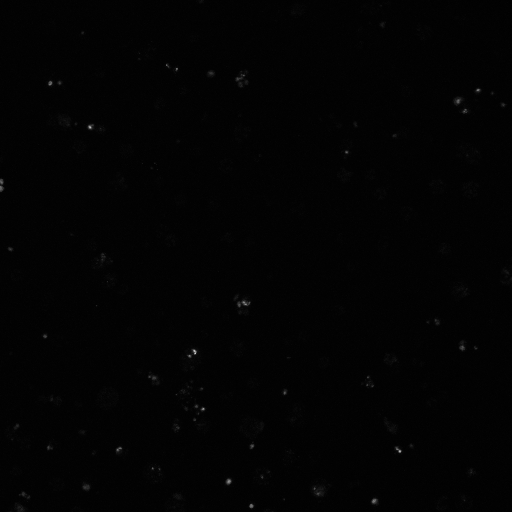

Supplement: Supplementary file 5 — Code EV1 [file MSB-13-955-s005.zip › DeathPro/example_images/150612_OC12_0h_2.1_MIPs/OC12_0h__W0007__P0001_channel1.tif]

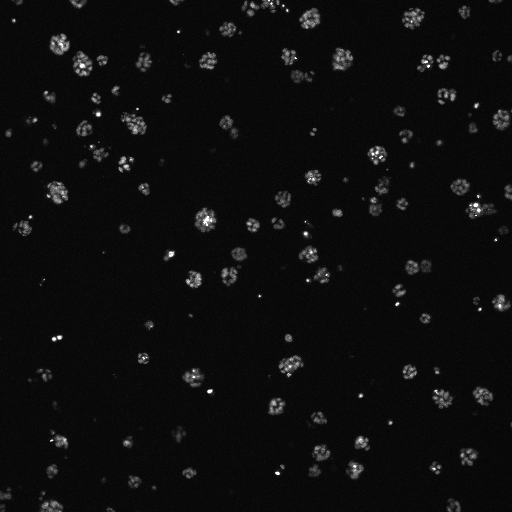

Supplement: Supplementary file 5 — Code EV1 [file MSB-13-955-s005.zip › DeathPro/example_images/150612_OC12_0h_2.1_MIPs/OC12_0h__W0007__P0002_channel0.tif]

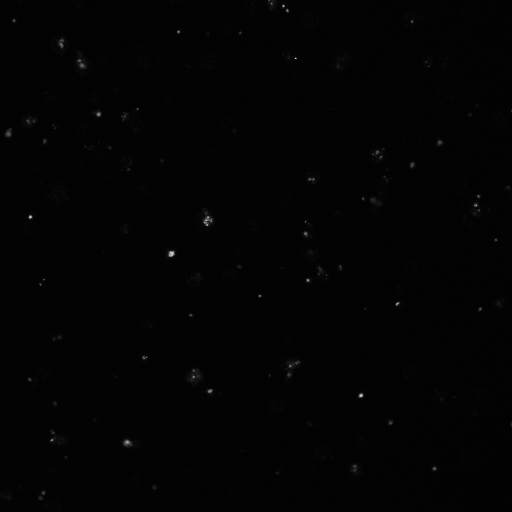

Supplement: Supplementary file 5 — Code EV1 [file MSB-13-955-s005.zip › DeathPro/example_images/150612_OC12_0h_2.1_MIPs/OC12_0h__W0007__P0002_channel1.tif]

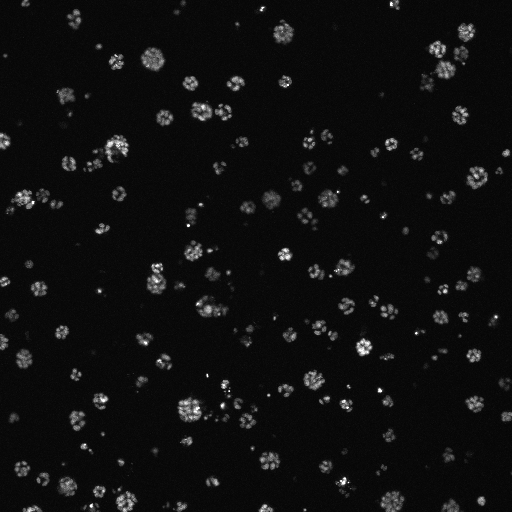

Supplement: Supplementary file 5 — Code EV1 [file MSB-13-955-s005.zip › DeathPro/example_images/150612_OC12_0h_2.1_MIPs/OC12_0h__W0008__P0001_channel0.tif]

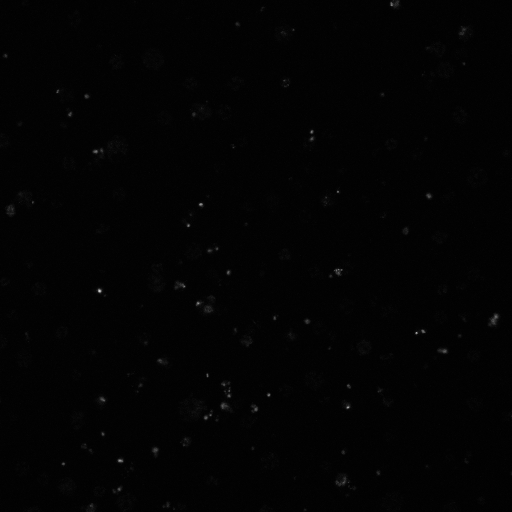

Supplement: Supplementary file 5 — Code EV1 [file MSB-13-955-s005.zip › DeathPro/example_images/150612_OC12_0h_2.1_MIPs/OC12_0h__W0008__P0001_channel1.tif]

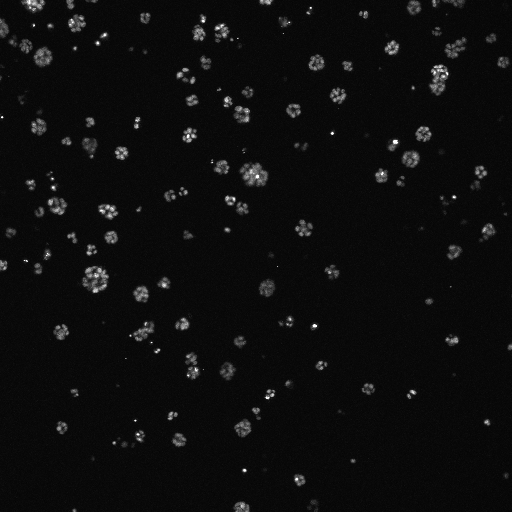

Supplement: Supplementary file 5 — Code EV1 [file MSB-13-955-s005.zip › DeathPro/example_images/150612_OC12_0h_2.1_MIPs/OC12_0h__W0008__P0002_channel0.tif]

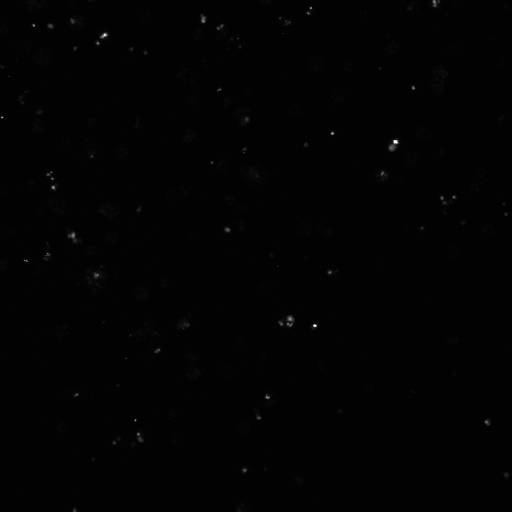

Supplement: Supplementary file 5 — Code EV1 [file MSB-13-955-s005.zip › DeathPro/example_images/150612_OC12_0h_2.1_MIPs/OC12_0h__W0008__P0002_channel1.tif]

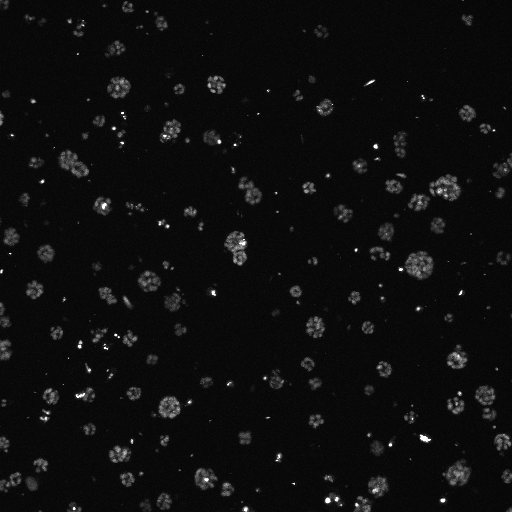

Supplement: Supplementary file 5 — Code EV1 [file MSB-13-955-s005.zip › DeathPro/example_images/150612_OC12_0h_2.1_MIPs/OC12_0h__W0009__P0001_channel0.tif]

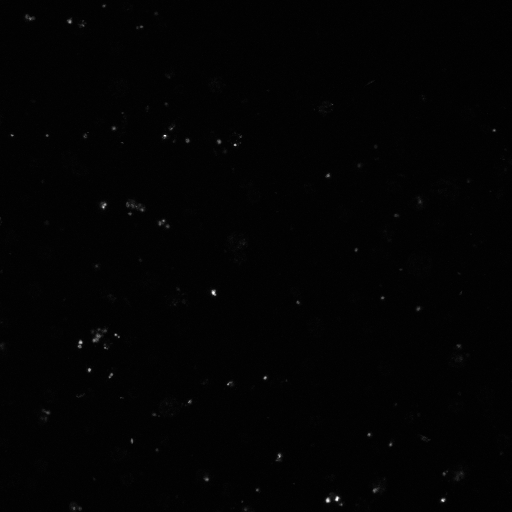

Supplement: Supplementary file 5 — Code EV1 [file MSB-13-955-s005.zip › DeathPro/example_images/150612_OC12_0h_2.1_MIPs/OC12_0h__W0009__P0001_channel1.tif]

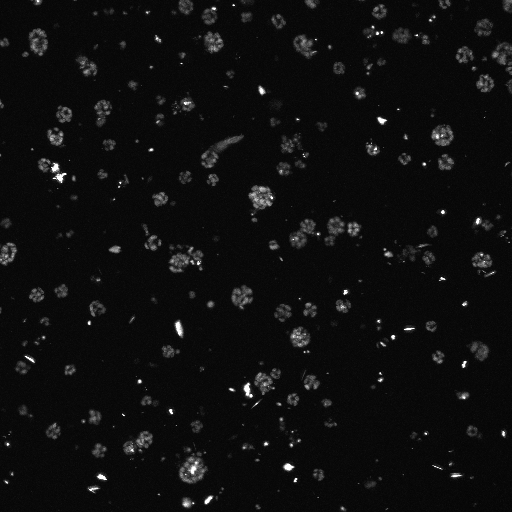

Supplement: Supplementary file 5 — Code EV1 [file MSB-13-955-s005.zip › DeathPro/example_images/150612_OC12_0h_2.1_MIPs/OC12_0h__W0009__P0002_channel0.tif]

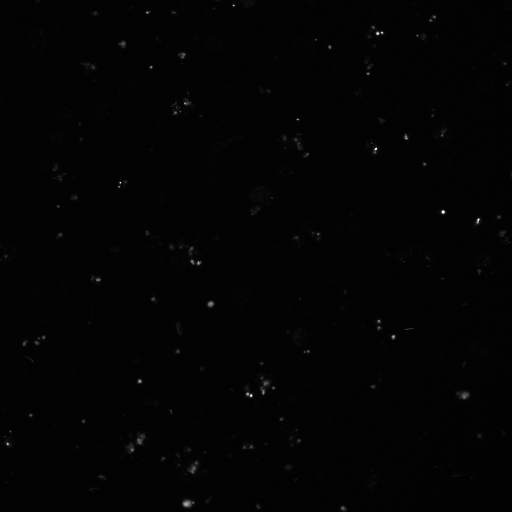

Supplement: Supplementary file 5 — Code EV1 [file MSB-13-955-s005.zip › DeathPro/example_images/150612_OC12_0h_2.1_MIPs/OC12_0h__W0009__P0002_channel1.tif]

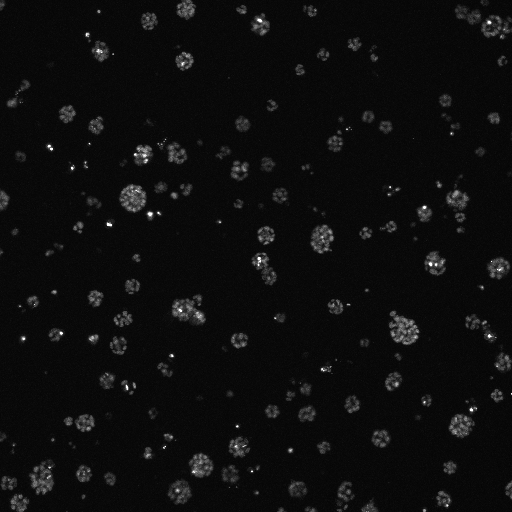

Supplement: Supplementary file 5 — Code EV1 [file MSB-13-955-s005.zip › DeathPro/example_images/150612_OC12_0h_2.1_MIPs/OC12_0h__W0010__P0001_channel0.tif]

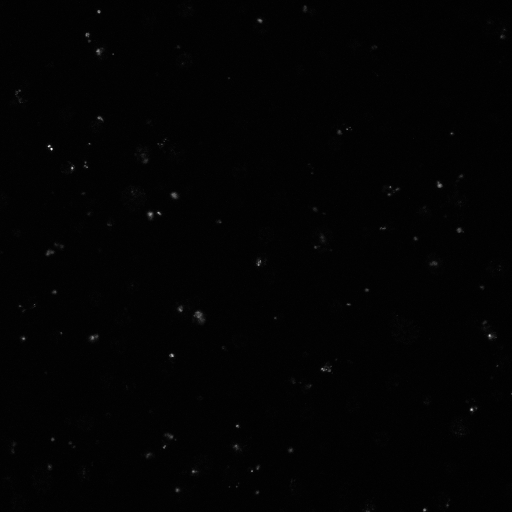

Supplement: Supplementary file 5 — Code EV1 [file MSB-13-955-s005.zip › DeathPro/example_images/150612_OC12_0h_2.1_MIPs/OC12_0h__W0010__P0001_channel1.tif]

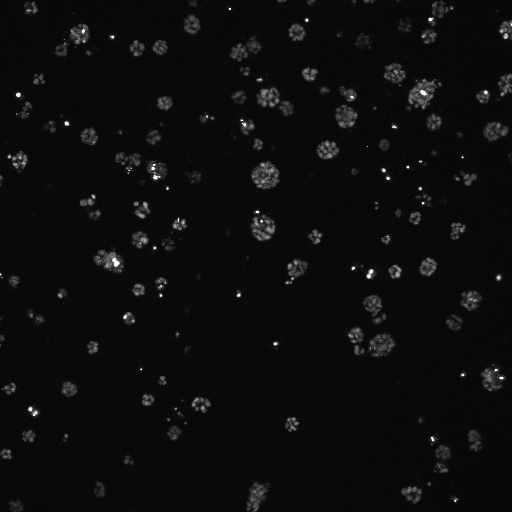

Supplement: Supplementary file 5 — Code EV1 [file MSB-13-955-s005.zip › DeathPro/example_images/150612_OC12_0h_2.1_MIPs/OC12_0h__W0010__P0002_channel0.tif]

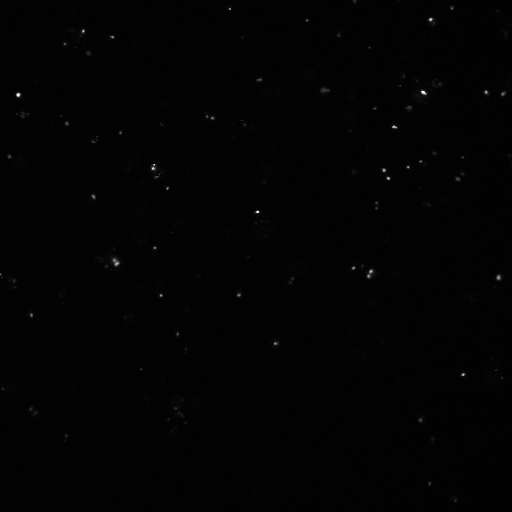

Supplement: Supplementary file 5 — Code EV1 [file MSB-13-955-s005.zip › DeathPro/example_images/150612_OC12_0h_2.1_MIPs/OC12_0h__W0010__P0002_channel1.tif]

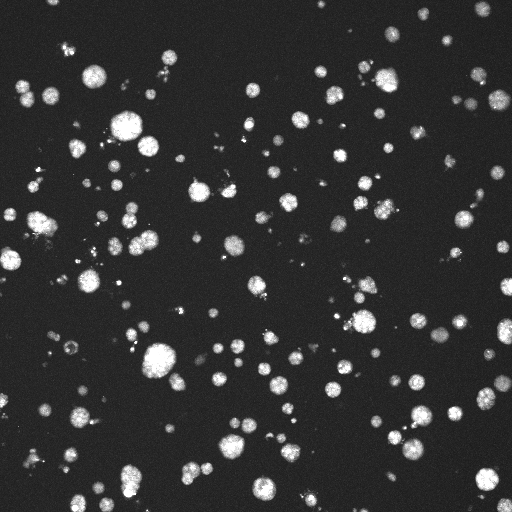

Supplement: Supplementary file 5 — Code EV1 [file MSB-13-955-s005.zip › DeathPro/example_images/150612_OC12_0h_2.1_MIPs/OC12_0h__W0011__P0001_channel0.tif]

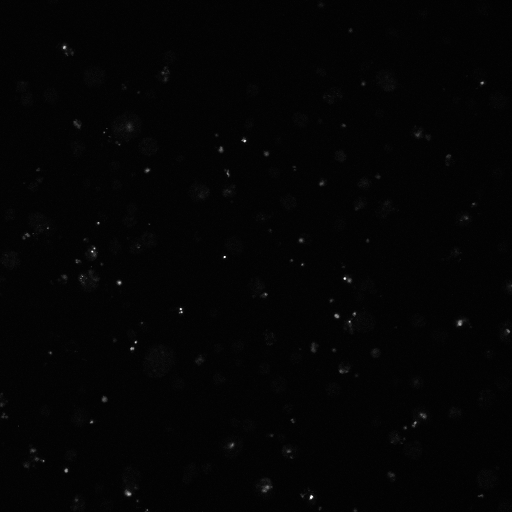

Supplement: Supplementary file 5 — Code EV1 [file MSB-13-955-s005.zip › DeathPro/example_images/150612_OC12_0h_2.1_MIPs/OC12_0h__W0011__P0001_channel1.tif]

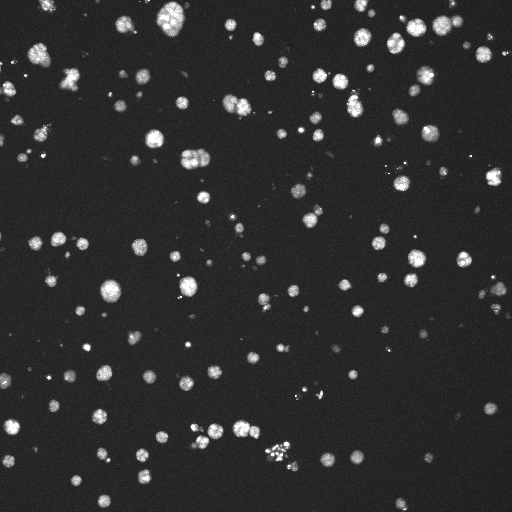

Supplement: Supplementary file 5 — Code EV1 [file MSB-13-955-s005.zip › DeathPro/example_images/150612_OC12_0h_2.1_MIPs/OC12_0h__W0011__P0002_channel0.tif]

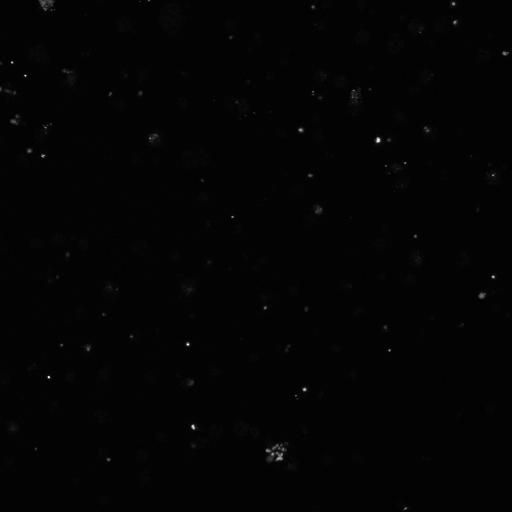

Supplement: Supplementary file 5 — Code EV1 [file MSB-13-955-s005.zip › DeathPro/example_images/150612_OC12_0h_2.1_MIPs/OC12_0h__W0011__P0002_channel1.tif]

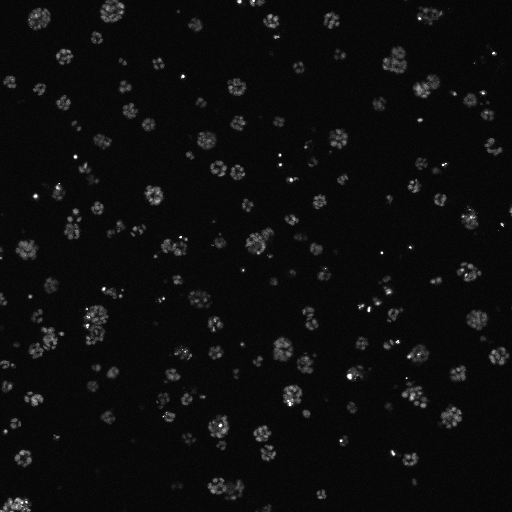

Supplement: Supplementary file 5 — Code EV1 [file MSB-13-955-s005.zip › DeathPro/example_images/150612_OC12_0h_2.1_MIPs/OC12_0h__W0012__P0001_channel0.tif]

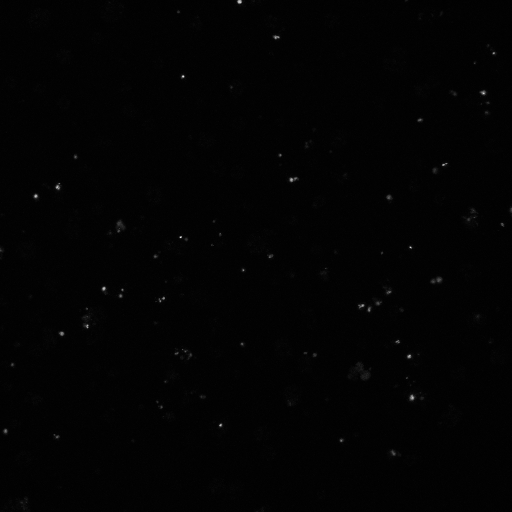

Supplement: Supplementary file 5 — Code EV1 [file MSB-13-955-s005.zip › DeathPro/example_images/150612_OC12_0h_2.1_MIPs/OC12_0h__W0012__P0001_channel1.tif]

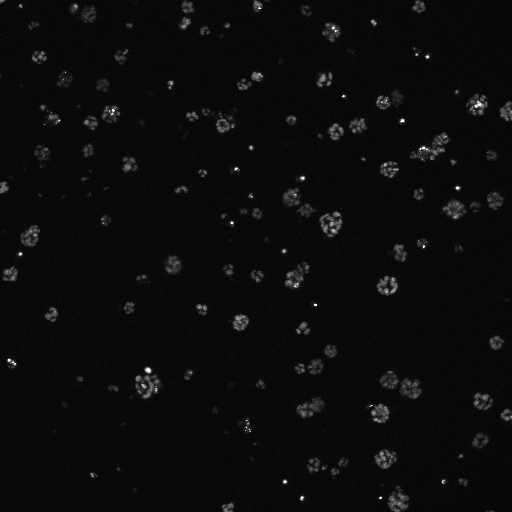

Supplement: Supplementary file 5 — Code EV1 [file MSB-13-955-s005.zip › DeathPro/example_images/150612_OC12_0h_2.1_MIPs/OC12_0h__W0012__P0002_channel0.tif]

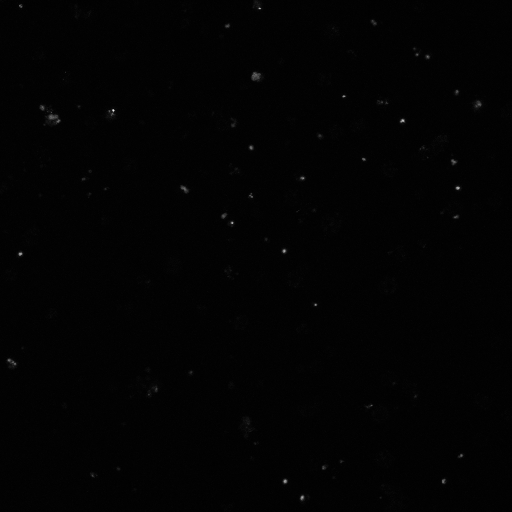

Supplement: Supplementary file 5 — Code EV1 [file MSB-13-955-s005.zip › DeathPro/example_images/150612_OC12_0h_2.1_MIPs/OC12_0h__W0012__P0002_channel1.tif]

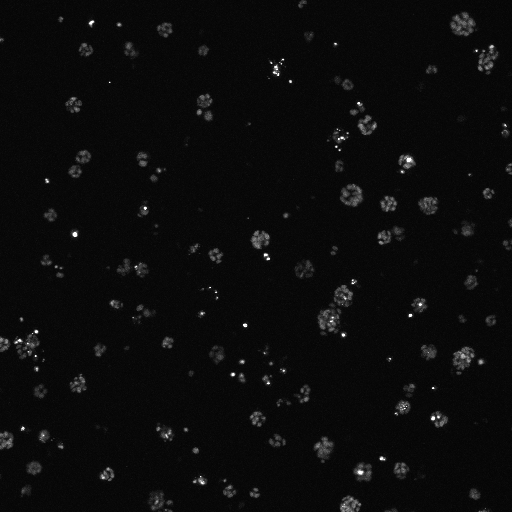

Supplement: Supplementary file 5 — Code EV1 [file MSB-13-955-s005.zip › DeathPro/example_images/150612_OC12_0h_2.1_MIPs/OC12_0h__W0013__P0001_channel0.tif]

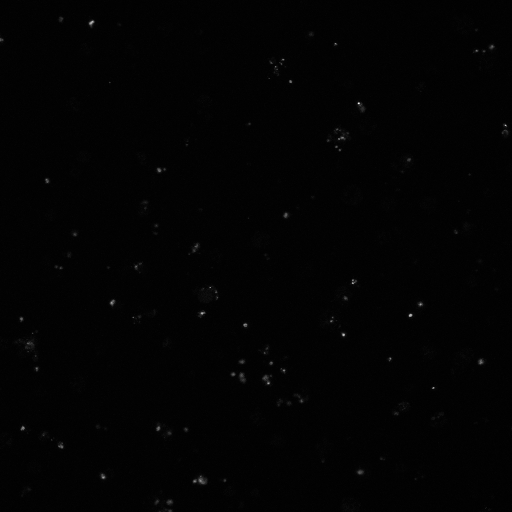

Supplement: Supplementary file 5 — Code EV1 [file MSB-13-955-s005.zip › DeathPro/example_images/150612_OC12_0h_2.1_MIPs/OC12_0h__W0013__P0001_channel1.tif]

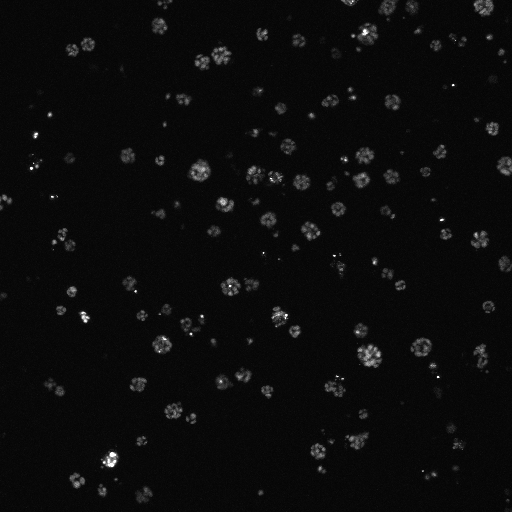

Supplement: Supplementary file 5 — Code EV1 [file MSB-13-955-s005.zip › DeathPro/example_images/150612_OC12_0h_2.1_MIPs/OC12_0h__W0013__P0002_channel0.tif]

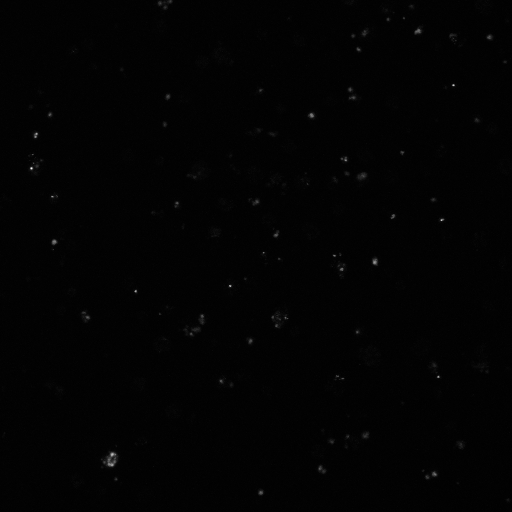

Supplement: Supplementary file 5 — Code EV1 [file MSB-13-955-s005.zip › DeathPro/example_images/150612_OC12_0h_2.1_MIPs/OC12_0h__W0013__P0002_channel1.tif]

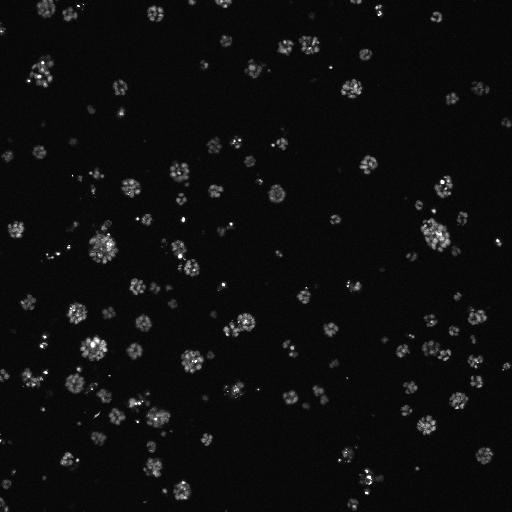

Supplement: Supplementary file 5 — Code EV1 [file MSB-13-955-s005.zip › DeathPro/example_images/150612_OC12_0h_2.1_MIPs/OC12_0h__W0014__P0001_channel0.tif]

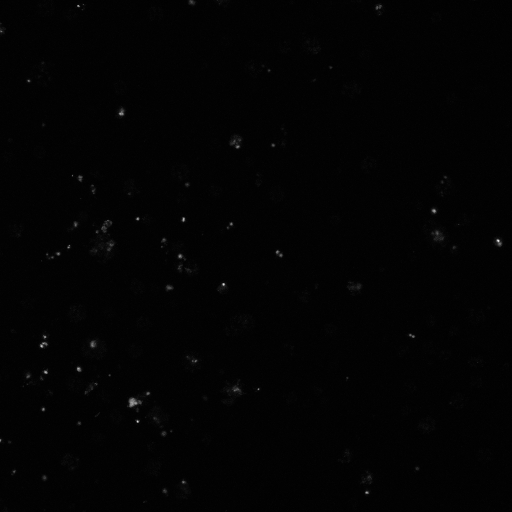

Supplement: Supplementary file 5 — Code EV1 [file MSB-13-955-s005.zip › DeathPro/example_images/150612_OC12_0h_2.1_MIPs/OC12_0h__W0014__P0001_channel1.tif]

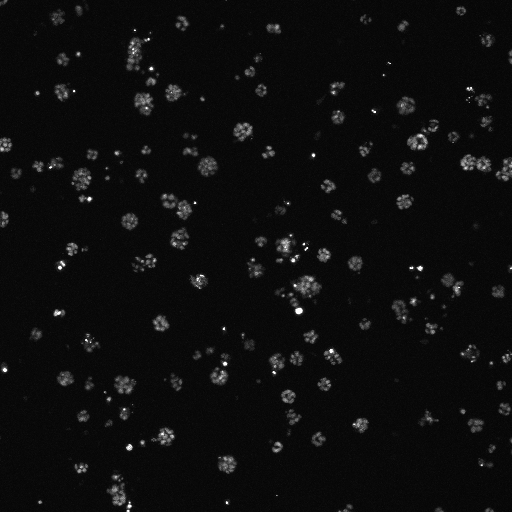

Supplement: Supplementary file 5 — Code EV1 [file MSB-13-955-s005.zip › DeathPro/example_images/150612_OC12_0h_2.1_MIPs/OC12_0h__W0014__P0002_channel0.tif]

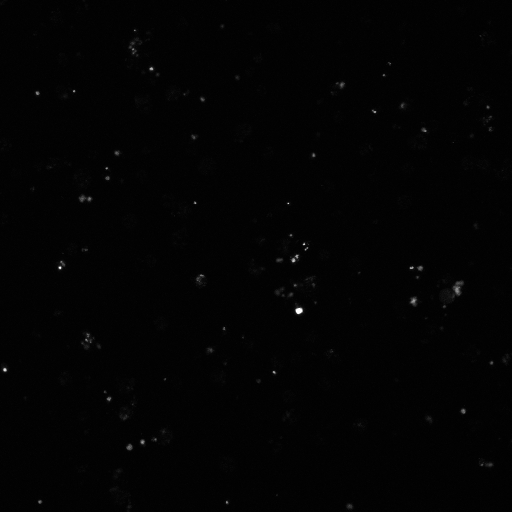

Supplement: Supplementary file 5 — Code EV1 [file MSB-13-955-s005.zip › DeathPro/example_images/150612_OC12_0h_2.1_MIPs/OC12_0h__W0014__P0002_channel1.tif]

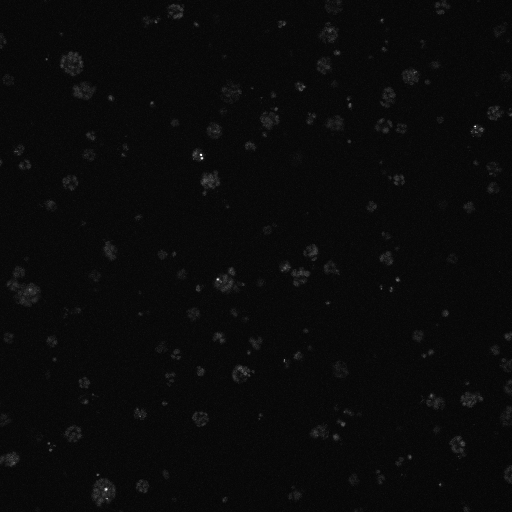

Supplement: Supplementary file 5 — Code EV1 [file MSB-13-955-s005.zip › DeathPro/example_images/150612_OC12_0h_2.1_MIPs/OC12_0h__W0015__P0001_channel0.tif]

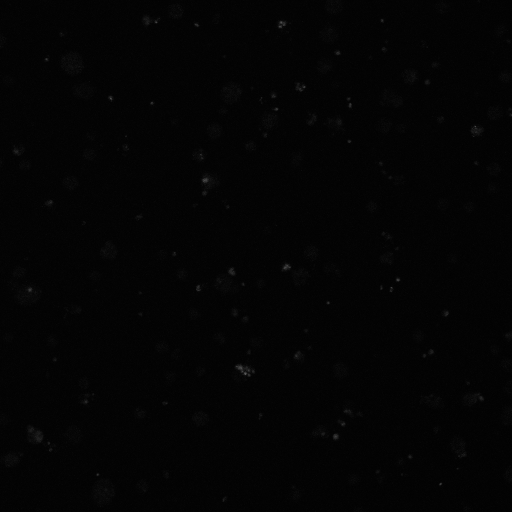

Supplement: Supplementary file 5 — Code EV1 [file MSB-13-955-s005.zip › DeathPro/example_images/150612_OC12_0h_2.1_MIPs/OC12_0h__W0015__P0001_channel1.tif]

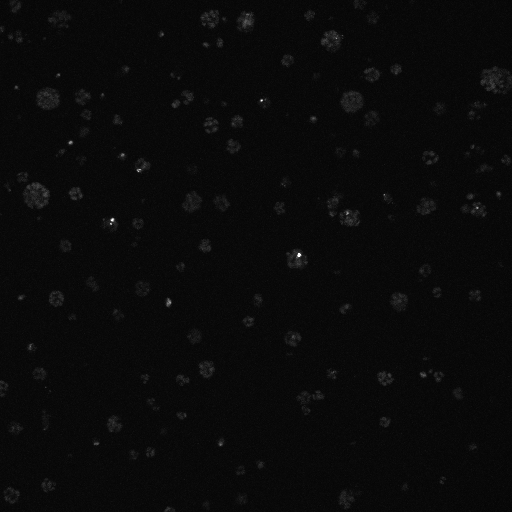

Supplement: Supplementary file 5 — Code EV1 [file MSB-13-955-s005.zip › DeathPro/example_images/150612_OC12_0h_2.1_MIPs/OC12_0h__W0015__P0002_channel0.tif]

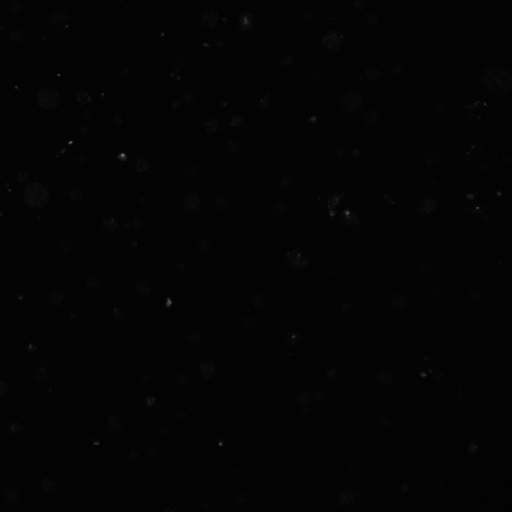

Supplement: Supplementary file 5 — Code EV1 [file MSB-13-955-s005.zip › DeathPro/example_images/150612_OC12_0h_2.1_MIPs/OC12_0h__W0015__P0002_channel1.tif]

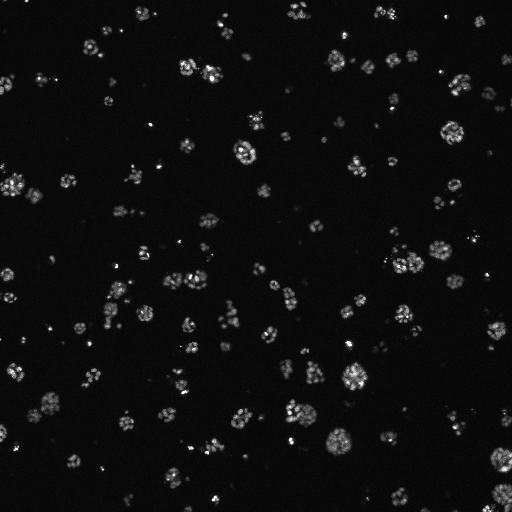

Supplement: Supplementary file 5 — Code EV1 [file MSB-13-955-s005.zip › DeathPro/example_images/150612_OC12_0h_2.1_MIPs/OC12_0h__W0016__P0001_channel0.tif]

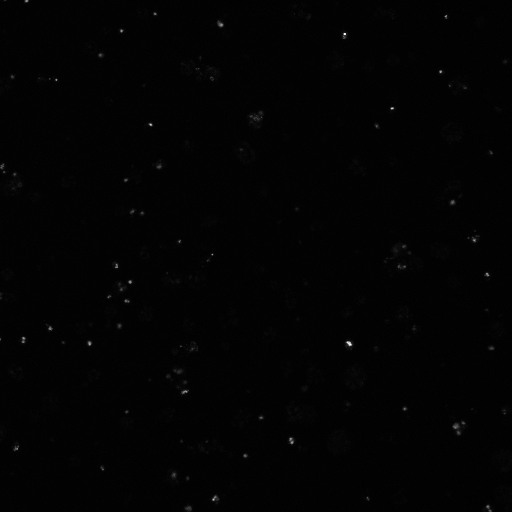

Supplement: Supplementary file 5 — Code EV1 [file MSB-13-955-s005.zip › DeathPro/example_images/150612_OC12_0h_2.1_MIPs/OC12_0h__W0016__P0001_channel1.tif]

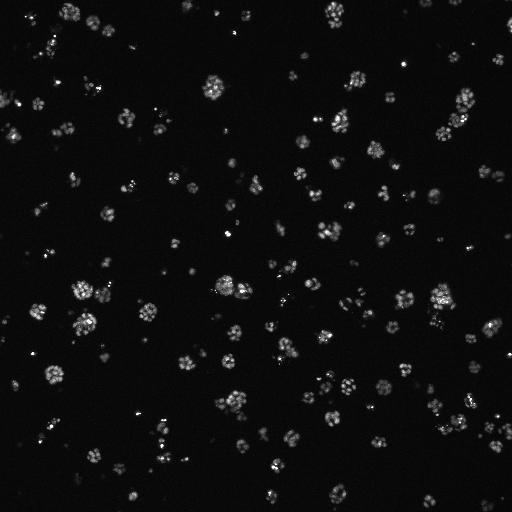

Supplement: Supplementary file 5 — Code EV1 [file MSB-13-955-s005.zip › DeathPro/example_images/150612_OC12_0h_2.1_MIPs/OC12_0h__W0016__P0002_channel0.tif]

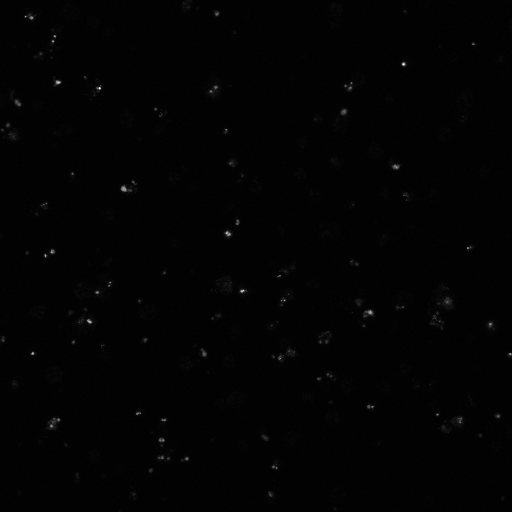

Supplement: Supplementary file 5 — Code EV1 [file MSB-13-955-s005.zip › DeathPro/example_images/150612_OC12_0h_2.1_MIPs/OC12_0h__W0016__P0002_channel1.tif]

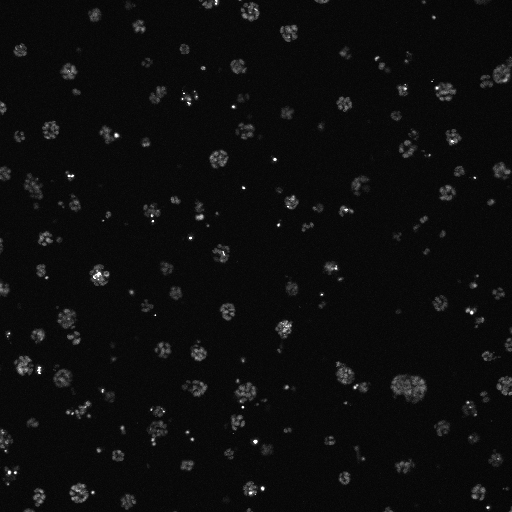

Supplement: Supplementary file 5 — Code EV1 [file MSB-13-955-s005.zip › DeathPro/example_images/150612_OC12_0h_2.1_MIPs/OC12_0h__W0017__P0001_channel0.tif]

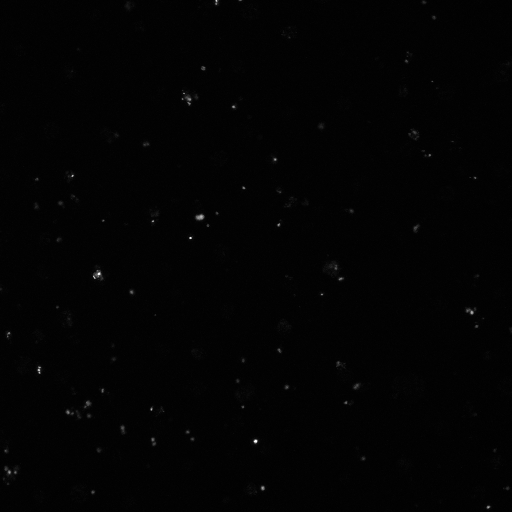

Supplement: Supplementary file 5 — Code EV1 [file MSB-13-955-s005.zip › DeathPro/example_images/150612_OC12_0h_2.1_MIPs/OC12_0h__W0017__P0001_channel1.tif]

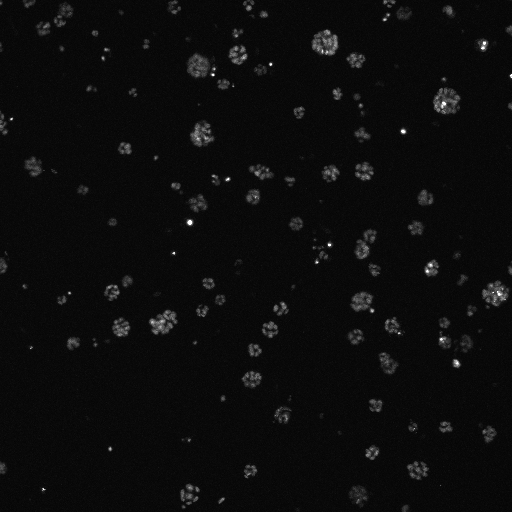

Supplement: Supplementary file 5 — Code EV1 [file MSB-13-955-s005.zip › DeathPro/example_images/150612_OC12_0h_2.1_MIPs/OC12_0h__W0017__P0002_channel0.tif]

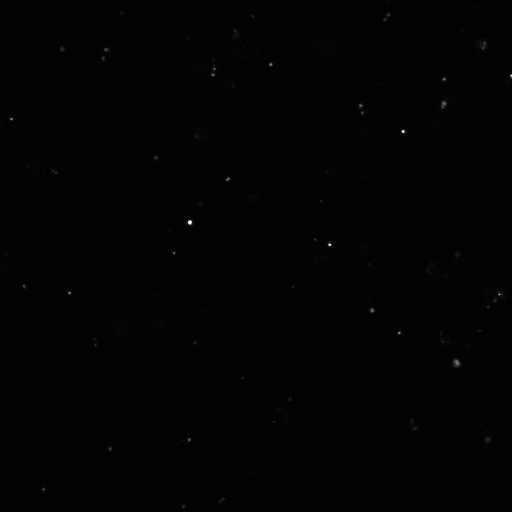

Supplement: Supplementary file 5 — Code EV1 [file MSB-13-955-s005.zip › DeathPro/example_images/150612_OC12_0h_2.1_MIPs/OC12_0h__W0017__P0002_channel1.tif]

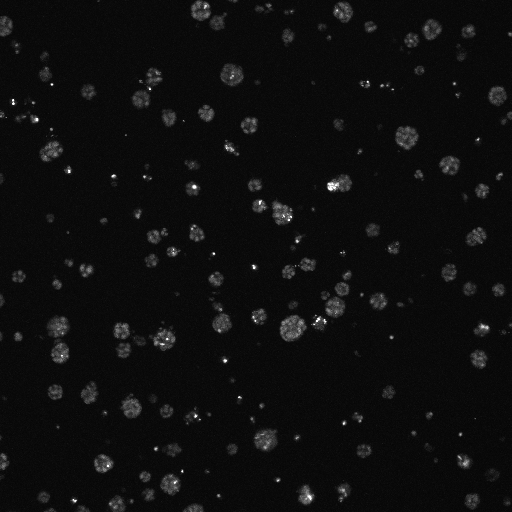

Supplement: Supplementary file 5 — Code EV1 [file MSB-13-955-s005.zip › DeathPro/example_images/150612_OC12_0h_2.1_MIPs/OC12_0h__W0018__P0001_channel0.tif]

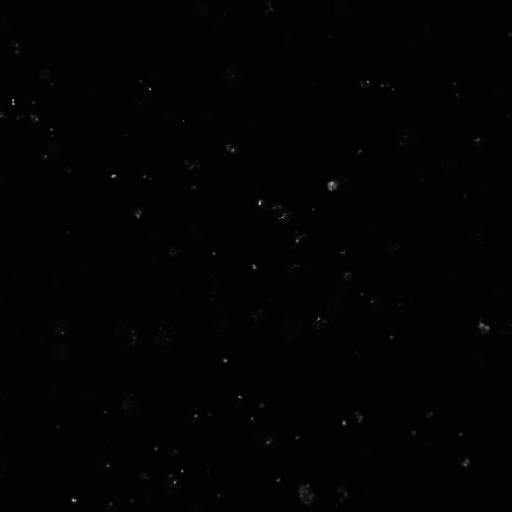

Supplement: Supplementary file 5 — Code EV1 [file MSB-13-955-s005.zip › DeathPro/example_images/150612_OC12_0h_2.1_MIPs/OC12_0h__W0018__P0001_channel1.tif]

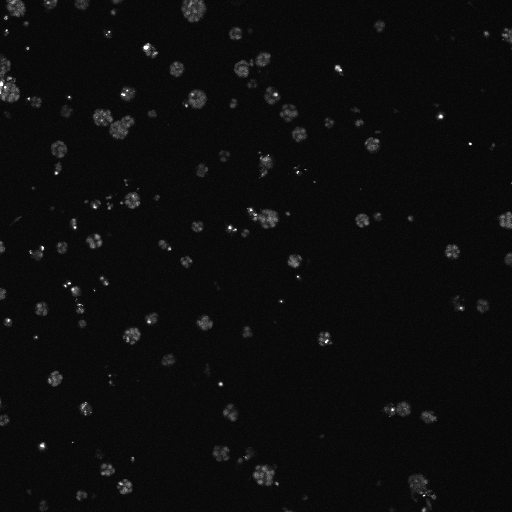

Supplement: Supplementary file 5 — Code EV1 [file MSB-13-955-s005.zip › DeathPro/example_images/150612_OC12_0h_2.1_MIPs/OC12_0h__W0018__P0002_channel0.tif]

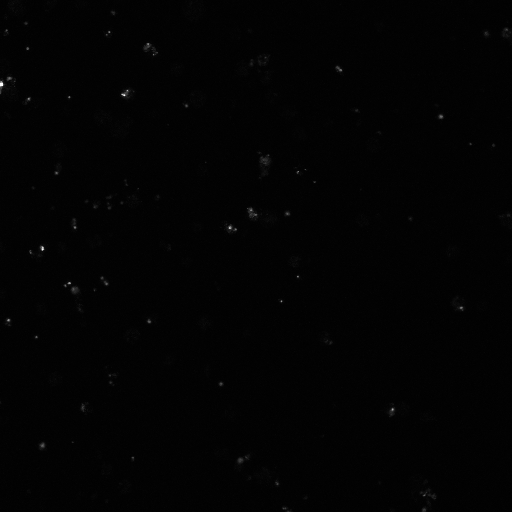

Supplement: Supplementary file 5 — Code EV1 [file MSB-13-955-s005.zip › DeathPro/example_images/150612_OC12_0h_2.1_MIPs/OC12_0h__W0018__P0002_channel1.tif]

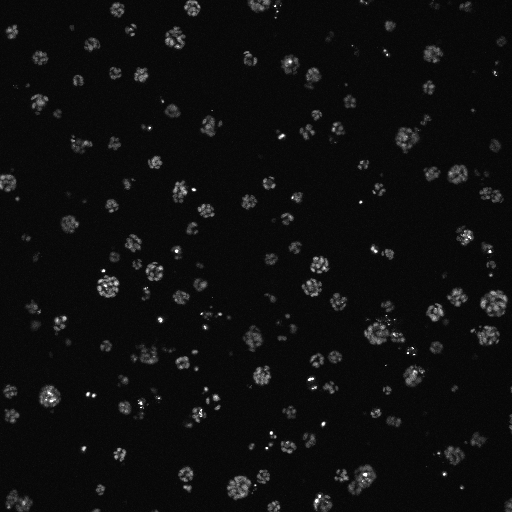

Supplement: Supplementary file 5 — Code EV1 [file MSB-13-955-s005.zip › DeathPro/example_images/150612_OC12_0h_2.1_MIPs/OC12_0h__W0019__P0001_channel0.tif]

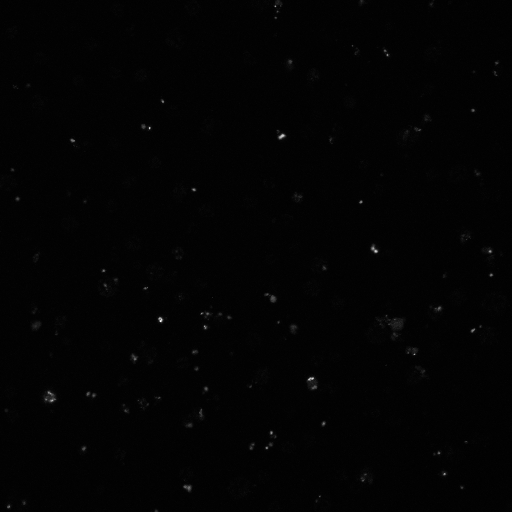

Supplement: Supplementary file 5 — Code EV1 [file MSB-13-955-s005.zip › DeathPro/example_images/150612_OC12_0h_2.1_MIPs/OC12_0h__W0019__P0001_channel1.tif]

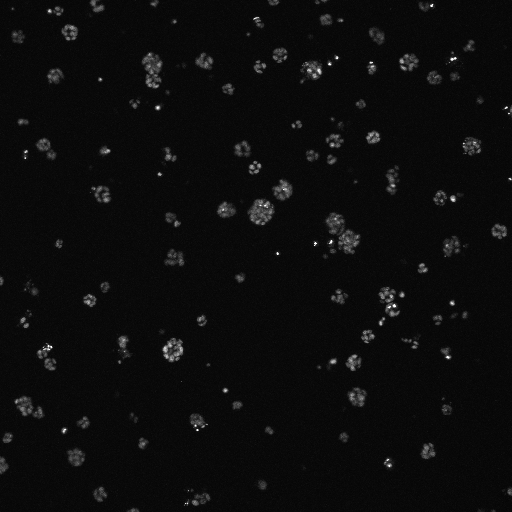

Supplement: Supplementary file 5 — Code EV1 [file MSB-13-955-s005.zip › DeathPro/example_images/150612_OC12_0h_2.1_MIPs/OC12_0h__W0019__P0002_channel0.tif]

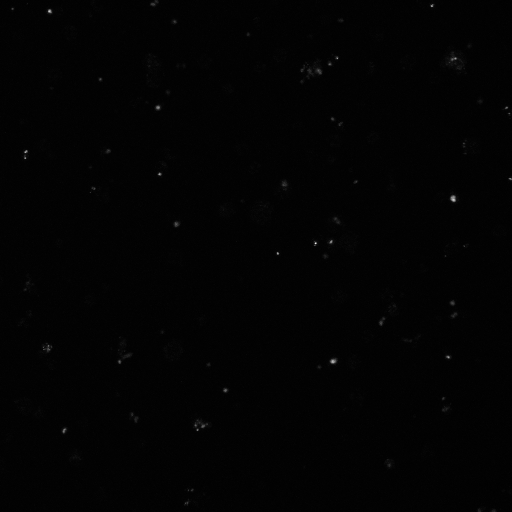

Supplement: Supplementary file 5 — Code EV1 [file MSB-13-955-s005.zip › DeathPro/example_images/150612_OC12_0h_2.1_MIPs/OC12_0h__W0019__P0002_channel1.tif]

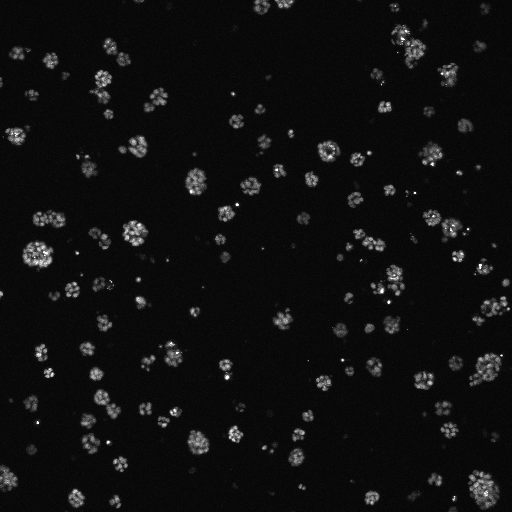

Supplement: Supplementary file 5 — Code EV1 [file MSB-13-955-s005.zip › DeathPro/example_images/150612_OC12_0h_2.1_MIPs/OC12_0h__W0020__P0001_channel0.tif]

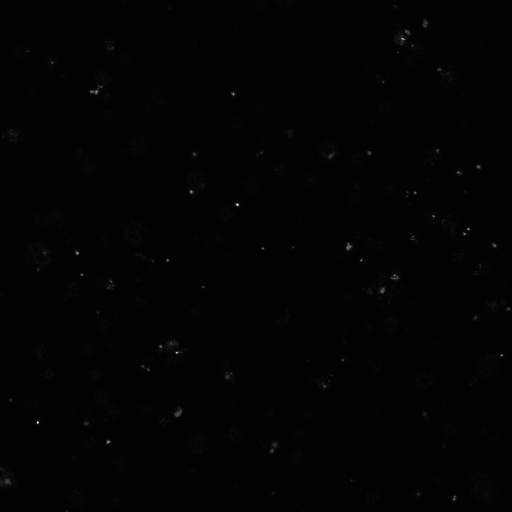

Supplement: Supplementary file 5 — Code EV1 [file MSB-13-955-s005.zip › DeathPro/example_images/150612_OC12_0h_2.1_MIPs/OC12_0h__W0020__P0001_channel1.tif]

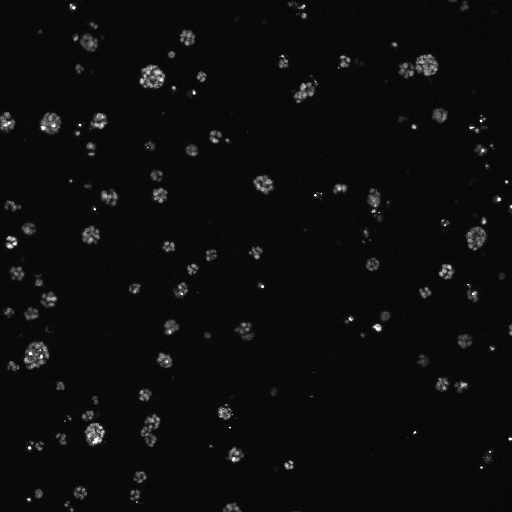

Supplement: Supplementary file 5 — Code EV1 [file MSB-13-955-s005.zip › DeathPro/example_images/150612_OC12_0h_2.1_MIPs/OC12_0h__W0020__P0002_channel0.tif]

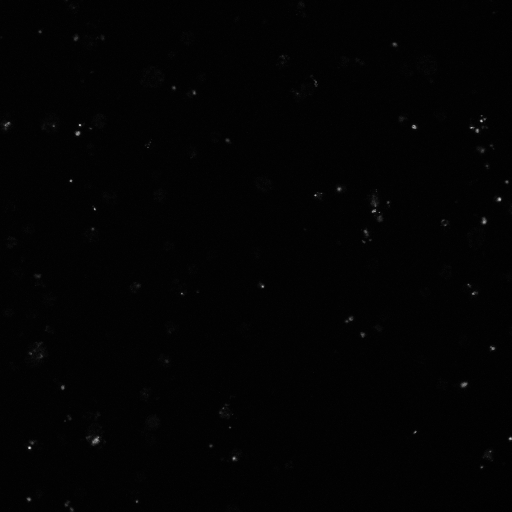

Supplement: Supplementary file 5 — Code EV1 [file MSB-13-955-s005.zip › DeathPro/example_images/150612_OC12_0h_2.1_MIPs/OC12_0h__W0020__P0002_channel1.tif]

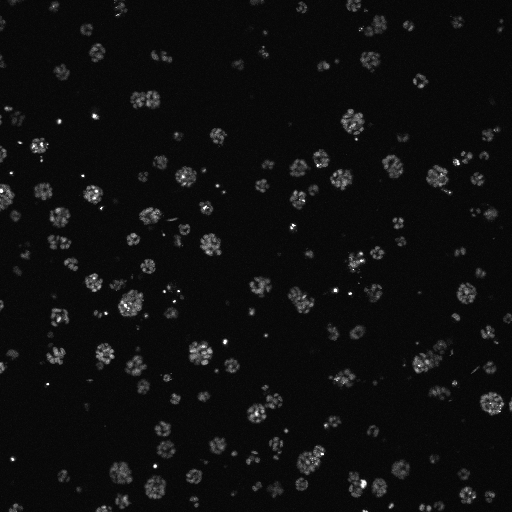

Supplement: Supplementary file 5 — Code EV1 [file MSB-13-955-s005.zip › DeathPro/example_images/150612_OC12_0h_2.1_MIPs/OC12_0h__W0021__P0001_channel0.tif]

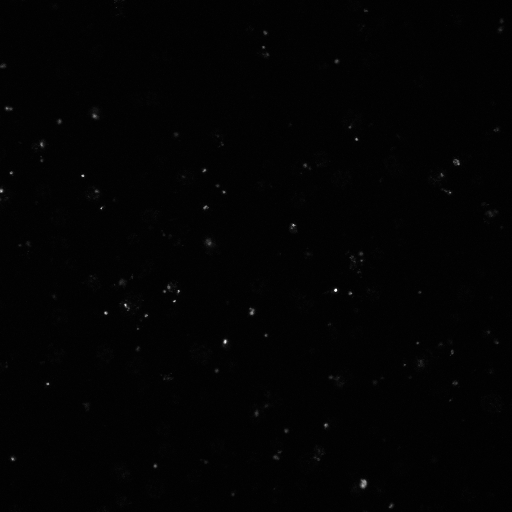

Supplement: Supplementary file 5 — Code EV1 [file MSB-13-955-s005.zip › DeathPro/example_images/150612_OC12_0h_2.1_MIPs/OC12_0h__W0021__P0001_channel1.tif]

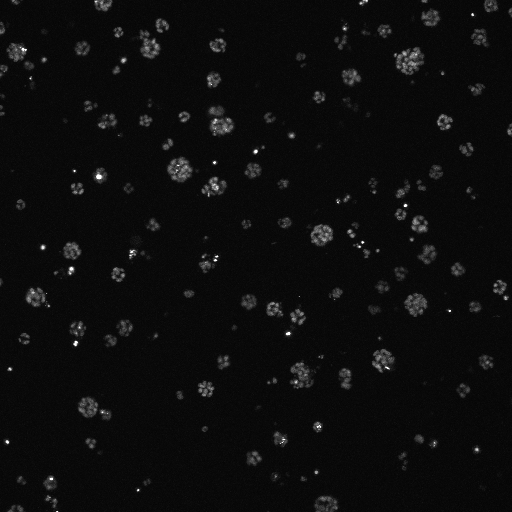

Supplement: Supplementary file 5 — Code EV1 [file MSB-13-955-s005.zip › DeathPro/example_images/150612_OC12_0h_2.1_MIPs/OC12_0h__W0021__P0002_channel0.tif]

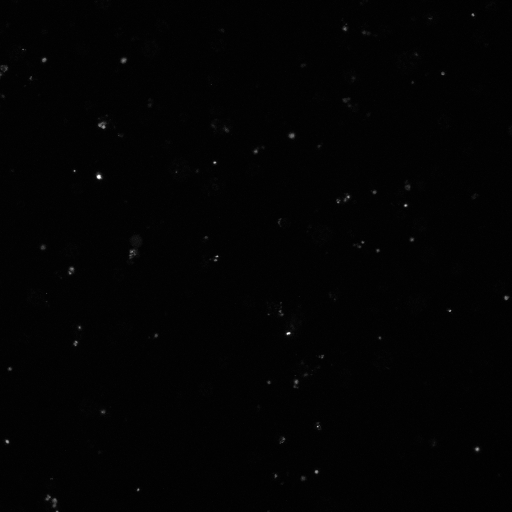

Supplement: Supplementary file 5 — Code EV1 [file MSB-13-955-s005.zip › DeathPro/example_images/150612_OC12_0h_2.1_MIPs/OC12_0h__W0021__P0002_channel1.tif]

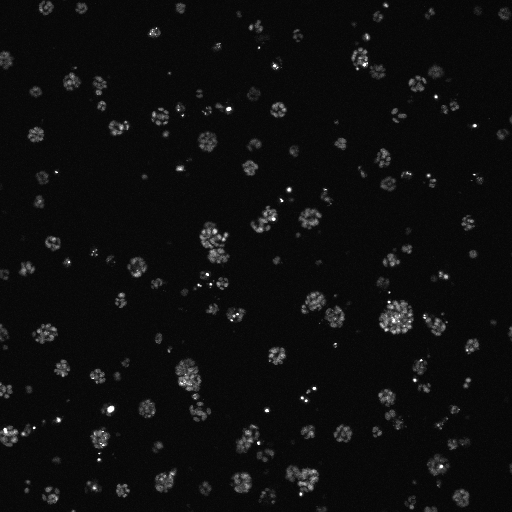

Supplement: Supplementary file 5 — Code EV1 [file MSB-13-955-s005.zip › DeathPro/example_images/150612_OC12_0h_2.1_MIPs/OC12_0h__W0022__P0001_channel0.tif]

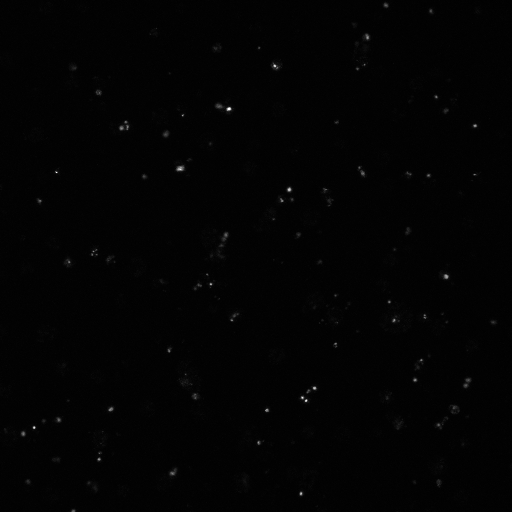

Supplement: Supplementary file 5 — Code EV1 [file MSB-13-955-s005.zip › DeathPro/example_images/150612_OC12_0h_2.1_MIPs/OC12_0h__W0022__P0001_channel1.tif]

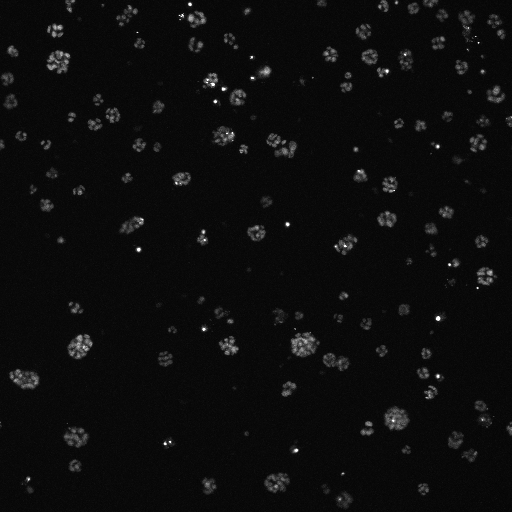

Supplement: Supplementary file 5 — Code EV1 [file MSB-13-955-s005.zip › DeathPro/example_images/150612_OC12_0h_2.1_MIPs/OC12_0h__W0022__P0002_channel0.tif]

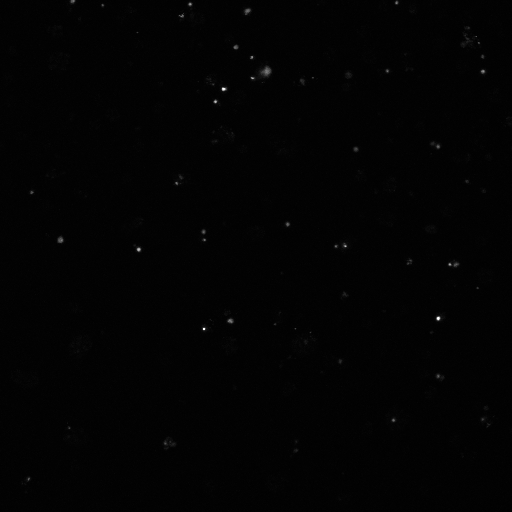

Supplement: Supplementary file 5 — Code EV1 [file MSB-13-955-s005.zip › DeathPro/example_images/150612_OC12_0h_2.1_MIPs/OC12_0h__W0022__P0002_channel1.tif]

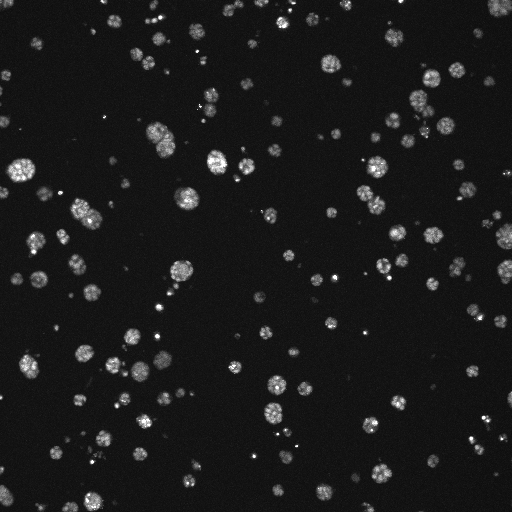

Supplement: Supplementary file 5 — Code EV1 [file MSB-13-955-s005.zip › DeathPro/example_images/150612_OC12_0h_2.1_MIPs/OC12_0h__W0023__P0001_channel0.tif]

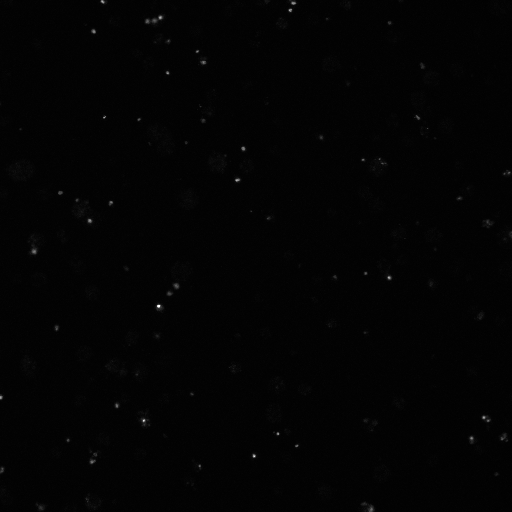

Supplement: Supplementary file 5 — Code EV1 [file MSB-13-955-s005.zip › DeathPro/example_images/150612_OC12_0h_2.1_MIPs/OC12_0h__W0023__P0001_channel1.tif]

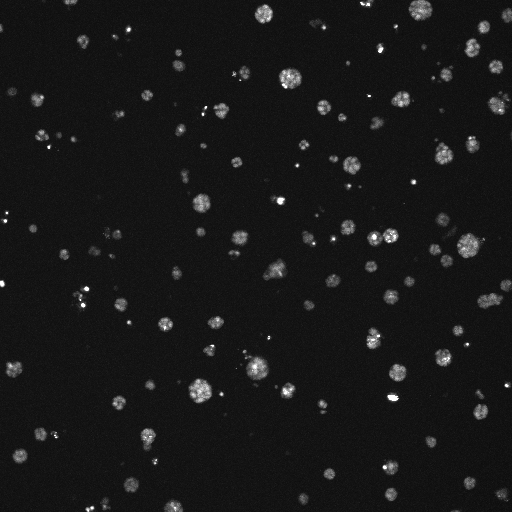

Supplement: Supplementary file 5 — Code EV1 [file MSB-13-955-s005.zip › DeathPro/example_images/150612_OC12_0h_2.1_MIPs/OC12_0h__W0023__P0002_channel0.tif]

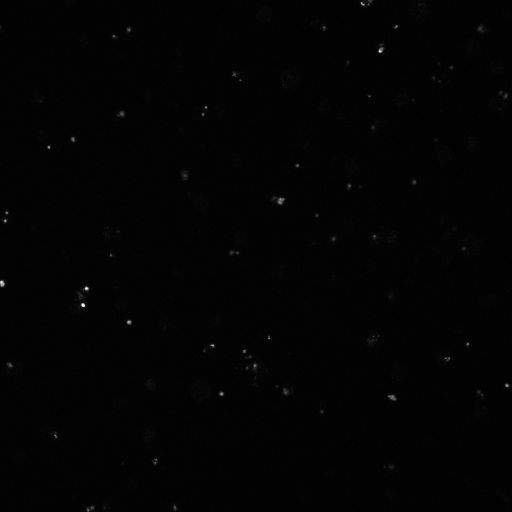

Supplement: Supplementary file 5 — Code EV1 [file MSB-13-955-s005.zip › DeathPro/example_images/150612_OC12_0h_2.1_MIPs/OC12_0h__W0023__P0002_channel1.tif]

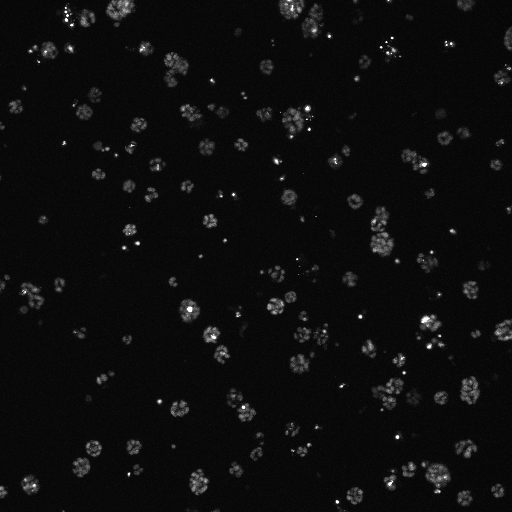

Supplement: Supplementary file 5 — Code EV1 [file MSB-13-955-s005.zip › DeathPro/example_images/150612_OC12_0h_2.1_MIPs/OC12_0h__W0024__P0001_channel0.tif]

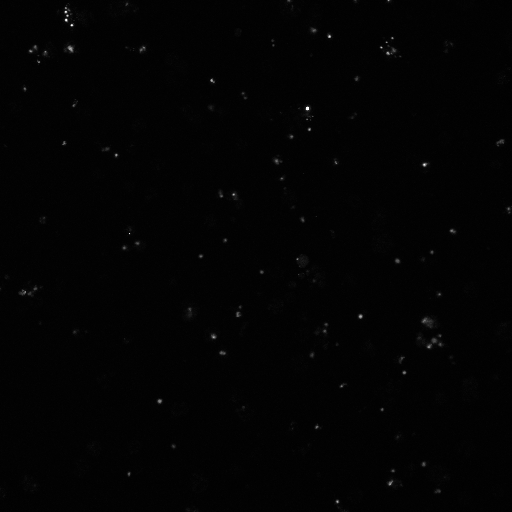

Supplement: Supplementary file 5 — Code EV1 [file MSB-13-955-s005.zip › DeathPro/example_images/150612_OC12_0h_2.1_MIPs/OC12_0h__W0024__P0001_channel1.tif]

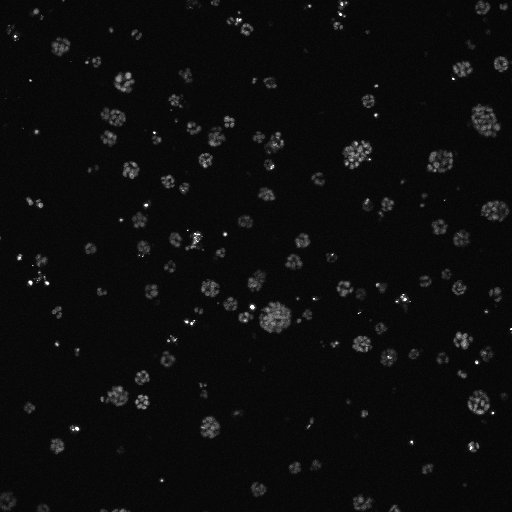

Supplement: Supplementary file 5 — Code EV1 [file MSB-13-955-s005.zip › DeathPro/example_images/150612_OC12_0h_2.1_MIPs/OC12_0h__W0024__P0002_channel0.tif]

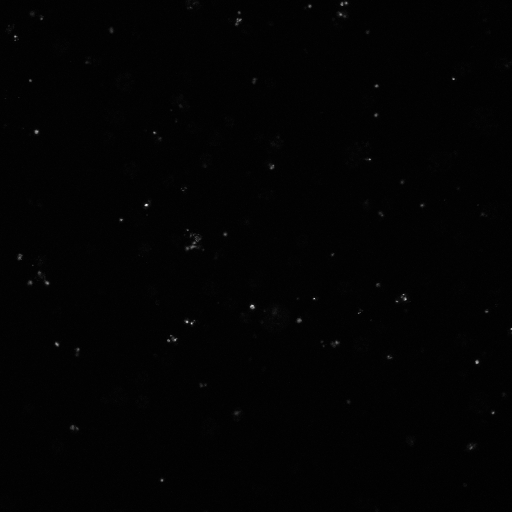

Supplement: Supplementary file 5 — Code EV1 [file MSB-13-955-s005.zip › DeathPro/example_images/150612_OC12_0h_2.1_MIPs/OC12_0h__W0024__P0002_channel1.tif]

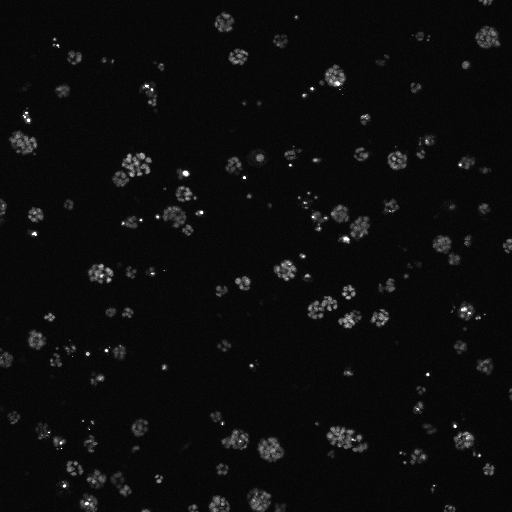

Supplement: Supplementary file 5 — Code EV1 [file MSB-13-955-s005.zip › DeathPro/example_images/150612_OC12_0h_2.1_MIPs/OC12_0h__W0025__P0001_channel0.tif]

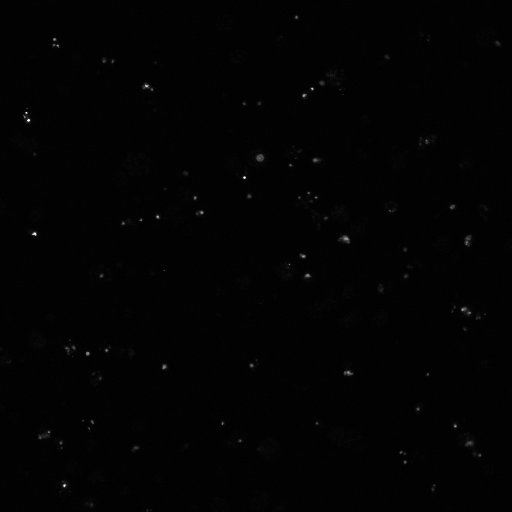

Supplement: Supplementary file 5 — Code EV1 [file MSB-13-955-s005.zip › DeathPro/example_images/150612_OC12_0h_2.1_MIPs/OC12_0h__W0025__P0001_channel1.tif]

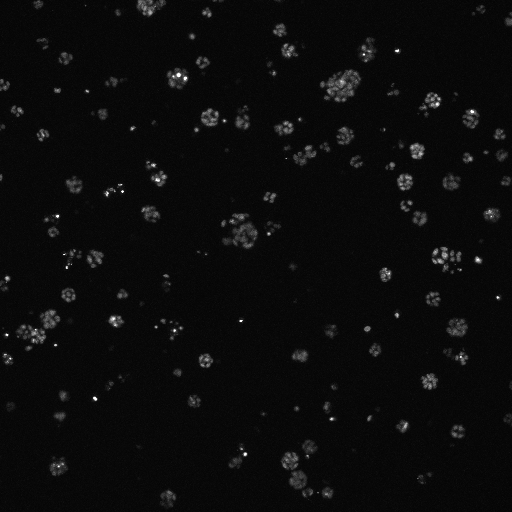

Supplement: Supplementary file 5 — Code EV1 [file MSB-13-955-s005.zip › DeathPro/example_images/150612_OC12_0h_2.1_MIPs/OC12_0h__W0025__P0002_channel0.tif]

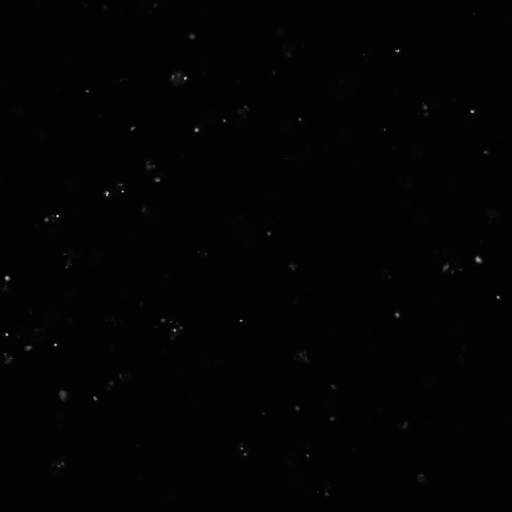

Supplement: Supplementary file 5 — Code EV1 [file MSB-13-955-s005.zip › DeathPro/example_images/150612_OC12_0h_2.1_MIPs/OC12_0h__W0025__P0002_channel1.tif]
