# Supplementary material for: Screening drug effects in patient‐derived cancer cells links organoid responses to genome alterations
Source: Mol Syst Biol. 2017 Nov 27;13(11):955. doi: 10.15252/msb.20177697 (PMC5731348; doi:10.15252/msb.20177697)
Supplement: Supplementary file 5 — Code EV1 [file MSB-13-955-s005.zip › DeathPro/example_results/150615_OC12_72h_dead_5e-04.pdf]

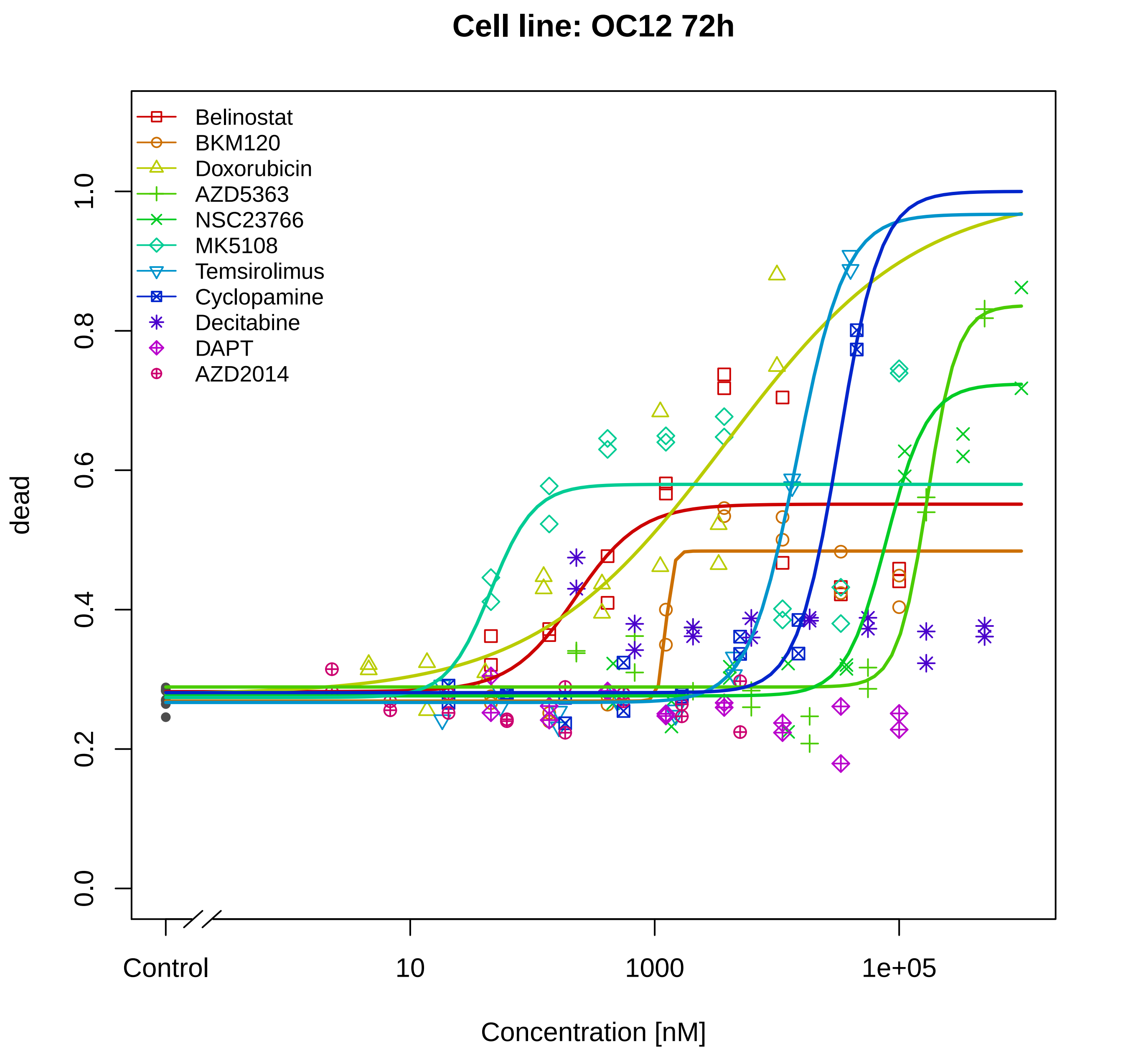

Cell line: OC12 72h
Belinostat
BKM120
Doxorubicin
AZD5363
NSC23766
MK5108
Temsirolimus
Cyclopamine
Decitabine
DAPT
AZD2014
dead
0.0
0.2
0.4
0.6
0.8
1.0
Control
10
1000
1e+05
Concentration [nM]
