# Supplementary material for: Screening drug effects in patient‐derived cancer cells links organoid responses to genome alterations
Source: Mol Syst Biol. 2017 Nov 27;13(11):955. doi: 10.15252/msb.20177697 (PMC5731348; doi:10.15252/msb.20177697)
Supplement: Supplementary file 5 — Code EV1 [file MSB-13-955-s005.zip › DeathPro/example_results/OC12_growth.pdf]

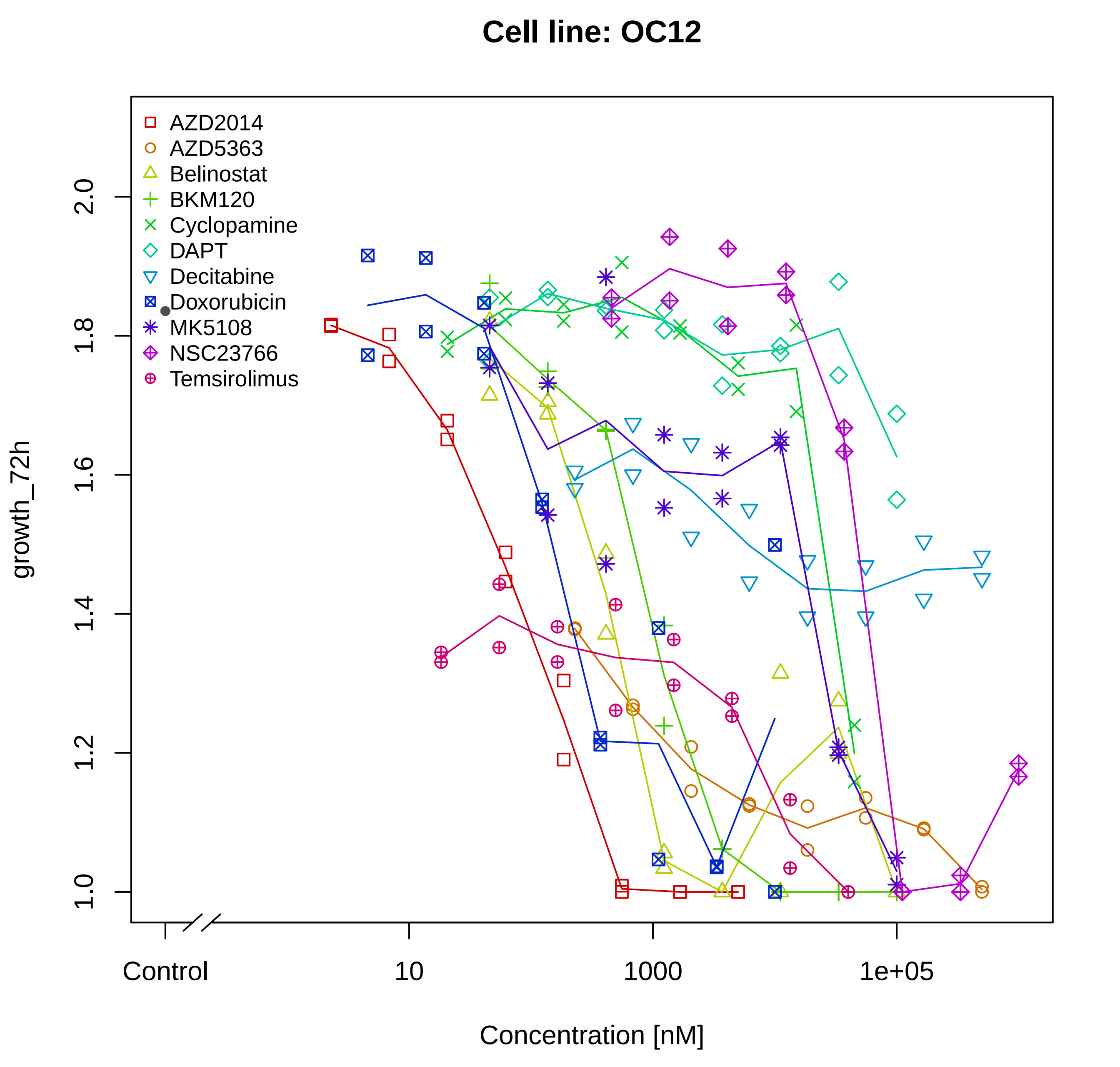

Cell line: OC12
AZD2014
AZD5363
Belinostat
BKM120
Cyclopamine
DAPT
Decitabine
Doxorubicin
MK5108
NSC23766
Temsirolimus
growth_72h
1.0
1.2
1.4
1.6
1.8
2.0
Control
10
1000
1e+05
Concentration [nM]
